# Rho-kinase inhibition reduces subretinal fibrosis

**DOI:** 10.1038/s41420-025-02709-0

**Published:** 2025-10-06

**Authors:** Yuebing Li, Tural Yarahmadov, Laura Jahnke, Tess Brodie, Sophia C. Morandi, Deborah Stroka, Ali Hafezi-Moghadam, Martin S. Zinkernagel, Volker Enzmann, Souska Zandi

**Affiliations:** 1 Department of Ophthalmology, Inselspital, Bern University Hospital, University of Bern, Bern, Switzerland; 2 Department for BioMedical Research (DBMR), University of Bern, Bern, Switzerland; 3 Graduate School for Cellular and Biomedical Sciences, University of Bern, Bern, Switzerland; 4 Department of Ophthalmology, the Affiliated Hospital of Guizhou Medical University, Guizhou Medical University, Guiyang, China; 5 Department of Visceral Surgery and Medicine, Inselspital, Bern University Hospital, University of Bern, Bern, Switzerland; 6 Department of Rheumatology and Immunology, Inselspital, Bern University Hospital, University of Bern, Bern, Switzerland; 7 Molecular Biomarkers Nano-Imaging Laboratory (MBNI), Brigham & Women’s Hospital, Harvard Medical School, Boston, MA USA

**Keywords:** Retina, Macular degeneration

## Abstract

Subretinal fibrosis, a consequence of choroidal neovascularization (CNV) in age-related macular degeneration (AMD), leads to irreversible vision loss due to excessive accumulation of extracellular matrix (ECM) proteins and fibrotic scarring. Anti-VEGF therapy can reverse neovascularization, but its effect on fibrosis is relatively limited. To reduce the visual impact of the fibrosis that remains after CNV. Our study investigated the use of ROCK inhibitors, fasudil and belumosudil, to treat subretinal fibrosis after CNV. The results confirmed that levels of key fibrotic markers (TGF-β1, fibronectin, vimentin, α-SMA and pMYPT1) were lower after treatment. IMC provided detailed spatial mapping of protein expression, revealing significant changes in structure and cellular composition before and after the treatment. We found that fasudil and belumosudil are effective in attenuating subretinal fibrosis by modulating the ROCK-signaling pathway, reducing ECM remodeling and attenuating the expression of markers associated with fibrosis. We hope to provide a basis for maximizing clinical benefit, focusing on optimizing dose and timing of treatment, exploring combination therapies for future anti-subretinal fibrosis research.

## Introduction

Subretinal fibrosis is a late-stage event associated with retinal diseases, including choroidal neovascularization (CNV) resulting from conditions like age-related macular degeneration (AMD) [1]. Critical cellular players in the context of subretinal fibrosis associated with CNV include retinal pigment epithelial (RPE) cells, glial cells, and fibroblasts [2]. Immune cells, like macrophages, have been implicated as key to the pathophysiology of subretinal fibrosis. Injection of M2 macrophages into the mouse eye aggravates the development of CNV, whereas M1 macrophages ameliorate the process [3]. The current mainstay of CNV treatment consists of the inhibition of vascular endothelial growth factor (anti-VEGF therapy). Inhibiting the PDGF pathway, which is involved in pericyte recruitment and fibrosis, has been tested in combination with anti-VEGF therapies [4, 5]. However, results are inconclusive and more research is necessary to determine the most effective strategy. Therefore, we established a mouse model without active CNV to test the anti-fibrotic effect of different compounds, such as selective Rho-kinase (ROCK) inhibitors, on fibrosis only [6].

**Fig. 1:**
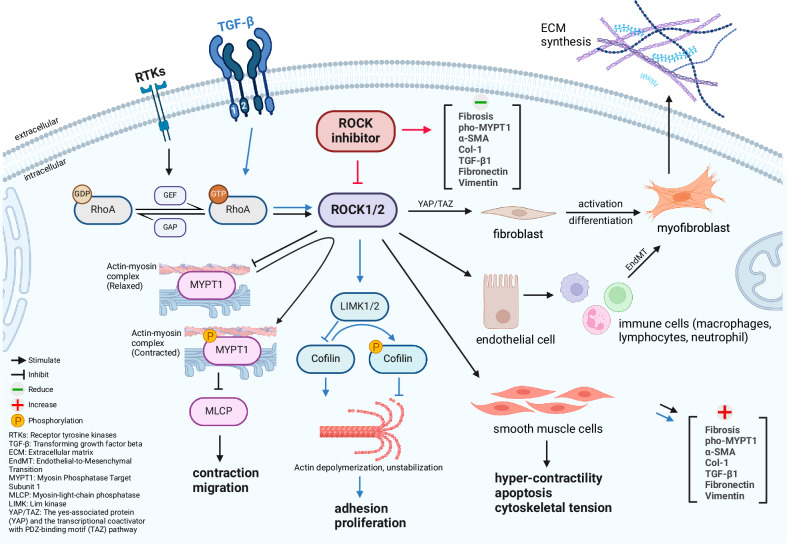
ROCK1/2 pathway diagram.

The Rho-associated coiled-coil-containing protein kinase (ROCK), a serine/threonine kinase, is an essential component of the RhoA/ROCK signalling pathway and its two isoforms ROCK1 and ROCK2 are involved in diverse cellular functions [7]. ROCK1 and ROCK2 are important players in several fundamental cellular processes, including cell contraction, migration, proliferation [8], apoptosis [9] and fibrosis. Through phosphorylation of myosin light chain (MLC), myosin phosphatase targeting protein (MYPT1) and LIM kinases (LIMKs), ROCK1 and ROCK2 play a key role in regulating cytoskeletal organization [10]. Meanwhile, local hypoxia may play a relevant role in the activation of the RhoA/ROCK pathway [11, 12]. Rho-kinase is involved in the transformation of fibroblasts into myofibroblasts, which is a key event in the development of fibrosis [13] (Fig. 1).

Rho-kinase inhibitors are a class of drugs that target one or both isoforms of Rho-associated protein kinase. By blocking the activity of the enzymes, ROCK inhibitors modify these processes to manage or treat associated diseases. Activation of ROCK promotes actomyosin contractility, leading to increased cellular tension and secretion of fibrogenic factors [14]. This in turn stimulates deposition of extracellular matrix (ECM) proteins such as collagen and fibronectin [15].

Fasudil is a synthetic small molecule, an isoquinoline derivative and a potent Rho-kinase inhibitor, acting on both ROCK1 and ROCK2. It is known for effects on vascular smooth muscle cells (VSMC), primarily through the inhibition of ROCK phosphorylating events that affect cellular function and structure [16]. Fasudil inhibits ROCK by competitively binding to the ATP-binding site in the kinase. This prevents the phosphorylation of ROCK’s downstream targets, which include MLCP and LIMKs [17, 18]. MYPT1 is a key regulatory subunit of MLCP. The phosphorylation of MYPT1 at specific sites by ROCK leads to the inhibition of MLCP activity, resulting in increased levels of phosphorylated MLC (p-MLC), which promotes muscle contraction and cell tension [19]. Through inhibition of ROCK, fasudil reduces the phosphorylation of MYPT1, thereby increasing MLCP activity. This reduces p-MLC levels, promoting muscle relaxation, with implications for reducing vascular resistance and treating conditions such as hypertension and vasospasm [20]. In addition, by modulating the activity of ROCK and its downstream targets such as MYPT1, fasudil may also have an effect on cell migration, proliferation and apoptosis relevant to the development of fibrosis [21]. However, the short half-life of fasudil and the need for intravenous injection limits its use to some extent [22]. As fasudil inhibits both ROCK isoforms, dissecting the individual contributions of each ROCK isoform during the development of subretinal fibrosis is a complex task and needs selective inhibitors, such as belumosudil.

Belumosudil (KD025) is a small molecule inhibitor of Rho-kinase, which acts primarily by selectively inhibiting ROCK2, leading to a shift in the immune response [23] and reduces fibrosis by downregulating both TGF-β signalling and profibrotic gene expression, preventing the formation of focal adhesion [24]. Belumosudil has been investigated for its potential therapeutic applications in a range of conditions including chronic graft-versus-host disease (cGVHD) and systemic sclerosis (SSc) [25]. In addition to the role of ROCK2 inhibitors in vascular injury, prophylactic and therapeutic administration of KD025 effectively attenuates thioacetamide-induced liver fibrosis and promotes fibrosis regression by disrupting signal transducer and activator of transcription 3 (STAT3)/cofilin pathways, inhibiting proinflammatory cytokine production and macrophage migration [26]. A major target of ROCK1 and ROCK2 is the regulatory subunit of myosin phosphatase, MYPT1. Phosphorylation of MYPT1 by both isoforms decreases the activity of the phosphatase, leading to higher levels of phosphorylated myosin light chain and consequently increased smooth muscle contraction [27].

By inhibiting ROCK2, belumosudil reduces the phosphorylation of MYPT1, thereby decreasing myosin light chain phosphorylation and promoting smooth muscle relaxation [28]. This would result in decreased phosphorylation of the MLC, reduced cellular contractility, and potentially beneficial effects in diseases characterized by pathological fibrosis and tissue remodelling [29]. Importantly, inhibiting ROCK2 selectively (as opposed to non-selective ROCK inhibition) may allow more targeted therapy with fewer off-target effects. Higher levels of ROCK2 in the heart and brain suggest critical roles in cardiovascular and nervous systems [7]. Conversely, ROCK1 is more prominent in the lung, liver, and spleen, hinting at isoform-specific functions in various tissues [30]. In summary, we aimed to evaluate the antifibrotic effects of ROCK inhibitors in a CNV-induced mouse model, using imaging mass cytometry (IMC) to assess changes in fibrotic signaling and extracellular matrix remodeling.

## Results

### Fibrotic signatures in human dermal fibroblasts (HDF) and mouse fibroblasts (MC3T3 cells)

**Fig. 2:**
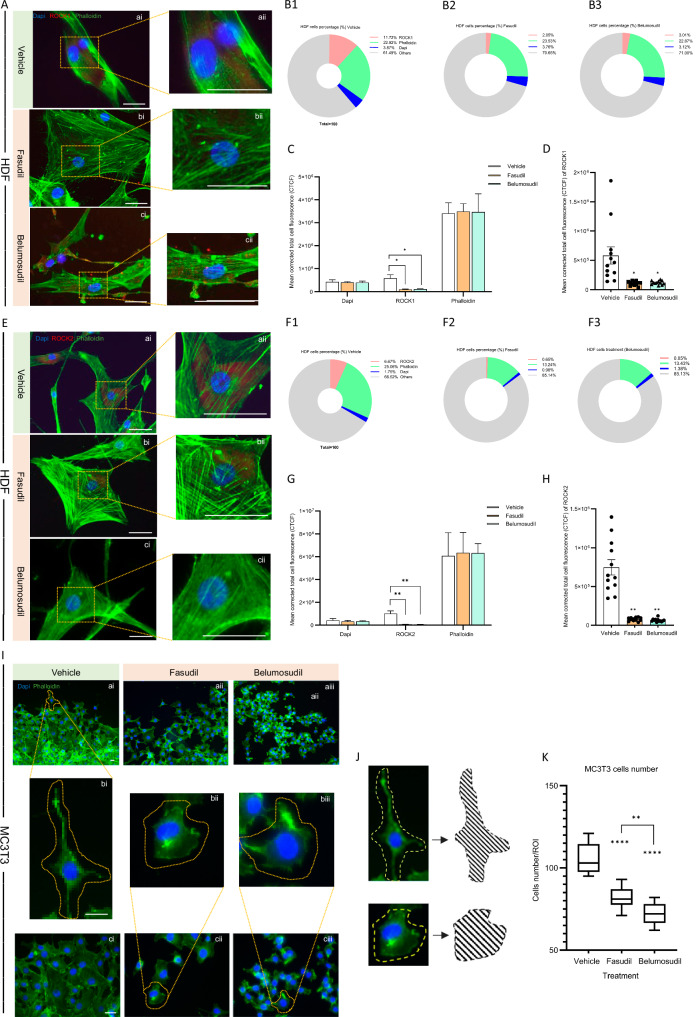
Area with ROCK1 (red) staining, corrected total cell fluorescence (CTCF) changes between before and after treatment in HDF cells. **A** Panels ai-cii depict the expression of ROCK1 in HDF prior to treatment and after ROCK inhibitors treatment. ROCK1 is identified by red staining, actin filaments are marked in green by phalloidin, and cell nuclei are stained blue with DAPI. A yellow dotted outline highlights an enlarged section of the relevant area for detailed examination. Scale bar = 50 µm. **B1**–**3** The change in percentage of cells occupying the ROCK1-stained region before and after ROCK inhibitor treatment. Red indicates ROCK1-stained regions, green denotes phalloidin staining, blue corresponds to DAPI, and gray represents background and other non-stained areas. Each pie chart is calibrated to a total of 100% to represent the entire cellular area. **C** White represents the vehicle, orange represents the fasudil group, and mint represents the belumosudil group. While the CTCF values for DAPI and phalloidin exhibited minimal, non-significant changes, ROCK1 expression decreased significantly following treatment. **D** The CTCF value of ROCK1 is significantly reduced after fasudil and belumosudil treatment. **E**, **F1**–**3**, **G**, **H** The expression and CTCF value of ROCK2 in HDF before and after treatment. **I** Changes in the number and morphology of MC3T3 cells before and after ROCK treatment. Actin filaments stained with phalloidin are green and nuclei stained with DAPI are blue. Their corresponding enlarged versions are shown in bi- biii. Scale bar = 50 µm. **J** Schematic illustration of morphology changes in MC3T3 cells. **K** MC3T3 cell number/region of interest changes after treatment.

Immunohistochemistry (IHC) was utilized to determine the expression patterns and localization of ROCK1 and ROCK2 in HDF. In the vehicle-treated group, both ROCK1 and ROCK2 were predominantly localized in the perinuclear region and cytoplasm. Following 24 h of exposure to the two distinct ROCK inhibitors, a notable decrease in the expression levels of ROCK1 and ROCK2 was observed in both fasudil-treated and belumosudil-treated groups, with a reduction in the intracellular punctate staining and some red punctate expression appearing extracellularly as depicted. Concurrently, the intracellular staining of ROCK1 and ROCK2 expression diminished. The percentage of cells occupied by ROCK1 stained regions was reduced significantly from 11.7% to 2.1% (*p* < 0.01) and 3.0% (*p* < 0.01), respectively, when compared prior to treatment with ROCK inhibitors, as detailed in Figure 2B1-3. In the assessment of ROCK2-stained regions before and after treatment with ROCK inhibitors, a marked reduction in cellular occupancy was observed. Specifically, post-treatment with fasudil, the percentage of cells with ROCK2-stained regions decreased significantly from 6.7% to 0.7% (*p* < 0.05). An even more pronounced reduction was noted with belumosudil, where the occupancy decreased from 6.7% to a mere 0.1% (*p* < 0.05; Fig. 2F1–3). Additionally, IHC analysis indicated a significant decrease in the intracellular corrected total cell fluorescence (CTCF) value for ROCK1 and ROCK2 expression following the treatment with both ROCK inhibitors. Compared with the vehicle group, it is evident that the CTCF of ROCK1 is significantly reduced in both groups (Fasudil *p* < 0.05) and (Belumosudil *p* < 0.05), meanwhile ROCK2 also significantly reduced in the fasudil-treated group (*p* < 0.01) and belumosudil-treated group (*p* < 0.01) as evidenced in Fig. 2C, D and Fig. 2G, H.

Mouse fibroblasts (MC3T3 cells) in the vehicle group displayed their characteristic morphology: an elongated spike or irregular triangular shape, with a centrally located round nucleus and 2–4 cytoplasmic protrusions of varying lengths (Fig. 2J). This morphology aligns with the cells’ typical physiological appearance, as documented in previous studies [31, 32]. After 24 h of treatment with the two ROCK inhibitors, MC3T3 cells retained their centrally located round nuclei but exhibited a blunter, rhombic periphery, with long cytoplasmic processes being diminished or absent. Concurrently, a significant decrease in cell number was noted following treatment with fasudil (*p* < 0.0001) and belumosudil (*p* < 0.0001), indicating a correlation between morphological changes as well as cell viability and treatment (Fig. 2K).

### Immunohistological assessment of fibrotic remodeling

**Fig. 3:**
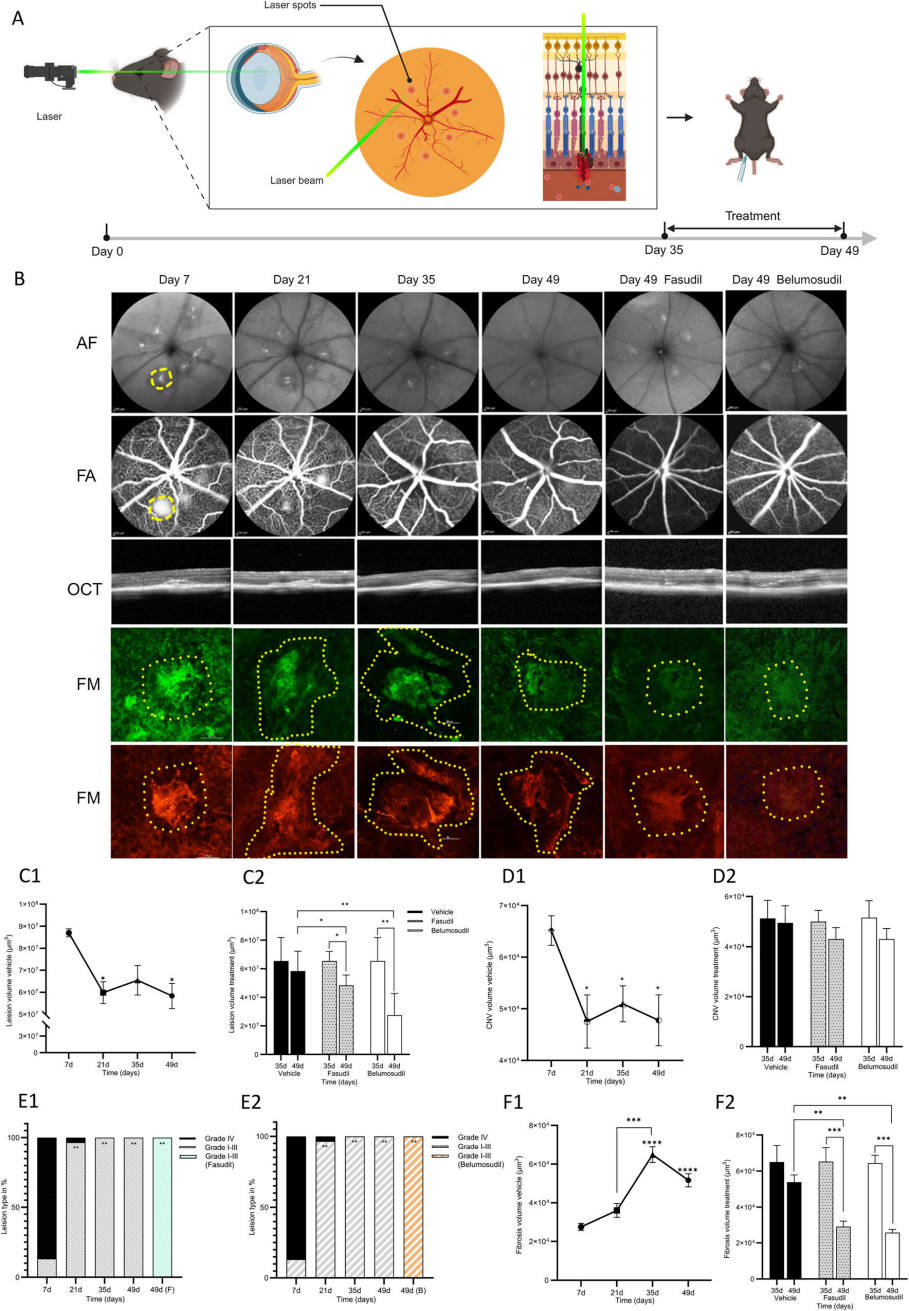
Temporal changes in laser-induced retinal lesions in mice, assessed using various imaging modalities. **A** An overview of the fibrosis model in C57BL/6 mice and the subsequent evaluation timeline. **B** Weekly serial images collected using AF, FA, and OCT from day 7 to day 49 for the vehicle group and after fasudil and belumosudil treatment. Flatmounts were stained immunohistochemically to visualize CNV and fibrosis at the same time points. Green staining for isolectin B identified CNV regions and red staining for type1 collagen highlighted areas of fibrosis. The lesions had their peripheries marked with yellow dotted lines. **C1**–**2** The volume changes in the lesion area pre- and post-treatment. **D1**–**2** CNV volume changes before and after treatment. **E1**–**2** The proportion of lesions grading classification. **F1**–**2** Fibrosis volume comparison pre- and post-treatment.

The circular laser-induced lesions were monitored weekly using fundus autofluorescence imaging (AF) from day 7 to day 49 post laser. Fluorescein angiography (FA) and Optical coherence tomography (OCT) images provided stereoscopic simultaneous sagittal and coronal views of the lesions (Fig. 3). Vehicle group lesion volumes decreased from 8.7 × 10^7^ μm^3^ and stabilized at 5.8–6.5 × 10^7^ μm^3^ from 7 to 49 days post injury (Fig. 3C1-2). Lesion components were quantified microscopically after flatmount staining with isolectin B for neovascularization (CNV) and type1 collagen for fibrosis, using Z-stack imaging for volume calculations. Notably, CNV volumes significantly differed at days 35 and 49 post-laser (*p* < 0.0001) compared to day 7 (Fig. 3D1-2). FA grading of lesions revealed that over 90% were Grade IV at day 7 post-laser, decreasing to about 5% by day 21. By day 35, all lesions (100%) were Grades I-III of leakage (Fig. 3E1-2), indicating no active CNV after 35 days post laser. Post treatment with fasudil and belumosudil, 100% of lesions remain in Grades I-III of leakage, (*p* < 0.01 for both). OCT imaging post-treatment showed a reduced lesion volume by day 49 (Fig. 3C1-2). To minimize CNV impact on therapeutic outcomes and to solely treat the subretinal fibrosis, ROCK inhibitor treatments were initiated intraperitoneally at day 35 post-laser. In the day 49 fasudil treated group, the average lesion volume decreased to 4.1 × 10^7^ µm³, from 6.3 × 10^7 ^µm³ on day 35 (*p* < 0.05), and was significantly lower than the vehicle group’s consistent volume of 6.2 × 10^7 ^µm³ on day 49 (*p* < 0.05). The day 49 belumosudil group exhibited an even more pronounced decrease, with the lesion volume dropping to 2.7 × 10^7^ µm³, from 6.2 × 10^7 ^µm³ on day 35 (*p* < 0.01), surpassing the reduction in the vehicle group (*p* < 0.0001). Fibrosis increased significantly from day 21, peaked at day 35, and then stabilized to around 5–6.5 × 10^4^ μm^3^. Fibrosis volume analysis depicted in Fig. 3F1-2 demonstrated a significant reduction in the fasudil group to 2.9 × 10^4^ µm^3^ by day 49, from 6.5 × 10^4^ µm^3^ on day 35 (*p* < 0.001), and when compared to the vehicle group 5.2 × 10^4^ µm^3^ on day 49 (*p* < 0.01). The belumosudil group exhibited an even greater decrease to 2.7 × 10^4^ µm^3^, down from 6.5 × 10^4^ µm^3^ on day 35, and notably lower than the vehicle group at day 49 (*p* < 0.001).

**Fig. 4:**
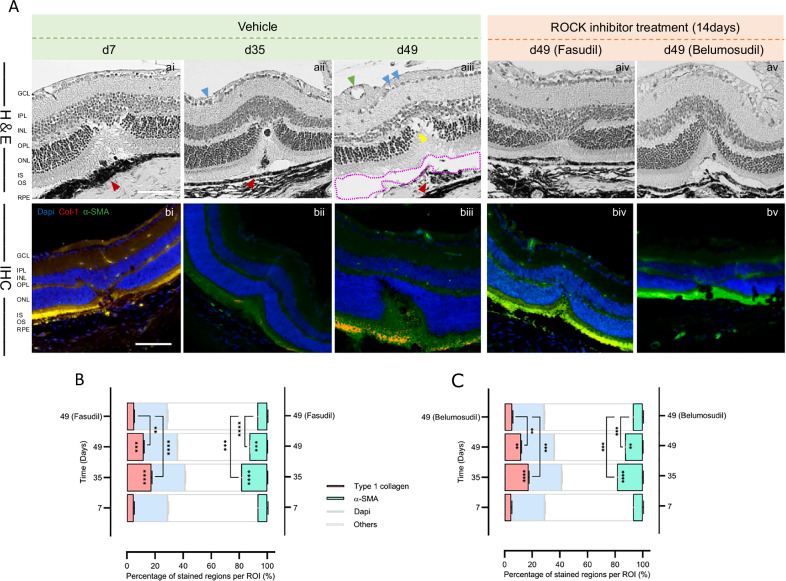
Histology (H&E) and immunofluorescent detection of type I collagen and α-SMA in mouse choroid/retina, pre- vs post-treatment. **A** Display of the temporal alterations of fibrotic makers in H&E and fluorescent staining of mouse choroid and retina paraffin sections without- and with-ROCK inhibitor treatment. Red signifies type 1 collagen, green denotes α-SMA, and blue represents DAPI staining. Scale bar = 50 µm. GCL = ganglion cell layer, IPL = inner plexiform layer, INL = inner nuclear layer, OPL = outer plexiform layer, ONL = outer nuclear layer, IS = inner segment of photoreceptors, OS = outer segment of the photoreceptors, RPE = retinal pigment epithelium. **B** The difference in the percentage of stained regions per ROI expressing type1 collagen and α-SMA between the Vehicle and Fasudil groups. **C** The difference in the percentage of stained regions per ROI expressing type1 collagen and α-SMA between the Vehicle and Belumosudil groups.

H&E staining (Fig. 4A ai–av) reveals progressive subretinal fibrosis and structural disruption in the vehicle group (aiii), while ROCK inhibitor treatment markedly preserves retinal morphology (aiv, av). Immunofluorescence for type I collagen (red) and α-SMA (green) shows increasing fibrosis and myofibroblast activation over time in untreated eyes (bi–biii), which is substantially reduced following fasudil (biv) or Belumosudil (bv) administration. (Fig. 4B–C) Quantification of stained area percentages for type I collagen, α-SMA in ROIs across timepoints, demonstrating statistically significant reductions in fibrotic and glial markers with fasudil (B) and belumosudil (C) at day 49. Following treatment with fasudil and belumosudil for 49 days, retinal thickness increased compared to the vehicle group. That included also improved cellular alignment in the INL and ONL layers, minimal intercellular spacing, and a significant decrease in pyknotic nuclei. In addition to tissue and cellular changes, changes in collagen type 1 and α-SMA expression were seen. After fasudil treatment, a significant reduction in both collagen type 1 from 11.89–5.11% (*p* < 0.01) and α-SMA from 12.76–7.12% (*p* < 0.0001) expression compared to vehicle on day 49 after injury was observed in retina and choroid (Fig. 4B). In the belumosudil group, we also found a reduction of collagen type 1 with 5.52% and α-SMA with 7.19% compared to vehicle treated mice at day 49 post laser (Fig. 4C).

**Fig. 5:**
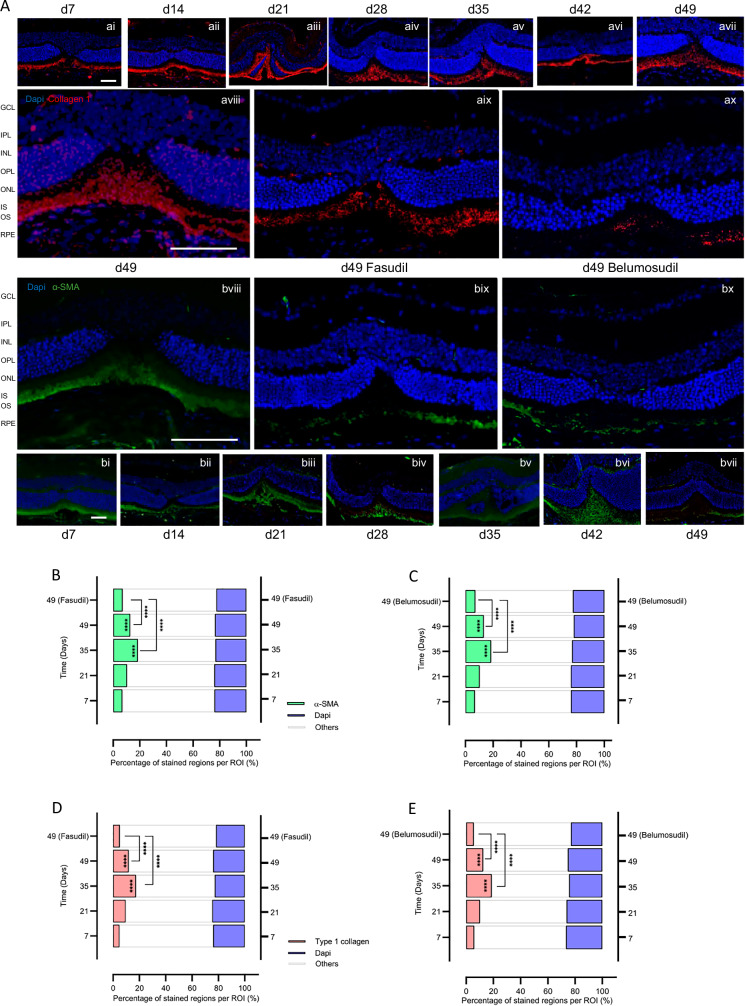
Time-course fluorescence staining of mouse choroid and retina with quantitative results. **A** Fluorescence staining images of mouse retina post-laser injury. Red indicates type1 collagen, green represents α-SMA, and blue denotes DAPI. Scale bar = 50 μm. **B**, **C** The percentage of stained regions per ROI by α-SMA. **D**, **E** The percentage of stained regions per ROI by type1 collagen. Red indicates type1 collagen, green is α-SMA, blue represents DAPI staining, and the gray- bordered blank areas denote background and other unstained regions.

To investigate fibrotic remodeling in the injured retina, we conducted immunofluorescence staining for type I collagen and α-SMA across sequential time points (days 7–49) post-injury. In the vehicle-treated group, a progressive increase in fibrotic marker expression was observed, culminating in prominent collagen deposition and α-SMA accumulation by day 49, particularly within the subretinal space and outer nuclear layer (Fig. 5A, ai–aviii, bi-bviii). In contrast, administration of the ROCK inhibitors fasudil and belumosudil from day 35 to 49 substantially attenuated the expression of both markers, suggesting effective suppression of myofibroblast activation and extracellular matrix deposition (Fig. 5A, aix–ax, bix-bx). Quantitative analyses of fibrotic and cellular markers at day 49 revealed significant attenuation of subretinal fibrosis following ROCK inhibition. Both fasudil and belumosudil treatment resulted in a marked reduction in the type I collagen positive area compared to vehicle controls (Fig. 5D,E), indicating suppression of extracellular matrix deposition. Similarly, α-SMA positive regions were significantly decreased in treated retinas (Fig. 5B,C), consistent with reduced myofibroblast activation. These findings confirm that ROCK inhibition mitigates fibrotic remodeling and supports retinal tissue integrity during late-stage degeneration.

### Western Blot validation of fibrotic markers

**Fig. 6:**
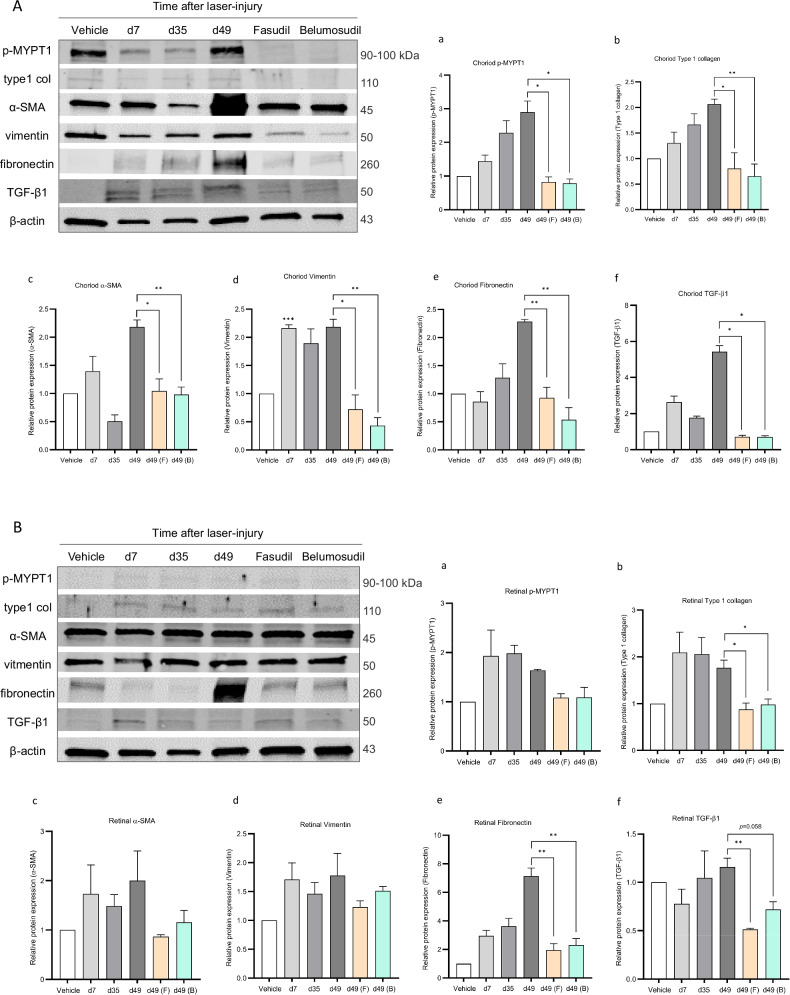
Western blot images and statistical analysis of the mouse choroid–RPE complex and retina. **A** Representative Western blot images of the mouse choroid-RPE complex. Relative expression of antibodies in the fasudil and belumosudil groups in comparison with the vehicle group at different time points. **B** Representative Western blot images of the mouse retina, relative expression of antibodies in the fasudil and belumosudil groups compared to the vehicle group at different time points.

The expression of myosin phosphatase target subunit 1 (p-MYPT1), the major substrate of ROCK was significantly reduced the choroid-RPE complex (Fig. 6A) following the administration of both inhibitors (*p* < 0.05). Expression of type1 collagen, a key fibrotic marker and extracellular matrix component, peaked between days 35 and 49 after laser in the vehicle group. After treatment, belumosudil significantly decreased type1 collagen levels (*p* < 0.01), with fasudil also showing a similar trend (*p* < 0.05). Myofibroblast activation, as indicated by α-SMA expression, reached a peak twice on days 7 and 49 after injury in the vehicle group, then significantly attenuated by both ROCK inhibitors (*p* < 0.01). Similarly, fibronectin, a glycoprotein involved in fibrosis, increased until day 49 in the vehicle group but showed a significant reduction after treatment with both fasudil and belumosudil (*p* < 0.01). Vimentin, a protein associated with wound healing and scarring processes, showed high expression at day 7 and peaked at day 49 after laser induction, consistent with previous observations [6]. Notably, vimentin expression followed a decreasing pattern after treatment with both ROCK inhibitors, with fasudil (*p* < 0.05) and belumosudil (*p* < 0.01) showing reductions. Furthermore, TGF-β1, a multifunctional cytokine that regulates cell proliferation and differentiation and plays a major role in fibrosis, showed an upsurge early at day 7 after injury, a further peak at day 49 post-laser in the vehicle group, and a between days 35–49 decrease following treatment with both fasudil and belumosudil (both *p* < 0.05).

In mouse retina, Western blot results (Fig. 6B) indicate both type1 collagen and TGF-β1 levels were lower in the fasudil group compared to vehicle (*p* < 0.05 for both), with similar decrease observed in the belumosudil group for type1 collagen (*p* < 0.05) and TGF-β1 (*p* = 0.058). Noteworthy was the significant reduction of fibronectin expression post-treatment with either ROCK inhibitor (*p* < 0.01). These results suggest that the ROCK inhibitors exert their anti-fibrotic effects by modulating key molecules involved in the fibrotic process, confirming their potential as therapeutic agents to mitigate subretinal fibrosis.

### Cell type classification and spatial clustering by IMC

**Fig. 7:**
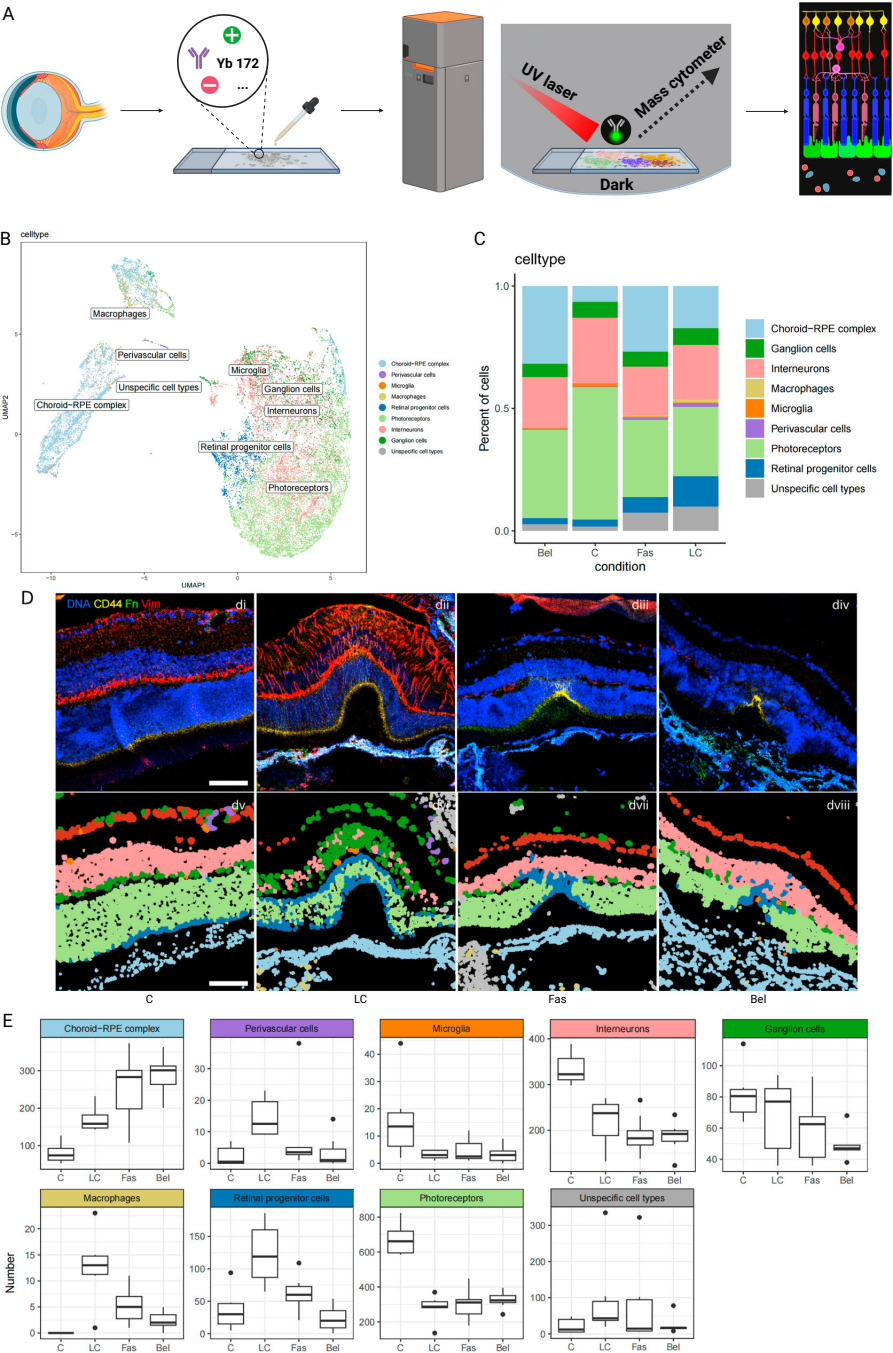
IMC-based retinal cell profiling and antibody detection dynamics pre- and post-ROCK inhibition. **A** IMC flow chart. **B** UMAP visualization of cell types in the mouse retina. **C** Comparative analysis of antibody detection ratios across various cell types pre- and post-Rock inhibitor treatment. Different colors correspond to the annotations in **B**. **D** Panels di-div depict the expression distribution images of different antibodies in the retina of normal healthy mouse by IMC, they represent in order: C, LC, Fas, Bel groups. Blue-DNA, yellow-CD44, green-fibronectin, red-vimentin. Panels dv-diii are the corresponding images of di-div. **E** Variations in cell type distribution in the retina pre- and post-Rock inhibitor treatment. C-Control, LC-Laser control, Fas-Fasudil, Bel-Belumosudil. Different colors correspond to the annotations in **B**, **C**. Scale bar = 60 µm.

The heatmap and UMAP illustrate variance in antibody expression across cell types (Fig. 7B, C). Data were processed as described in the Steinbock pipeline, except for cell segmentation masks being produced using the cellpose tissue net model (Fig. 7D). Thereby, α-SMA selectively marks perivascular cells, distinguishing itself as the sole antibody in this panel. In contrast to α-SMA’s specificity for perivascular cells, CD44 and beta-catenin preferentially label retinal progenitor cells and demonstrate concentrated expression in regions marked by laser spots. In addition, the expression of vimentin is also closely related to macroglia. The number of retinal ganglion cells in the healthy mouse vehicle group was significantly higher than in the laser-only group (*p* < 0.001). Treatment with ROCK inhibitors makes no significant changes in ganglion cell numbers compared to laser-only group. As same as photoreceptors. After treatment, there was a highly significant increase in the number of interneurons in both the Fasudil (*p* < 0.01) and Belumosudil (*p* < 0.01) groups compared to the laser-only group. The number of laser-only group macroglia increased significantly (*p* < 0.05) after laser damage. However, Fasudil treatment led to a significant decrease (*p* < 0.01), and this reduction was even more pronounced with Belumosudil treatment (*p* < 0.001). In the vehicle group, only a small number of macrophages were present. Following injury, the number of macrophages increased over time. Treatment with Fasudil significantly reduced the number of macrophages (*p* < 0.05) compared to group laser-only. Similarly, Belumosudil treatment also led to a reduction in macrophage numbers (*p* < 0.05). Perivascular cell counts were significantly higher in the laser-only group compared to the Control group (*p* < 0.05). After treatment with ROCK inhibitors, there was a reduction in cell numbers in the Belumosudil group compared to the laser-only group (*p* = 0.053). Retinal progenitor cell counts in the laser-only group significantly increased compared to the Control group (*p* < 0.05). Following ROCK inhibitor treatment, the Belumosudil group showed a significant decrease in cell numbers compared to the laser-only group (*p* < 0.05). After laser-induced damage, there was a slight decrease in microglia cells (*p* = 0.059); however, no significant increase was observed following treatment with two ROCK inhibitors (Fig. 7E). Although the total number of choroid-RPE complex cells did not change significantly before and after treatment, it is worth noting that the presence of fibrosis-related markers such as type1 collagen and fibronectin in Choriod-RPE complex suggests the formation of secondary subretinal fibrosis following laser-induced injury. Quantitative analysis revealed a significant increase in cellular density within this fibrotic region by day 49 post-laser. Interestingly, after treatment with BEL, the number of cells expressing type1 collagen (*p* < 0.001), vimentin (*p* < 0.001), and fibronectin in this region was reduced compared to laser control group (supplementary tables/files Fig. 1). A similar situation was also observed in the Fas treatment group. However, treatment with ROCK inhibitors notably reduced the number of cells expressed fibrotic markers in this area, with belumosudil demonstrating a more pronounced effect compared to fasudil.

We observed a population of unspecific cell types (gray area in Fig. 7B,C,E) characterized by unique morphology and size, differing notably from other cell groups. The LC and Fas groups exhibit significantly elevated median expression levels and broader variability, including notable outliers, indicative of enhanced fibrotic activity. In contrast, the C and Bel groups display comparatively low expression. These cells express fibrotic markers such as collagen and fibronectin and are primarily localized in the choroid–RPE layer. However, their morphology deviates from that of typical RPE cells. We hypothesize that these regions may represent areas of fibrotic tissue accumulation. This cell population may consist of fibroblasts, fibrotic tissue, or RPE cells that have undergone degeneration, potentially via epithelial–mesenchymal transition (EMT).

## Discussion

Subretinal fibrosis, a sequela of exudative AMD, remains one of the leading causes of irreversible vision loss [33]. This condition is characterized by an excessive accumulation of ECM proteins and the formation of fibrotic scars within the subretinal space, which disrupt the normal retinal architecture and thus impede visual function.

In human fibroblasts (HDF), the immunohistochemical localization and expression of ROCK1 and ROCK2 isoforms offer a window into the subcellular shifts that underpin fibrotic processes. The significant reduction in ROCK expression upon treatment with both fasudil and belumosudil indicates a direct impact of these inhibitors on the ROCK-signalling pathway. The decline in CTCF post-treatment suggests a decrease in ROCK activity, corroborating the hypothesis that ROCK inhibitors can effectively modulate fibroblasts. The observed extracellular puncta of ROCK isoforms following treatment may indicate a release mechanism, which warrants further investigation to understand its implications in the context of cellular signalling and fibrosis mitigation.

The MC3T3 mouse fibroblast cell line displayed altered morphology and reduced proliferation when subjected to ROCK inhibitors. The blunting of cellular processes and decreased cell numbers post-treatment underscore the potential for ROCK inhibitors to suppress the phenotypic attributes that contribute to fibrosis.

The utilization of fundus AF, FA, and OCT provided a multimodal assessment of retinal and choroidal lesions. Vehicle-treated groups demonstrated a progression of lesion size over time, with volumes decreasing and stabilizing between days 7 and 49 post-laser. This progression reflects the inherent wound-healing response of retinal tissues following injury. The initial volume reduction is presumably attributed to the cessation of acute inflammatory and exudative processes that characterize the early post-injury phase. The FA grading system revealed a rapid evolution of leakage severity, with most reaching Grade IV early post-laser and then regressing to lower grades, suggesting a transition from an active CNV to a scar phase within three weeks. By day 35, lesions show an increase in fibrotic scar formation, indicting a predominantly healing rather than active inflammatory state.

Treatment with ROCK inhibitors, fasudil and belumosudil, at day 35 post-laser yielded significant improvements in lesion size by day 49, with a noteworthy decrease in lesion volumes observed via OCT and confocal microscopy. This observation aligns with the hypothesized role of ROCK inhibitors in modulating wound healing and fibrotic processes. ROCK inhibition has been implicated in cytoskeletal rearrangement, cell migration, and myofibroblast activation - key elements in the pathogenesis of subretinal fibrosis [34]. Fasudil’s effect on fibrosis volume was significant but less so than belumosudil, which could be due to differences in drug potency, bioavailability, or the specific ROCK isoforms inhibited. The marked decrease in fibrosis volumes post-treatment underscores the potential of ROCK inhibitors in altering the extracellular matrix-remodelling characteristic of subretinal fibrosis. The reduction of subretinal fibrosis lesions after treatment with fasudil and belumosudil not only supports their therapeutic efficacy but also underscores the role of ROCK signalling in subretinal fibrosis pathology. The stabilization of fibrosis volumes after peak suggests a potential window during which intervention with ROCK inhibitors could be optimally effective.

Histological analyses painted a vivid picture of the in vivo consequences of ROCK inhibition. A significant reduction in both type1 collagen and α-SMA expression after treatment indicates a dampening of fibrotic activity at the tissue level. These markers, indicative of myofibroblast presence and ECM remodelling, are cornerstones in the establishment of fibrosis. The observed decreases in expression align with the anticipated outcomes of ROCK inhibition namely, reduced myofibroblast transdifferentiation and ECM production.

In vitro, the ROCK inhibitor Y27632 blocks TGF-β–induced type1 collagen expression in human retinal pigment epithelial cells. Consistent with this, fasudil has been shown to downregulate α-SMA and type1 collagen levels in fibroblasts and animal models of fibrosis. Likewise, belumosudil suppresses TGF-β1–induced fibroblast activation and ameliorates fibrotic remodelling in the heart. In our CNV-fibrosis model, both agents dramatically reduced collagen deposition and α-SMA expression, indicating that pan-ROCK (fasudil) and ROCK2-selective (belumosudil) blockade can effectively interrupt the profibrotic cascade. Notably, the similar efficacy of belumosudil highlights the particular importance of ROCK2 in driving fibrosis, likely via modulation of TGF-β/Smad pathways.

The Western blot analyses further cement the role of ROCK inhibitors in attenuating fibrotic signalling. Fasudil and belumosudil, by inhibiting different ROCK isoforms, can alter the course of fibrotic processes [24, 35]. The expression levels of certain proteins, including type1 collagen, TGF-β1, fibronectin, vimentin and α-SMA are markers that serve as indicators of fibrotic activity and provide measurable endpoints to assess the efficacy of ROCK inhibitors in a disease context. Type1 collagen and TGF-β1, both central to the development of fibrosis, showed decreased levels in the fasudil group, with belumosudil demonstrating a similar trend. Thereby, type1 collagen is a primary structural component of the ECM, and its overexpression is a hallmark of fibrotic tissue [36]. On the other hand, TGF-β1 is a key cytokine involved in the proliferation of fibroblasts and the transformation into myofibroblasts, the primary cells responsible for ECM deposition [37]. The reduction in these proteins is particularly encouraging, pointing towards the suppression of fibrotic processes by ROCK inhibition. Fibronectin, another critical ECM protein, was also reduce following treatment with both inhibitors. Fibronectin plays an essential role in cell adhesion and matrix formation [38]; its reduced expression suggests a possible decrease in matrix deposition and fibroblast activity. The expression of vimentin, which peaked early post-induction of the model, followed by a significant decrease after ROCK inhibitor treatment, correlates with the protein’s role in early wound healing and fibrosis [39]. This response underscores the potential restorative effects of ROCK inhibition on retinal and choroidal architecture post-injury. α-SMA, a marker for myofibroblast activation [40], also demonstrated a significant reduction following treatment with ROCK inhibitors. The attenuation of α-SMA levels suggests a decline in myofibroblast presence, which is crucial given their role in the synthesis and contraction of fibrotic tissue. The downregulation of α-SMA provides a strong indication of the therapeutic value of ROCK inhibitors in controlling the fibrotic response. Furthermore, p-MYPT1 serves as a downstream effector in the ROCK pathway and inhibitors of ROCK, such as fasudil and belumosudil, may reduce the phosphorylation of MYPT1 by inhibiting ROCK, thereby increasing the activity of MLCP, reducing MLC phosphorylation, and ultimately decreasing cell contractility [41]. The expression of p-MYPT1 in the choroidal-RPE complex was significantly reduced following administration of both ROCK inhibitors. This mechanism is part of the rationale behind the use of ROCK inhibitors as potential therapies for conditions involving aberrant contractility and fibrosis. In the choroid-RPE complex, the downregulation of p-MYPT1, vimentin, and α-SMA following treatment suggests that ROCK inhibition may be impeding the fibrotic cascade at multiple levels, including the inhibition of myofibroblast activation, ECM component and profibrotic cytokine synthesis.

The IMC method was applied for the first time to detect the antibody expression levels of various cell types in a mouse model of subretinal fibrosis. The choroid-RPE complex, known for its critical function in supporting retinal physiology through nutrient supply and waste removal [42], might exhibit a heightened immune response or cellular activity, as evidenced by the universal marker upregulation. The selective marking of peripheral cells by α-SMA is a notable finding, establishing α-SMA as a unique marker for these cells within the panel. This specificity together with our findings in Western blot suggest that peripheral cells possess a distinct cytoskeletal or contractile feature that is critical for their function or identity. In contrast, the preferential labeling of retinal progenitor cells by CD44[43] and beta-catenin [44], especially in regions affected by laser treatment, indicates a potential role for these cells in the response to retinal injury or in the process of retinal regeneration. This is further corroborated by the focused expression of these proteins in areas with laser spots, hinting at a localized upregulation in response to damage or stress. Vimentin’s association with macroglia underscores its relevance in glial cells’ structural integrity and reactive changes [45], particularly under pathological conditions. The significant disparities in cell type distribution between the control and laser control groups highlight the laser’s impact on the cellular composition of the retina. This effect underscores the sensitivity of retinal cells to stress, reflecting changes in cell populations that are attributed to cell death, migration, or proliferation in response to injury.

The treatment effects of fasudil and belumosudil (on retinal cells offer intriguing insights into their therapeutic mechanisms. The significant increase in choroid-RPE complex, and interneurons after fasudil treatment could indicate a protective or regenerative effect, possibly by enhancing cellular resilience or promoting regeneration. Conversely, the notable decrease in macroglia following this treatment suggests a reduction in glial reactivity or proliferation, which could be beneficial in mitigating retinal inflammation or fibrosis. The belumosudil group’s pattern closely mirrors that of the fasudil group, with the added effect of significantly reducing macrophages and perivascular cells. This reduction may reflect a targeted anti-inflammatory or anti-fibrotic action, pointing to belumosudil’s potential in specifically modulating the immune response and fibrotic components of the retina. The observed changes after belumosudil treatment, especially the diminished numbers of macrophages and perivascular cells, could have implications for treating eye diseases characterized by inflammation and fibrosis.

### A fibrotic niche hypothesis

Interestingly, Fig. 7E shows that the boxplot reveals significant expression of fibrotic markers, namely collagen and fibronectin, within a population of unspecific cells across laser conditions (LC), and reduced after treatment with belumosudil. These cells are localised in or near the choroid–RPE boundary and demonstrate morphological deviation from canonical RPE phenotypes. Their anatomical positioning and expression profile of fibrotic markers suggest that they are not merely residual cell types, but rather an actively engaged, heterogeneous cellular population. Recent insights into fibroblast biology underscore their remarkable heterogeneity and plasticity across tissues and disease states. Fibroblast identity is shaped by four key factors: tissue condition, regional anatomy, microenvironmental signals, and cell state [46]. In fibrotic conditions, quiescent fibroblasts can become activated, differentiate into myofibroblasts, and express fibrogenic markers such as α-SMA, collagen, and fibronectin—consistent with the marker profile seen in the unspecific IMC population.

Moreover, myofibroblasts may originate not only from resident fibroblasts, but also from other cell types, such as pericytes, mesenchymal progenitors and RPEs undergoing EMT. This flexibility supports the hypothesis that some of the observed cells could be fibroblast-like cells that arise from local precursors rather than forming part of a homogeneous fibroblast lineage. An alternative, though not mutually exclusive, interpretation is that these non-specific cells are degenerating RPE cells undergoing EMT. EMT is a cellular reprogramming process whereby epithelial cells lose their polarity and junctional integrity, gaining mesenchymal characteristics such as motility and ECM production. This transition is well established in ocular pathologies, including proliferative vitreoretinopathy and advanced AMD. The single-cell RNA sequencing demonstrating extensive remodelling of cell types in an NaIO₃-induced dry AMD model. While much attention was focused on Sox2⁺ Müller glia and their loss via ferroptosis and apoptosis, the dataset also revealed a dramatic reduction in RPE cell populations, as well as the emergence of fibroblast-like gene expression signatures in the retina [47]. The expression of fibrosis-associated markers, especially in the choroid–RPE region, could thus reflect EMT-mediated transformation of RPE cells into mesenchymal phenotypes. Additionally, in pathological contexts such as AMD, oxidative stress, lipid accumulation, and chronic inflammation serve as EMT-inducing stimuli [48]. EMT potentially coupled with ferroptotic stress and pro-inflammatory cytokines, drives some RPE cells toward a fibroblast-like, fibrogenic fate [49].

Rather than being distinct entities, it is increasingly plausible that fibroblasts, myofibroblasts, and EMT-derived RPE cells co-exist within a pathological niche characterized by oxidative stress, immune infiltration, and matrix remodeling. These populations may not only share functional characteristics but also exhibit phenotypic overlap, forming a fibrotic continuum. In this context, the “choroid-RPE complex and unspecific” cell population may represent a transitional or mixed lineage state. Furthermore, lineage tracing studies in other organ systems have shown that once cells transition to a fibrotic phenotype, they can maintain their activated state through epigenetic imprinting, thus becoming persistent drivers of tissue remodeling. In ocular fibrosis, this implies that EMT-derived RPE cells may not revert even if the stimulus is removed, potentially contributing to irreversible retinal degeneration.

The distinct roles of α-SMA, CD44, type1 collagen and vimentin in marking specific cell populations, alongside the therapeutic impacts of fasudil and belumosudil, underscore the complexity of retinal biology and offer avenues for targeted interventions in retinal pathologies. Typically, interneurons and ganglion cells do not regenerate after injury. It is aligned with our results. Notably, in terms of the effects on certain cells, such as macrophages/microphages, retinal progenitor cells, perivascular cells and fibrotic related cells in choroid-RPE complex, the decrease in nonspecific cell numbers was more pronounced in the belumosudil-treated group compared to fasudil. This may reflect the distinct inhibitory potency of ROCK2-selective blockade on cellular infiltration and proliferation in fibrotic tissue. Since ROCK2 is closely linked to TGF-β1 signaling, which drives fibroblast activation, immune cell recruitment, and extracellular matrix remodelling, selective ROCK2 inhibition may more effectively suppress the cellular mechanisms contributing to fibrotic progression. These findings highlight the potential of belumosudil as a targeted antifibrotic agent, particularly in modulating the cellular microenvironment of subretinal fibrotic lesions.

### Limitations and future directions

The laser-induced CNV mouse model has its limitations to fully replicate the human AMD and fibrotic response. While the model is widely used, results must be extrapolated to human AMD with caution, as certain chronic pathological processes and the influence of aging are not represented. To identify the cells responsible for scar formation in our lesions, the “unspecific” cell type and population at the choroid–RPE boundary exhibited fibrotic markers and atypical morphology, suggesting a mix of fibroblasts, myofibroblasts, or RPE-derived mesenchymal cells. However, without cell-specific markers or lineage tracing, this interpretation remains speculative. It is known that multiple cell types (RPE, macrophages, pericytes, endothelial cells) can undergo mesenchymal transition into myofibroblasts in subretinal fibrotic lesions. In future, we will refine our approach by using single-cell RNA sequencing, lineage tracing, and EMT-specific markers to clarify the origins and roles of these fibrotic cell populations.

## Conclusion

The convergence of cellular, histological, and molecular data presented in this study illuminates the potent anti-fibrotic effects of ROCK inhibitors in both in vitro and in vivo. By dissecting the levels of fibrotic pathology, our evidence supporting the repurposing of ROCK inhibitors as a viable strategy against subretinal fibrosis. The observed reductions in key fibrotic markers, coupled with the partial restoration of retinal architecture, offer a promising avenue for addressing fibrosis-driven vision impairment. Nevertheless, the path to clinical application is intricate. Given the close association between ROCK2 and TGF-β1 signaling—central to fibroblast activation, immune cell recruitment, and extracellular matrix remodeling—selective inhibition of ROCK2 may yield more precise and effective suppression of fibrotic progression. Our findings thus highlight belumosudil’s potential as a targeted anti-fibrotic agent, particularly in reshaping the pathological microenvironment of subretinal fibrotic lesions.

The morphological features, anatomical context, and fibrotic markers expression signatures of the unspecific cells observed in fibrosis formation period strongly support the hypothesis that this population comprises a heterogeneous mix of fibroblasts, myofibroblasts, and RPE-derived cells undergoing EMT. Future investigations using lineage tracing, spatial transcriptomics, and EMT marker panels would be crucial to definitively resolve the ontogeny and functional roles of these cell types. These findings highlight the potential of belumosudil as a targeted antifibrotic agent, particularly in modulating the cellular microenvironment of subretinal fibrotic lesions. The realization of their full therapeutic potential will hinge on the integration of these preclinical findings with patient-centric research, wherein individual variations in response can be accounted for and treatment can be tailored accordingly.

## Materials and methods

### Laser-induced CNV and subretinal fibrosis

Mice were anaesthetized and pupils were dilated for laser. Six spots were placed in each eye using a 532 nm argon laser (Visulas532s; Carl Zeiss Meditec AG, Oberkochen, Germany), a slit delivery system (Iridex Corporation, Mountain View, CA) mounted on a slit lamp (BM 900; Haag-Streit AG, Köniz, Switzerland), and a coverslip as a contact lens.

### Cell culture

Human dermal fibroblasts (HDF; C-12302, Sigma-Aldrich) and mouse fibroblasts (MC3T3 cells; CRL-2593, ATCC, Manassas, VA, USA) were cultured under 5% CO_2_ at 37 °C. Cells were then incubated with only medium (vehicle group), medium with fasudil (fasudil group) or with belumosudil (belumosudil group) for 24 h and fixed with 4% paraformaldehyde (PFA) after treatment for 20 min.

### FA, AF and OCT

Autofluorescence imaging (AF), fluorescein angiography and Optical coherence tomography (OCT) were performed through a digital fundus camera (SLO; Heidelberg Engineering, Heidelberg, Germany) at all designated time points after laser injury. The retinal pigment epithelium (RPE)-choroid-sclera complex was flat-mounted.

### ROCK inhibitor treatments

Fasudil (LKT-F0275; Biomol, St. Paul, MN, USA) and belumosudil (SLx-2119, KD-025; S7936; Selleck Chemicals, Houston, USA) were used as ROCK inhibitors. Both were administered intraperitoneally (i.p.) from d35 to d49 post laser.

### H&E staining and immunohistochemistry

Eyes were fixed in 4% PFA solution at 4 °C for 24 h. Paraffin sections (5 µm) of the posterior segment were prepared and stained with haematoxylin and eosin (H&E). For immunohistochemistry, the slides were incubated with the first antibodies and followed by incubation with second antibodies, respectively.

### Western Blot

For Western blot, 10 laser spots were placed in each eye. An additional washing step of 10 min with Stripping Buffer (NewBlot Nitro; D20815-03; LI-COR, Bad Homburg, Germany) was added between the incubations of each antibody.

### IMC

Paraffin sections (2–3 µm) from the posterior segment of the mouse eye were stained following immunohistochemistry protocols, utilizing antibodies labeled with heavy metal isotopes. These stained samples were analysed using the Hyperion Imaging System, which ablates tissue with a laser and uses TOF mass cytometry for single-cell resolution imaging. Data were visualized with Napari v.0.4.15, processed using the Steinpose Napari plugin, and analysed with R v.4.3.0 in RStudio, following the “IMC Data Analysis Workflow.” Additional details regarding the materials and methods are reported in the supplementary files.

### Statistical and analysis

All experiments were conducted in triplicate. Data, normally distributed per Bonferroni’s test, were analysed using Student’s *t*-test or ANOVA and expressed as mean ± SEM. Significance was noted as *p < 0.05, **p < 0.01, ***p < 0.001, and ****p < 0.0001. GraphPad Prism, Fiji, Qupath, Inkscape, and R were used for analysis and illustration. Figures 1, 3A, and 7A were designed and created using BioRender. Grammarly and DeepL were used for grammatical correction and polishing.

## Supplementary information

Supplementary tables and files

Additional Materials and Methods

Supplementary-westernblot original files

## Data Availability

All raw MCD data, the segmentation masks and parameters used to obtain them, the R object we obtained after the analysis, and the analysis script required are in Zenodo at the following link: https://zenodo.org/records/16780397.
