# Supplementary material for: Rho-kinase inhibition reduces subretinal fibrosis
Source: Cell Death Discov. 2025 Oct 6;11:428. doi: 10.1038/s41420-025-02709-0 (PMC12501381; doi:10.1038/s41420-025-02709-0)
Supplement: Supplementary file 1 — Supplementary tables and files [file 41420_2025_2709_MOESM1_ESM.docx]

| **Contents** |  |  |  |  |  |  |  |  |  | page |
| --- | --- | --- | --- | --- | --- | --- | --- | --- | --- | --- |
| **S1** | **Table S1. Full list of abbreviations** | | | | | | | | | 2-4 |
| **S2** | **Table S2. Hyperion Imaging System protein markers** | | | | | | | | | 5-7 |
| **S3** | **Table S3. Hyperion cells anntotated methods** | | | | | | | | | 8 |
| **S4** | **Table S4. Statistical analysis (Bonferroni's multiple comparisons test and two-way ANOVA)** | | | | | | | | | 9-22 |
| **S5** | **Table S5. Comparison between groups (one-way ANOVA)** | | | | | | | | | 23 |
| **S6** | **Table S6. SEM and p value of the t-test in Figure 6** | | | | | | | | | 24-27 |
| **S7** | **Table S7. DA results donor of Figure 7** | | | | | | | | | 28-30 |
| **S8** | **Table S8. Totall cell numbers** | | | | | | | | | 31-33 |
| **S9** | **Table S9. IMC sample metadata** | | | | | | | | | 34-35 |
| **S10** | **Table S10. Celltype neighborhoods** | | | | | | | | | 36-53 |
| **S11** | **Table S11. Expression neighborhoods** | | | | | | | | | 54-71 |
| **S12** | **Table S12. IMC differential state** | | | | | | | | | 72-83 |
| **S13** | **Table S13. Antibody list** | | | | | | | | | 84 |
| **F1** | **Figure F1. Boxplot of fibrosis-related markers in IMC** | | | | | | | | | 85 |

**Supplementary tables/files**

| **Table S1. Full list of abbreviations** | | |
| --- | --- | --- |
| **Group** | **Abbreviation** | **Unabbreviated form** |
| Diseases & Signs | CNV | choroidal neovascularization |
|  | AMD | age-related macular degeneration |
|  | nAMD | neovascular age-related macular degeneration |
|  | cGVHD | chronic graft-versus-host disease |
|  | SSc | systemic sclerosis |
|  | CC | congested choriocapillaris |
| Celluar components and proteins | ECM | extracellular matrix |
|  | BM | bruch's membrane |
|  | MLC | myosin light chain |
|  | MYPT1 | myosin phosphatase targeting protein |
|  | LIMKs | LIM kinase |
|  | MLCP | myosin light chain phosphatase |
|  | p-MLC | phosphorylated MLC |
|  | STAT3 | signal transducer and activator of transcription 3 |
|  | pMYPT1 | myosin phosphatase target subunit 1 |
| Cell types | RPE | retinal pigment epithelial |
|  | VSMC | vascular smooth muscle cells |
|  | HDF | human dermal fibroblasts |
|  | MC3T3 cells | mouse fibroblasts |
| Growth factors | TGF- β | transforming growth factor-β |
|  | PDGF | platelet-derived growth factor |
|  | VEGF | vascular endothelial growth factor |
| Cytokines | IL-6 | interleukin-6 |
|  | IL-1 | interleukin-1 |
|  | TNF-α | tumour necrosis factor-alpha |
|  | GEFs | guanosine exchange factors |
| Cell activities | EMT | epithelial-mesenchymal transition |
| Rho-kinase | ROCK | rho-kinase |
|  | ROCKs | rho-associated coiled-coil-containing protein kinases |
|  | Fas | fasudil |
|  | Bel | belumosudil |
| Reagents and solutions | PFA | paraformaldehyde |
|  | abs | antibodies |
|  | α-SMA | alpha smooth muscle actin |
|  | CCL19 | chemokine (C-C motif) ligand 19 |
| Retinal layers | ONL | outer nuclear layer |
|  | INL | inner nuclear layer |
|  | GCL | ganglion cell layer |
|  | IPL | inner plexiform layer |
|  | OPL | outer plexiform layer |
|  | IS | inner segment of photoreceptors |
|  | OS | outer segment of the photoreceptors |
| Instruments and methods | AF | fundus autofluorescence |
|  | FA | fluorescein angiography |
|  | OCT | optical coherence tomography |
|  | LI-COR | odyssey infrared imaging system |
|  | H&E | hematoxylin and eosin |
|  | ICP | inductively coupled plasma |
|  | TOF | time of flight |
|  | IMC | imaging mass cytometry |
|  | IHC | Immunohistochemistry |
| Miscellaneous | IVC | individually ventilated cages |
|  | i.p. | intraperitoneally |
|  | ROI | regions of interest |
|  | IOP | intraocular pressure |
|  | CTCF | corrected total cell fluorescence |

| **Table S2. Hyperion Imaging System protein markers** | | | |
| --- | --- | --- | --- |
| **channel** | **name** | **keep** | **ilastik** |
| ArAr80 | 1 | 1 | 1 |
| I127 | 2 | 1 | 2 |
| Xe131 | 3 | 1 | 3 |
| Xe134 | 4 | 1 | 4 |
| Ba138 | 5 | 1 | 5 |
| La139 | 6 | 1 | 6 |
| Ce140 | 7 | 1 | 7 |
| Pr141 | α-SMA | 1 | 8 |
| Nd142 | 8 | 1 | 9 |
| Nd143 | 9 | 1 | 10 |
| Nd144 | pTyrosine | 1 | 11 |
| Nd145 | 10 | 1 | 12 |
| Nd146 | 11 | 1 | 13 |
| Sm147 | 12 | 1 | 14 |
| Sm148 | 13 | 1 | 15 |
| Sm149 | Vimentin | 1 | 16 |
| Nd150 | Ki67 | 1 | 17 |
| Eu151 | CD45 | 1 | 18 |
| Sm152 | Fibronectin | 1 | 19 |
| Eu153 | CD44 | 1 | 20 |
| Sm154 | beta_actin | 1 | 21 |
| Gd155 | GranzymeB | 1 | 22 |
| Gd156 | F480 | 1 | 23 |
| Gd157 | 14 | 1 | 24 |
| Gd158 | Ecadherin | 1 | 25 |
| Tb159 | CD4 | 1 | 26 |
| Gd160 | iNOS_CD206 | 1 | 27 |
| Dy161 | MHCII | 1 | 28 |
| Dy162 | CD8 | 1 | 29 |
| Dy163 | CD11b | 1 | 30 |
| Dy164 | Arginase_perk | 1 | 31 |
| Ho165 | Foxp3 | 1 | 32 |
| Er166 | Ly6G | 1 | 33 |
| Er167 | 15 | 1 | 34 |
| Er168 | 16 | 1 | 35 |
| Tm169 | Beta_catenin | 1 | 36 |
| Er170 | CD3 | 1 | 37 |
| Yb171 | CD31 | 1 | 38 |
| Yb172 | 17 | 1 | 39 |
| Yb173 | CollagenT1 | 1 | 40 |
| Yb174 | Pan_cytokeratin | 1 | 41 |
| Lu175 | 18 | 1 | 42 |
| Yb176 | B220 | 1 | 43 |
| BCKG190 | 19 | 1 | 44 |
| Ir191 | DNA1 | 1 | 45 |
| Ir193 | DNA2 | 1 | 46 |
| Pb208 | 20 | 1 | 47 |

| **Table S3. Hyperion cells anntotated methods** | | |
| --- | --- | --- |
| **Initial annotation** | **Renamed into** | **How it was anntoated** |
| Choriod- RPE | Choroid-RPE complex | Pan-cytokeratin positive, positive for most channels; also classified manually in Qupath based on location |
| CD44−positive | Retinal progenitor cells | CD44+ cells (clustering-based annotation) |
| ONL | Photoreceptors | Separated manually in Qupath |
| Vimentin−pos | Macroglia | Vimentin+ cells (clustering-based annotation) |
| INL | Interneurons | Classified manually in Qupath based on location |
| GCL | Ganglion cells | Classified manually in Qupath based on location |
| undefined | Unspecific cell types | Classified manually in Qupath based on location |
| Microglia_CD11b | Microglia | CD45- CD11b+ cells (shiny app annotation) |
| Macrophage_CD45CD11b | Macrophages | CD45+ CD11b+ cells (shiny app annotation) |
| Perivascular_αSMA | Perivascular cells | αSMA++ cells (shiny app annotation) |
| fibrosis | Choroid-RPE complex | Type 1 collagen + and fibronectin+ cells |

| **Table S4.1 Statistical analysis (Bonferroni's multiple comparisons test and two-way ANOVA)** | | | | | | | | | | | |
| --- | --- | --- | --- | --- | --- | --- | --- | --- | --- | --- | --- |
| **Image NO.** | | **Group** | **Bonferroni's multiple comparisons test** | | | | **n** | **Method** | **ANOVA table** | **F** | **p** |
|  |  |  | **Mean Diff.** | **95.00% CI of diff.** | **Summary** | **Adjusted**  **P Value** |  |  |  |  |  |
| Figure 2 | C | Dapi |  |  |  |  | 12 | two-way ANOVA | staining | F (1.052, 11.57) = 187.4 | P<0.0001 |
|  |  | Vehicle vs. Fasudil | 24213 | -202156 to 250582 | ns | >0.9999 |  |  | treatment | F (1.457, 16.03) = 0.1307 | P=0.8134 |
|  |  | Vehicle vs. Belumosudil | 26803 | -260388 to 313995 | ns | >0.9999 |  |  | staining x treatment | F (1.323, 14.55) = 0.2214 | P=0.7121 |
|  |  | Fasudil vs. Belumosudil | 2590 | -164132 to 169313 | ns | >0.9999 |  |  |  |  |  |
|  |  |  |  |  |  |  |  |  |  |  |  |
|  |  | ROCK1 |  |  |  |  |  |  |  |  |  |
|  |  | Vehicle vs. Fasudil | 474642 | 46792 to 902492 | * | 0.0288 |  |  |  |  |  |
|  |  | Vehicle vs. Belumosudil | 462623 | 44760 to 880486 | * | 0.0291 |  |  |  |  |  |
|  |  | Fasudil vs. Belumosudil | -12019 | -61271 to 37234 | ns | >0.9999 |  |  |  |  |  |
|  |  |  |  |  |  |  |  |  |  |  |  |
|  |  | Phalloidin |  |  |  |  |  |  |  |  |  |
|  |  | Vehicle vs. Fasudil | -85669 | -1678474 to 1507136 | ns | >0.9999 |  |  |  |  |  |
|  |  | Vehicle vs. Belumosudil | -58193 | -3218036 to 3101649 | ns | >0.9999 |  |  |  |  |  |
|  |  | Fasudil vs. Belumosudil | 27475 | -2456109 to 2511060 | ns | >0.9999 |  |  |  |  |  |
|  | G | ROCK2 |  |  |  |  | 12 | two-way ANOVA | staining x treatment | F (4, 66) = 0.2411 | P=0.9141 |
|  |  | Vehicle vs. Fasudil | 946258 | 287753 to 1604764 | ** | 0.0057 |  |  | staining | F (2, 33) = 21.49 | P<0.0001 |
|  |  | Vehicle vs. Belumosudil | 958659 | 312087 to 1605231 | ** | 0.0046 |  |  | treatment | F (1.664, 54.92) = 0.1228 | P=0.8483 |
|  |  | Fasudil vs. Belumosudil | 12401 | -15374 to 40176 | ns | 0.7022 |  |  | Subject | F (33, 66) = 2.951 | P<0.0001 |
| Figure 3 | C2 | 35d |  |  |  |  | 8 | two-way ANOVA | Time x treatment | F (2, 15) = 3.364 | P=0.0621 |
|  |  | Vehicle vs. Fasudil | 0 | -21897573 to 21897573 | ns | >0.9999 |  |  | Time | F (1, 15) = 17.49 | P=0.0008 |
|  |  | Vehicle vs. Belumosudil | 0 | -21897573 to 21897573 | ns | >0.9999 |  |  | treatment | F (2, 15) = 2.494 | P=0.1162 |
|  |  |  |  |  |  |  |  |  | Subject | F (15, 15) = 1.349 | P=0.2846 |
|  |  | 49d |  |  |  |  |  |  | \|  \| \| --- \| |  |  |
|  |  | Vehicle vs. Fasudil | 9935600 | -11961973 to 31833173 | ns | 0.5858 |  |  |  |  |  |
|  |  | Vehicle vs. Belumosudil | 3.1E+07 | 8877274 to 52672420 | ** | 0.0048 |  |  |  |  |  |
|  | D2 | 35d |  |  |  |  | 8 | two-way ANOVA | Time x treatment | F (2, 21) = 0.1411 | P=0.8692 |
|  |  | Vehicle vs. Fasudil | 1254 | -19275 to 21782 | ns | 0.9873 |  |  | Time | F (1, 21) = 1.110 | P=0.3040 |
|  |  | Vehicle vs. Belumosudil | -285.8 | -20814 to 20243 | ns | 0.9993 |  |  | treatment | F (2, 21) = 0.3520 | P=0.7073 |
|  |  | Fasudil vs. Belumosudil | -1540 | -22068 to 18989 | ns | 0.9809 |  |  | Subject | F (21, 21) = 0.5342 | P=0.9205 |
|  |  |  |  |  |  |  |  |  |  |  |  |
|  |  | 49d |  |  |  |  |  |  |  |  |  |
|  |  | Vehicle vs. Fasudil | 6446 | -14083 to 26974 | ns | 0.7154 |  |  |  |  |  |
|  |  | Vehicle vs. Belumosudil | 6455 | -14073 to 26984 | ns | 0.7147 |  |  |  |  |  |
|  |  | Fasudil vs. Belumosudil | 9.75 | -20519 to 20538 | ns | >0.9999 |  |  |  |  |  |
|  | F2 | 35d |  |  |  |  | 8 | two-way ANOVA | Time x treatment | F (2, 21) = 3.816 | P=0.0386 |
|  |  | Vehicle vs. Fasudil | -298.2 | -20181 to 19585 | ns | >0.9999 |  |  | Time | F (1, 21) = 40.30 | P<0.0001 |
|  |  | Vehicle vs. Belumosudil | 563.8 | -19319 to 20447 | ns | >0.9999 |  |  | treatment | F (2, 21) = 3.676 | P=0.0428 |
|  |  | Fasudil vs. Belumosudil | 862 | -19021 to 20745 | ns | >0.9999 |  |  | \| Subject \| \| --- \| | F (21, 21) = 1.072 | P=0.4377 |
|  |  |  |  |  |  |  |  |  |  |  |  |
|  |  | 49d |  |  |  |  |  |  |  |  |  |
|  |  | Vehicle vs. Fasudil | 24880 | 4997 to 44763 | ** | 0.0098 |  |  |  |  |  |
|  |  | Vehicle vs. Belumosudil | 28225 | 8342 to 48107 | ** | 0.003 |  |  |  |  |  |
|  |  | Fasudil vs. Belumosudil | 3345 | -16538 to 23227 | ns | >0.9999 |  |  |  |  |  |
| Figure 4 | B | Type 1 collagen |  |  |  |  | 3 | two-way ANOVA | Time x staining | F (8, 60) = 60.05 | P<0.0001 |
|  |  | 7 vs. 21 | -4.649 | -7.974 to -1.325 | * | 0.0114 |  |  | Time | F (2.663, 39.94) = 0.000 | P>0.9999 |
|  |  | 7 vs. 35 | -12.53 | -16.05 to -9.015 | *** | 0.0001 |  |  | staining | F (2, 15) = 3681 | P<0.0001 |
|  |  | 7 vs. 49 | -7.03 | -10.89 to -3.174 | ** | 0.0033 |  |  | Subject | F (15, 60) = 2.549 | P=0.0053 |
|  |  | 7 vs. 49 (Fasudil) | -0.2543 | -3.441 to 2.932 | ns | >0.9999 |  |  |  |  |  |
|  |  | 21 vs. 35 | -7.884 | -10.46 to -5.309 | *** | 0.0003 |  |  |  |  |  |
|  |  | 21 vs. 49 | -2.381 | -4.342 to -0.4204 | * | 0.0215 |  |  |  |  |  |
|  |  | 21 vs. 49 (Fasudil) | 4.395 | 2.732 to 6.058 | *** | 0.0006 |  |  |  |  |  |
|  |  | 35 vs. 49 | 5.503 | 3.787 to 7.219 | *** | 0.0002 |  |  |  |  |  |
|  |  | 35 vs. 49 (Fasudil) | 12.28 | 9.214 to 15.34 | **** | <0.0001 |  |  |  |  |  |
|  |  | 49 vs. 49 (Fasudil) | 6.776 | 4.230 to 9.322 | *** | 0.0005 |  |  |  |  |  |
|  |  |  |  |  |  |  |  |  |  |  |  |
|  |  | Dapi |  |  |  |  |  |  |  |  |  |
|  |  | 7 vs. 21 | -0.7353 | -5.502 to 4.031 | ns | >0.9999 |  |  |  |  |  |
|  |  | 7 vs. 35 | 0.1865 | -4.631 to 5.004 | ns | >0.9999 |  |  |  |  |  |
|  |  | 7 vs. 49 | 0.07117 | -4.141 to 4.283 | ns | >0.9999 |  |  |  |  |  |
|  |  | 7 vs. 49 (Fasudil) | 0.6485 | -4.731 to 6.028 | ns | >0.9999 |  |  |  |  |  |
|  |  | 21 vs. 35 | 0.9218 | -3.755 to 5.599 | ns | >0.9999 |  |  |  |  |  |
|  |  | 21 vs. 49 | 0.8065 | -1.688 to 3.301 | ns | >0.9999 |  |  |  |  |  |
|  |  | 21 vs. 49 (Fasudil) | 1.384 | -0.4784 to 3.246 | ns | 0.1644 |  |  |  |  |  |
|  |  | 35 vs. 49 | -0.1153 | -4.622 to 4.392 | ns | >0.9999 |  |  |  |  |  |
|  |  | 35 vs. 49 (Fasudil) | 0.462 | -4.000 to 4.924 | ns | >0.9999 |  |  |  |  |  |
|  |  | 49 vs. 49 (Fasudil) | 0.5773 | -2.895 to 4.050 | ns | >0.9999 |  |  |  |  |  |
|  |  |  |  |  |  |  |  |  |  |  |  |
|  |  | Others |  |  |  |  |  |  |  |  |  |
|  |  | 7 vs. 21 | 5.384 | -0.5538 to 11.32 | ns | 0.0751 |  |  |  |  |  |
|  |  | 7 vs. 35 | 12.35 | 5.396 to 19.30 | ** | 0.0037 |  |  |  |  |  |
|  |  | 7 vs. 49 | 6.959 | 0.1196 to 13.80 | * | 0.0465 |  |  |  |  |  |
|  |  | 7 vs. 49 (Fasudil) | -0.3942 | -8.077 to 7.288 | ns | >0.9999 |  |  |  |  |  |
|  |  | 21 vs. 35 | 6.962 | -0.1215 to 14.05 | ns | 0.0538 |  |  |  |  |  |
|  |  | 21 vs. 49 | 1.575 | -2.097 to 5.246 | ns | 0.9598 |  |  |  |  |  |
|  |  | 21 vs. 49 (Fasudil) | -5.779 | -8.321 to -3.237 | ** | 0.0012 |  |  |  |  |  |
|  |  | 35 vs. 49 | -5.388 | -11.11 to 0.3330 | ns | 0.0643 |  |  |  |  |  |
|  |  | 35 vs. 49 (Fasudil) | -12.74 | -20.17 to -5.307 | ** | 0.0044 |  |  |  |  |  |
|  |  | 49 vs. 49 (Fasudil) | -7.353 | -12.42 to -2.287 | ** | 0.0096 |  |  |  |  |  |
|  | C | Type 1 collagen |  |  |  |  | 3 | two-way ANOVA | Time x staining | F (8, 60) = 55.26 | P<0.0001 |
|  |  | 7 vs. 21 | -4.649 | -7.974 to -1.325 | * | 0.0114 |  |  | Time | F (2.665, 39.98) = 0.000 | P>0.9999 |
|  |  | 7 vs. 35 | -12.53 | -16.05 to -9.015 | *** | 0.0001 |  |  | staining | F (2, 15) = 5264 | P<0.0001 |
|  |  | 7 vs. 49 | -7.03 | -10.89 to -3.174 | ** | 0.0033 |  |  | \| Subject \| \| --- \| | F (15, 60) = 1.669 | P=0.0826 |
|  |  | 7 vs. 49 (Belumodudil) | -0.6585 | -4.450 to 3.133 | ns | >0.9999 |  |  |  |  |  |
|  |  | 21 vs. 35 | -7.884 | -10.46 to -5.309 | *** | 0.0003 |  |  |  |  |  |
|  |  | 21 vs. 49 | -2.381 | -4.342 to -0.4204 | * | 0.0215 |  |  |  |  |  |
|  |  | 21 vs. 49 (Belumodudil) | 3.991 | 1.030 to 6.951 | * | 0.0135 |  |  |  |  |  |
|  |  | 35 vs. 49 | 5.503 | 3.787 to 7.219 | *** | 0.0002 |  |  |  |  |  |
|  |  | 35 vs. 49 (Belumodudil) | 11.87 | 7.760 to 15.99 | *** | 0.0004 |  |  |  |  |  |
|  |  | 49 vs. 49 (Belumodudil) | 6.372 | 3.055 to 9.689 | ** | 0.0026 |  |  |  |  |  |
|  |  |  |  |  |  |  |  |  |  |  |  |
|  |  | Dapi |  |  |  |  |  |  |  |  |  |
|  |  | 7 vs. 21 | -0.7353 | -5.502 to 4.031 | ns | >0.9999 |  |  |  |  |  |
|  |  | 7 vs. 35 | 0.1865 | -4.631 to 5.004 | ns | >0.9999 |  |  |  |  |  |
|  |  | 7 vs. 49 | 0.07117 | -4.141 to 4.283 | ns | >0.9999 |  |  |  |  |  |
|  |  | 7 vs. 49 (Belumodudil) | 0.956 | -2.952 to 4.864 | ns | >0.9999 |  |  |  |  |  |
|  |  | 21 vs. 35 | 0.9218 | -3.755 to 5.599 | ns | >0.9999 |  |  |  |  |  |
|  |  | 21 vs. 49 | 0.8065 | -1.688 to 3.301 | ns | >0.9999 |  |  |  |  |  |
|  |  | 21 vs. 49 (Belumodudil) | 1.691 | -2.622 to 6.005 | ns | >0.9999 |  |  |  |  |  |
|  |  | 35 vs. 49 | -0.1153 | -4.622 to 4.392 | ns | >0.9999 |  |  |  |  |  |
|  |  | 35 vs. 49 (Belumodudil) | 0.7695 | -5.175 to 6.714 | ns | >0.9999 |  |  |  |  |  |
|  |  | 49 vs. 49 (Belumodudil) | 0.8848 | -2.275 to 4.044 | ns | >0.9999 |  |  |  |  |  |
|  |  |  |  |  |  |  |  |  |  |  |  |
|  |  | Others |  |  |  |  |  |  |  |  |  |
|  |  | 7 vs. 21 | 5.384 | -0.5538 to 11.32 | ns | 0.0751 |  |  |  |  |  |
|  |  | 7 vs. 35 | 12.35 | 5.396 to 19.30 | ** | 0.0037 |  |  |  |  |  |
|  |  | 7 vs. 49 | 6.959 | 0.1196 to 13.80 | * | 0.0465 |  |  |  |  |  |
|  |  | 7 vs. 49 (Belumodudil) | -0.2975 | -7.010 to 6.415 | ns | >0.9999 |  |  |  |  |  |
|  |  | 21 vs. 35 | 6.962 | -0.1215 to 14.05 | ns | 0.0538 |  |  |  |  |  |
|  |  | 21 vs. 49 | 1.575 | -2.097 to 5.246 | ns | 0.9598 |  |  |  |  |  |
|  |  | 21 vs. 49 (Belumodudil) | -5.682 | -9.653 to -1.711 | * | 0.0103 |  |  |  |  |  |
|  |  | 35 vs. 49 | -5.388 | -11.11 to 0.3330 | ns | 0.0643 |  |  |  |  |  |
|  |  | 35 vs. 49 (Belumodudil) | -12.64 | -21.26 to -4.030 | ** | 0.0091 |  |  |  |  |  |
|  |  | 49 vs. 49 (Belumodudil) | -7.257 | -11.25 to -3.268 | ** | 0.0033 |  |  |  |  |  |
|  | D | Type 1 collagen |  |  |  |  | 3 | two-way ANOVA | Time x staining | F (8, 60) = 51.43 | P<0.0001 |
|  |  | 7 vs. 21 | -3.416 | -7.569 to 0.7366 | ns | 0.1111 |  |  | Time | F (2.377, 35.66) = 0.000 | P>0.9999 |
|  |  | 7 vs. 35 | -11.69 | -14.67 to -8.706 | **** | <0.0001 |  |  | staining | F (2, 15) = 10754 | P<0.0001 |
|  |  | 7 vs. 49 | -5.884 | -9.959 to -1.809 | ** | 0.0098 |  |  | Subject | F (15, 60) = 0.7111 | P=0.7635 |
|  |  | 7 vs. 49 (Fasudil) | -0.2352 | -5.007 to 4.536 | ns | >0.9999 |  |  |  |  |  |
|  |  | 21 vs. 35 | -8.272 | -11.53 to -5.019 | *** | 0.0007 |  |  |  |  |  |
|  |  | 21 vs. 49 | -2.468 | -3.639 to -1.297 | ** | 0.0017 |  |  |  |  |  |
|  |  | 21 vs. 49 (Fasudil) | 3.181 | 1.863 to 4.499 | *** | 0.0009 |  |  |  |  |  |
|  |  | 35 vs. 49 | 5.804 | 3.296 to 8.312 | ** | 0.0011 |  |  |  |  |  |
|  |  | 35 vs. 49 (Fasudil) | 11.45 | 7.899 to 15.01 | *** | 0.0002 |  |  |  |  |  |
|  |  | 49 vs. 49 (Fasudil) | 5.649 | 4.082 to 7.216 | *** | 0.0001 |  |  |  |  |  |
|  |  |  |  |  |  |  |  |  |  |  |  |
|  |  | Dapi |  |  |  |  |  |  |  |  |  |
|  |  | 7 vs. 21 | -0.09417 | -4.967 to 4.779 | ns | >0.9999 |  |  |  |  |  |
|  |  | 7 vs. 35 | -1.281 | -4.687 to 2.125 | ns | >0.9999 |  |  |  |  |  |
|  |  | 7 vs. 49 | 0.4257 | -4.739 to 5.591 | ns | >0.9999 |  |  |  |  |  |
|  |  | 7 vs. 49 (Fasudil) | 0.4602 | -3.853 to 4.774 | ns | >0.9999 |  |  |  |  |  |
|  |  | 21 vs. 35 | -1.187 | -5.990 to 3.616 | ns | >0.9999 |  |  |  |  |  |
|  |  | 21 vs. 49 | 0.5198 | -3.639 to 4.679 | ns | >0.9999 |  |  |  |  |  |
|  |  | 21 vs. 49 (Fasudil) | 0.5543 | -2.490 to 3.598 | ns | >0.9999 |  |  |  |  |  |
|  |  | 35 vs. 49 | 1.707 | -2.354 to 5.768 | ns | >0.9999 |  |  |  |  |  |
|  |  | 35 vs. 49 (Fasudil) | 1.741 | -1.974 to 5.457 | ns | 0.7549 |  |  |  |  |  |
|  |  | 49 vs. 49 (Fasudil) | 0.0345 | -5.415 to 5.484 | ns | >0.9999 |  |  |  |  |  |
|  |  |  |  |  |  |  |  |  |  |  |  |
|  |  | Others |  |  |  |  |  |  |  |  |  |
|  |  | 7 vs. 21 | 3.51 | -4.747 to 11.77 | ns | 0.982 |  |  |  |  |  |
|  |  | 7 vs. 35 | 12.97 | 7.948 to 17.99 | *** | 0.0006 |  |  |  |  |  |
|  |  | 7 vs. 49 | 5.459 | -1.152 to 12.07 | ns | 0.1094 |  |  |  |  |  |
|  |  | 7 vs. 49 (Fasudil) | -0.225 | -8.372 to 7.922 | ns | >0.9999 |  |  |  |  |  |
|  |  | 21 vs. 35 | 9.459 | 1.684 to 17.23 | * | 0.0214 |  |  |  |  |  |
|  |  | 21 vs. 49 | 1.948 | -2.747 to 6.643 | ns | >0.9999 |  |  |  |  |  |
|  |  | 21 vs. 49 (Fasudil) | -3.736 | -6.882 to -0.5889 | * | 0.0238 |  |  |  |  |  |
|  |  | 35 vs. 49 | -7.511 | -13.11 to -1.916 | * | 0.0137 |  |  |  |  |  |
|  |  | 35 vs. 49 (Fasudil) | -13.19 | -20.01 to -6.376 | ** | 0.0025 |  |  |  |  |  |
|  |  | 49 vs. 49 (Fasudil) | -5.684 | -11.72 to 0.3503 | ns | 0.0642 |  |  |  |  |  |
|  | E | α-SMA |  |  |  |  | 3 | two-way ANOVA | Time x staining | F (8, 60) = 55.37 | P<0.0001 |
|  |  | 7 vs. 21 | -3.416 | -7.569 to 0.7366 | ns | 0.1111 |  |  | Time | F (2.347, 35.20) = 0.000 | P>0.9999 |
|  |  | 7 vs. 35 | -11.69 | -14.67 to -8.706 | **** | <0.0001 |  |  | staining | F (2, 15) = 17539 | P<0.0001 |
|  |  | 7 vs. 49 | -5.884 | -9.959 to -1.809 | ** | 0.0098 |  |  | \| Subject \| \| --- \| | F (15, 60) = 0.4742 | P=0.9447 |
|  |  | 7 vs. 49 (Belumosudil) | -0.309 | -4.903 to 4.285 | ns | >0.9999 |  |  |  |  |  |
|  |  | 21 vs. 35 | -8.272 | -11.53 to -5.019 | *** | 0.0007 |  |  |  |  |  |
|  |  | 21 vs. 49 | -2.468 | -3.639 to -1.297 | ** | 0.0017 |  |  |  |  |  |
|  |  | 21 vs. 49 (Belumosudil) | 3.107 | 1.913 to 4.302 | *** | 0.0006 |  |  |  |  |  |
|  |  | 35 vs. 49 | 5.804 | 3.296 to 8.312 | ** | 0.0011 |  |  |  |  |  |
|  |  | 35 vs. 49 (Belumosudil) | 11.38 | 7.240 to 15.52 | *** | 0.0005 |  |  |  |  |  |
|  |  | 49 vs. 49 (Belumosudil) | 5.575 | 3.354 to 7.797 | *** | 0.0007 |  |  |  |  |  |
|  |  |  |  |  |  |  |  |  |  |  |  |
|  |  | Dapi |  |  |  |  |  |  |  |  |  |
|  |  | 7 vs. 21 | -0.09417 | -4.967 to 4.779 | ns | >0.9999 |  |  |  |  |  |
|  |  | 7 vs. 35 | -1.281 | -4.687 to 2.125 | ns | >0.9999 |  |  |  |  |  |
|  |  | 7 vs. 49 | 0.4257 | -4.739 to 5.591 | ns | >0.9999 |  |  |  |  |  |
|  |  | 7 vs. 49 (Belumosudil) | 0.32 | -4.374 to 5.014 | ns | >0.9999 |  |  |  |  |  |
|  |  | 21 vs. 35 | -1.187 | -5.990 to 3.616 | ns | >0.9999 |  |  |  |  |  |
|  |  | 21 vs. 49 | 0.5198 | -3.639 to 4.679 | ns | >0.9999 |  |  |  |  |  |
|  |  | 21 vs. 49 (Belumosudil) | 0.4142 | -4.287 to 5.115 | ns | >0.9999 |  |  |  |  |  |
|  |  | 35 vs. 49 | 1.707 | -2.354 to 5.768 | ns | >0.9999 |  |  |  |  |  |
|  |  | 35 vs. 49 (Belumosudil) | 1.601 | -2.658 to 5.861 | ns | >0.9999 |  |  |  |  |  |
|  |  | 49 vs. 49 (Belumosudil) | -0.1057 | -1.522 to 1.311 | ns | >0.9999 |  |  |  |  |  |
|  |  |  |  |  |  |  |  |  |  |  |  |
|  |  | Others |  |  |  |  |  |  |  |  |  |
|  |  | 7 vs. 21 | 3.51 | -4.747 to 11.77 | ns | 0.982 |  |  |  |  |  |
|  |  | 7 vs. 35 | 12.97 | 7.948 to 17.99 | *** | 0.0006 |  |  |  |  |  |
|  |  | 7 vs. 49 | 5.459 | -1.152 to 12.07 | ns | 0.1094 |  |  |  |  |  |
|  |  | 7 vs. 49 (Belumosudil) | -0.011 | -6.106 to 6.084 | ns | >0.9999 |  |  |  |  |  |
|  |  | 21 vs. 35 | 9.459 | 1.684 to 17.23 | * | 0.0214 |  |  |  |  |  |
|  |  | 21 vs. 49 | 1.948 | -2.747 to 6.643 | ns | >0.9999 |  |  |  |  |  |
|  |  | 21 vs. 49 (Belumosudil) | -3.522 | -8.020 to 0.9771 | ns | 0.1348 |  |  |  |  |  |
|  |  | 35 vs. 49 | -7.511 | -13.11 to -1.916 | * | 0.0137 |  |  |  |  |  |
|  |  | 35 vs. 49 (Belumosudil) | -12.98 | -19.46 to -6.497 | ** | 0.0021 |  |  |  |  |  |
|  |  | 49 vs. 49 (Belumosudil) | -5.47 | -7.423 to -3.516 | *** | 0.0004 |  |  |  |  |  |
| Figure 5 | B | Type 1 collagen |  |  |  |  | 3 | two-way ANOVA | Time x staining | F (9, 60) = 114.6 | P<0.0001 |
|  |  | 7 vs. 35 | -12.53 | -15.64 to -9.424 | **** | <0.0001 |  |  | Time | F (2.385, 47.70) = 0.000 | P>0.9999 |
|  |  | 7 vs. 49 | -7.03 | -10.44 to -3.622 | ** | 0.002 |  |  | staining | F (3, 20) = 2514 | P<0.0001 |
|  |  | 7 vs. 49 (Fasudil) | -0.2543 | -3.071 to 2.562 | ns | >0.9999 |  |  | Subject | F (20, 60) = 1.196 | P=0.2899 |
|  |  | 35 vs. 49 | 5.503 | 3.986 to 7.020 | *** | 0.0001 |  |  | \|  \| \| --- \| |  |  |
|  |  | 35 vs. 49 (Fasudil) | 12.28 | 9.570 to 14.99 | **** | <0.0001 |  |  |  |  |  |
|  |  | 49 vs. 49 (Fasudil) | 6.776 | 4.526 to 9.026 | *** | 0.0003 |  |  |  |  |  |
|  |  |  |  |  |  |  |  |  |  |  |  |
|  |  | Dapi |  |  |  |  |  |  |  |  |  |
|  |  | 7 vs. 35 | 0.1865 | -4.072 to 4.445 | ns | >0.9999 |  |  |  |  |  |
|  |  | 7 vs. 49 | 0.07117 | -3.652 to 3.795 | ns | >0.9999 |  |  |  |  |  |
|  |  | 7 vs. 49 (Fasudil) | 0.6485 | -4.107 to 5.404 | ns | >0.9999 |  |  |  |  |  |
|  |  | 35 vs. 49 | -0.1153 | -4.099 to 3.869 | ns | >0.9999 |  |  |  |  |  |
|  |  | 35 vs. 49 (Fasudil) | 0.462 | -3.482 to 4.406 | ns | >0.9999 |  |  |  |  |  |
|  |  | 49 vs. 49 (Fasudil) | 0.5773 | -2.492 to 3.647 | ns | >0.9999 |  |  |  |  |  |
|  |  |  |  |  |  |  |  |  |  |  |  |
|  |  | α-sma |  |  |  |  |  |  |  |  |  |
|  |  | 7 vs. 35 | -11.69 | -14.32 to -9.052 | **** | <0.0001 |  |  |  |  |  |
|  |  | 7 vs. 49 | -5.884 | -9.486 to -2.282 | ** | 0.0059 |  |  |  |  |  |
|  |  | 7 vs. 49 (Fasudil) | -0.2352 | -4.453 to 3.983 | ns | >0.9999 |  |  |  |  |  |
|  |  | 35 vs. 49 | 5.804 | 3.587 to 8.021 | *** | 0.0006 |  |  |  |  |  |
|  |  | 35 vs. 49 (Fasudil) | 11.45 | 8.312 to 14.59 | *** | 0.0001 |  |  |  |  |  |
|  |  | 49 vs. 49 (Fasudil) | 5.649 | 4.263 to 7.035 | **** | <0.0001 |  |  |  |  |  |
|  |  |  |  |  |  |  |  |  |  |  |  |
|  |  | Others |  |  |  |  |  |  |  |  |  |
|  |  | 7 vs. 35 | 24.04 | 16.58 to 31.49 | *** | 0.0002 |  |  |  |  |  |
|  |  | 7 vs. 49 | 12.84 | 6.770 to 18.92 | ** | 0.0018 |  |  |  |  |  |
|  |  | 7 vs. 49 (Fasudil) | -0.159 | -8.817 to 8.499 | ns | >0.9999 |  |  |  |  |  |
|  |  | 35 vs. 49 | -11.19 | -16.96 to -5.424 | ** | 0.0027 |  |  |  |  |  |
|  |  | 35 vs. 49 (Fasudil) | -24.19 | -33.07 to -15.32 | *** | 0.0005 |  |  |  |  |  |
|  |  | 49 vs. 49 (Fasudil) | -13 | -18.40 to -7.606 | *** | 0.0009 |  |  |  |  |  |
|  | C | Type 1 collagen |  |  |  |  | 3 | two-way ANOVA | Time x staining | F (9, 60) = 113.3 | P<0.0001 |
|  |  | 7 vs. 35 | -12.53 | -15.64 to -9.424 | **** | <0.0001 |  |  | Time | F (2.019, 40.38) = 0.000 | P>0.9999 |
|  |  | 7 vs. 49 | -7.03 | -10.44 to -3.622 | ** | 0.002 |  |  | staining | F (3, 20) = 3444 | P<0.0001 |
|  |  | 7 vs. 49 (Belumosudil) | -0.6585 | -4.010 to 2.693 | ns | >0.9999 |  |  | \| Subject \| \| --- \| | F (20, 60) = 0.8693 | P=0.6237 |
|  |  | 35 vs. 49 | 5.503 | 3.986 to 7.020 | *** | 0.0001 |  |  |  |  |  |
|  |  | 35 vs. 49 (Belumosudil) | 11.87 | 8.238 to 15.51 | *** | 0.0002 |  |  |  |  |  |
|  |  | 49 vs. 49 (Belumosudil) | 6.372 | 3.440 to 9.304 | ** | 0.0016 |  |  |  |  |  |
|  |  |  |  |  |  |  |  |  |  |  |  |
|  |  | Dapi |  |  |  |  |  |  |  |  |  |
|  |  | 7 vs. 35 | 0.1865 | -4.072 to 4.445 | ns | >0.9999 |  |  |  |  |  |
|  |  | 7 vs. 49 | 0.07117 | -3.652 to 3.795 | ns | >0.9999 |  |  |  |  |  |
|  |  | 7 vs. 49 (Belumosudil) | 0.956 | -2.498 to 4.410 | ns | >0.9999 |  |  |  |  |  |
|  |  | 35 vs. 49 | -0.1153 | -4.099 to 3.869 | ns | >0.9999 |  |  |  |  |  |
|  |  | 35 vs. 49 (Belumosudil) | 0.7695 | -4.485 to 6.024 | ns | >0.9999 |  |  |  |  |  |
|  |  | 49 vs. 49 (Belumosudil) | 0.8848 | -1.908 to 3.677 | ns | >0.9999 |  |  |  |  |  |
|  |  |  |  |  |  |  |  |  |  |  |  |
|  |  | α-sma |  |  |  |  |  |  |  |  |  |
|  |  | 7 vs. 35 | -11.69 | -14.32 to -9.052 | **** | <0.0001 |  |  |  |  |  |
|  |  | 7 vs. 49 | -5.884 | -9.486 to -2.282 | ** | 0.0059 |  |  |  |  |  |
|  |  | 7 vs. 49 (Belumosudil) | -0.309 | -4.370 to 3.752 | ns | >0.9999 |  |  |  |  |  |
|  |  | 35 vs. 49 | 5.804 | 3.587 to 8.021 | *** | 0.0006 |  |  |  |  |  |
|  |  | 35 vs. 49 (Belumosudil) | 11.38 | 7.721 to 15.04 | *** | 0.0003 |  |  |  |  |  |
|  |  | 49 vs. 49 (Belumosudil) | 5.575 | 3.611 to 7.539 | *** | 0.0004 |  |  |  |  |  |
|  |  |  |  |  |  |  |  |  |  |  |  |
|  |  | Others |  |  |  |  |  |  |  |  |  |
|  |  | 7 vs. 35 | 24.04 | 16.58 to 31.49 | *** | 0.0002 |  |  |  |  |  |
|  |  | 7 vs. 49 | 12.84 | 6.770 to 18.92 | ** | 0.0018 |  |  |  |  |  |
|  |  | 7 vs. 49 (Belumosudil) | 0.0115 | -7.019 to 7.042 | ns | >0.9999 |  |  |  |  |  |
|  |  | 35 vs. 49 | -11.19 | -16.96 to -5.424 | ** | 0.0027 |  |  |  |  |  |
|  |  | 35 vs. 49 (Belumosudil) | -24.02 | -33.89 to -14.16 | *** | 0.0009 |  |  |  |  |  |
|  |  | 49 vs. 49 (Belumosudil) | -12.83 | -17.20 to -8.467 | *** | 0.0004 |  |  |  |  |  |

| **Table S5. Comparison between groups (one-way ANOVA)** | | | | | |
| --- | --- | --- | --- | --- | --- |
| **Image NO.** | | **Subject** | **SD** | | |
|  |  |  | **F** | **df** | ***p*** |
| Figure 2 | D | Treatment (between columns) | F (1.020, 11.22) = 9.705 | 2 | *p* =0.0094 |
|  |  | Individual (between rows) | F (11, 22) = 0.8793 | 11 | *p* =0.5723 |
|  | H | Treatment (between columns) | F (1.014, 11.15) = 47.88 | 2 | *p* <0.0001 |
|  |  | Individual (between rows) | F (11, 22) = 1.073 | 11 | *p* =0.4240 |
|  | K | Treatment (between columns) | F (1.807, 14.46) = 134.7 | 2 | *p* <0.0001 |
|  |  | Individual (between rows) | F (8, 16) = 6.872 | 8 | *p* =0.0006 |
| Figure 3 | C1 | Treatment (between columns) | F (2.022, 10.11) = 5.304 | 3 | *p* =0.0264 |
|  |  | Individual (between rows) | F (5, 15) = 0.1753 | 5 | *p* =0.9678 |
|  | D1 | Treatment (between columns) | F (2.137, 49.15) = 4.127 | 3 | *p* =0.0199 |
|  |  | Individual (between rows) | F (23, 69) = 1.213 | 23 | *p* =0.2649 |
|  | F1 | Treatment (between columns) | F (2.605, 59.91) = 22.37 | 3 | *p* <0.0001 |
|  |  | Individual (between rows) | F (23, 69) = 0.6057 | 23 | *p* =0.9104 |

| **Table S6.1 SEM and P value of the t-test in Figure 6** | | | | |
| --- | --- | --- | --- | --- |
| **Image NO.** | **Subject** | **t-test** | | |
|  |  | **n** | **SEM** | ***p*** |
| A.a p-MYPT1 (c) | Vehicle | 3 | 0.0000 | \|  \| \| --- \| |
|  | d7 vs Vehicle | 3 | 0.2556 | 0.1580 |
|  | d35 vs d7 | 3 | 0.5045 | 0.2100 |
|  | d49 vs d7 | 3 | 0.4742 | 0.4277 |
|  | d49 (F) vs d49 | 3 | 0.2124 | 0.0162 |
|  | d49 (B) vs d49 | 3 | 0.1906 | 0.0143 |
| A.b Type 1 collagen (c) | Vehicle | 4 | 0.0000 | \|  \| \| --- \| |
|  | d7 vs Vehicle | 4 | 0.2494 | 0.2111 |
|  | d35 vs d7 | 4 | 0.2408 | 0.0185 |
|  | d49 vs d7 | 4 | 0.1148 | 0.0000 |
|  | d49 (F) vs d49 | 4 | 0.3609 | 0.0084 |
|  | d49 (B) vs d49 | 4 | 0.2743 | 0.0015 |
| A.c α-SMA (c) | Vehicle | 3 | 0.0000 | \|  \| \| --- \| |
|  | d7 vs Vehicle | 3 | 0.3703 | 0.3439 |
|  | d35 vs d7 | 3 | 0.1623 | 0.0918 |
|  | d49 vs d7 | 3 | 0.1802 | 0.1301 |
|  | d49 (F) vs d49 | 3 | 0.3030 | 0.0322 |
|  | d49 (B) vs d49 | 3 | 0.1884 | 0.0101 |
| A.d Vimentin (c) | Vehicle | 3 | 0.0000 |  |
|  | d7 vs Vehicle | 3 | 0.0826 | 0.0001 |
|  | d35 vs d7 | 3 | 0.3573 | 0.5040 |
|  | d49 vs d7 | 3 | 0.1918 | 0.5145 |
|  | d49 (F) vs d49 | 3 | 0.3640 | 0.0236 |
|  | d49 (B) vs d49 | 3 | 0.2027 | 0.0033 |
| A.e Fibronectin (c) | Vehicle | 3 | 0.0000 |  |
|  | d7 vs Vehicle | 3 | 0.2537 | 0.6024 |
|  | d35 vs d7 | 3 | 0.3528 | 0.3827 |
|  | d49 vs d7 | 3 | 0.0539 | 0.0485 |
|  | d49 (F) vs d49 | 3 | 0.2659 | 0.0075 |
|  | d49 (B) vs d49 | 3 | 0.3027 | 0.0047 |
| A.f TGF-β1 (c) | Vehicle | 3 | 0.0000 |  |
|  | d7 vs Vehicle | 3 | 0.4859 | 0.0286 |
|  | d35 vs d7 | 3 | 0.1249 | 0.1634 |
|  | d49 vs d7 | 3 | 0.4697 | 0.0017 |
|  | d49 (F) vs d49 | 3 | 0.1102 | 0.0006 |
|  | d49 (B) vs d49 | 3 | 0.0928 | 0.0006 |
| B.a p-MYPT1 (r) | Vehicle | 3 | 0.0000 |  |
|  | d7 vs Vehicle | 3 | 7.5541 | 0.3677 |
|  | d35 vs d7 | 3 | 0.1385 | 0.3750 |
|  | d49 vs d7 | 3 | 1.0996 | 0.3603 |
|  | d49 (F) vs d49 | 3 | 0.3093 | 0.5385 |
|  | d49 (B) vs d49 | 3 | 0.1952 | 0.2179 |
| B.b Type 1 collagen (r) | Vehicle | 3 | 0.0000 |  |
|  | d7 vs Vehicle | 3 | 0.6060 | 0.1445 |
|  | d35 vs d7 | 3 | 0.4988 | 0.1005 |
|  | d49 vs d7 | 3 | 0.2337 | 0.0308 |
|  | d49 (F) vs d49 | 3 | 0.1941 | 0.0432 |
|  | d49 (B) vs d49 | 3 | 0.1607 | 0.0494 |
| B.c α-SMA (r) | Vehicle | 3 | 0.0000 |  |
|  | d7 vs Vehicle | 3 | 0.8307 | 0.4269 |
|  | d35 vs d7 | 3 | 0.3261 | 0.7958 |
|  | d49 vs d7 | 3 | 0.8496 | 0.6008 |
|  | d49 (F) vs d49 | 3 | 0.0576 | 0.2518 |
|  | d49 (B) vs d49 | 3 | 0.3377 | 0.4068 |
| B.d Vimentin (r) | Vehicle | 3 | 0.0000 |  |
|  | d7 vs Vehicle | 3 | 0.4109 | 0.1608 |
|  | d35 vs d7 | 3 | 0.2671 | 0.6458 |
|  | d49 vs d7 | 3 | 0.5419 | 0.6325 |
|  | d49 (F) vs d49 | 3 | 0.1527 | 0.3876 |
|  | d49 (B) vs d49 | 3 | 0.1027 | 0.6603 |
| B.e Fibronectin (r) | Vehicle | 3 | 0.0000 |  |
|  | d7 vs Vehicle | 3 | 0.5431 | 0.0233 |
|  | d35 vs d7 | 3 | 0.7753 | 0.5093 |
|  | d49 vs d7 | 3 | 0.8167 | 0.0360 |
|  | d49 (F) vs d49 | 3 | 0.6487 | 0.0077 |
|  | d49 (B) vs d49 | 3 | 0.6300 | 0.0096 |
| B.f TGF-β1 (c) | Vehicle | 3 | 0.0000 |  |
|  | d7 vs Vehicle | 3 | 0.2158 | 0.3624 |
|  | d35 vs d7 | 3 | 0.3956 | 0.5842 |
|  | d49 vs d7 | 3 | 0.1253 | 0.7961 |
|  | d49 (F) vs d49 | 3 | 0.0148 | 0.0068 |
|  | d49 (B) vs d49 | 3 | 0.1123 | 0.0586 |

| **Table S7. DA results donor of Figure 7** | | | | | | |
| --- | --- | --- | --- | --- | --- | --- |
| **Groups** | **Cells annotation** | **logFC** | **logCPM** | **F** | ***p*** | **FDR** |
| LC vs C | Ganglion cells | -2.31736276 | 15.41494643 | 43.4052424 | 0.0001 | 0.0002 |
|  | Choroid-RPE complex | 1.42839252 | 17.66795909 | 42.68239272 | 0.0001 | 0.0002 |
|  | Macrophages | 7.869718598 | 12.58163799 | 78.01725065 | 0.0000 | 0.0002 |
|  | Interneurons | -1.080805218 | 17.46180942 | 22.06851583 | 0.0009 | 0.0022 |
|  | Photoreceptors | -0.932189292 | 18.50454774 | 19.44472836 | 0.0014 | 0.0027 |
|  | Perivascular cells | 2.953862554 | 12.87242085 | 9.962103002 | 0.0108 | 0.0181 |
|  | Unspecific cell types | 2.961695757 | 15.5265813 | 8.324530759 | 0.0170 | 0.0243 |
|  | Retinal progenitor cells | 2.080695472 | 15.89285415 | 7.49095461 | 0.0218 | 0.0273 |
|  | Macroglia | 1.513834073 | 16.16509223 | 5.425986076 | 0.0433 | 0.0481 |
|  | Microglia | -2.049233953 | 12.83926041 | 4.618100692 | 0.0585 | 0.0585 |
| LC vs Fas | Ganglion cells | -2.608907868 | 15.41494643 | 54.07567551 | 0.0000 | 0.0003 |
|  | Macroglia | 3.241675203 | 16.16509223 | 21.60294477 | 0.0010 | 0.0052 |
|  | Interneurons | -0.788817862 | 17.46180942 | 11.85961203 | 0.0064 | 0.0215 |
|  | Choroid-RPE complex | -0.69085468 | 17.66795909 | 10.35653337 | 0.0094 | 0.0235 |
|  | Macrophages | 1.050212028 | 12.58163799 | 4.589971979 | 0.0667 | 0.1333 |
|  | Retinal progenitor cells | 1.107768776 | 15.89285415 | 2.258776785 | 0.1653 | 0.2755 |
|  | Perivascular cells | 1.1300887 | 12.87242085 | 1.835964882 | 0.2067 | 0.2953 |
|  | Photoreceptors | -0.167231113 | 18.50454774 | 0.635177793 | 0.4443 | 0.5554 |
|  | Microglia | -0.585693805 | 12.83926041 | 0.353501171 | 0.5660 | 0.6289 |
|  | Unspecific cell types | 0.36042038 | 15.5265813 | 0.14720553 | 0.7096 | 0.7096 |
| LC vs Bel | Ganglion cells | -2.423147812 | 15.41494643 | 47.06433221 | 0.0000 | 0.0005 |
|  | Macroglia | 3.832118341 | 16.16509223 | 28.20453323 | 0.0004 | 0.0020 |
|  | Choroid-RPE complex | -0.875291836 | 17.66795909 | 16.53728331 | 0.0023 | 0.0067 |
|  | Macrophages | 2.324653697 | 12.58163799 | 19.24611097 | 0.0027 | 0.0067 |
|  | Interneurons | -0.869005964 | 17.46180942 | 14.35671242 | 0.0037 | 0.0073 |
|  | Retinal progenitor cells | 2.092341309 | 15.89285415 | 7.510491074 | 0.0217 | 0.0362 |
|  | Perivascular cells | 1.95145862 | 12.87242085 | 4.881948413 | 0.0529 | 0.0756 |
|  | Photoreceptors | -0.329555567 | 18.50454774 | 2.463078287 | 0.1481 | 0.1646 |
|  | Unspecific cell types | 1.58456104 | 15.5265813 | 2.671740017 | 0.1347 | 0.1646 |
|  | Microglia | -0.288272924 | 12.83926041 | 0.08115982 | 0.7818 | 0.7818 |
| Fas vs Bel | Macrophages | 1.274441669 | 12.58163799 | 5.609065008 | 0.0473 | 0.4727 |
|  | Choroid-RPE complex | -0.184437156 | 17.66795909 | 0.746451521 | 0.4082 | 0.6531 |
|  | Photoreceptors | -0.162324454 | 18.50454774 | 0.598953 | 0.4572 | 0.6531 |
|  | Macroglia | 0.590443139 | 16.16509223 | 0.78353833 | 0.3979 | 0.6531 |
|  | Perivascular cells | 0.821369921 | 12.87242085 | 0.860293895 | 0.3766 | 0.6531 |
|  | Retinal progenitor cells | 0.984572534 | 15.89285415 | 1.746816275 | 0.2172 | 0.6531 |
|  | Unspecific cell types | 1.22414066 | 15.5265813 | 1.619466053 | 0.2334 | 0.6531 |
|  | Ganglion cells | 0.185760056 | 15.41494643 | 0.32277533 | 0.5827 | 0.7284 |
|  | Interneurons | -0.080188102 | 17.46180942 | 0.124528827 | 0.7316 | 0.7655 |
|  | Microglia | 0.29742088 | 12.83926041 | 0.094202676 | 0.7655 | 0.7655 |
| C vs Fas | Choroid-RPE complex | -2.119247201 | 17.66795909 | 90.33909477 | 0.0000 | 0.0000 |
|  | Macrophages | -6.81950657 | 12.58163799 | 53.54939187 | 0.0001 | 0.0006 |
|  | Photoreceptors | 0.764958179 | 18.50454774 | 13.17409556 | 0.0047 | 0.0158 |
|  | Unspecific cell types | -2.601275377 | 15.5265813 | 6.582809748 | 0.0291 | 0.0583 |
|  | Macroglia | 1.72784113 | 16.16509223 | 6.793385766 | 0.0272 | 0.0583 |
|  | Perivascular cells | -1.823773855 | 12.87242085 | 3.808516082 | 0.0810 | 0.1350 |
|  | Microglia | 1.463540149 | 12.83926041 | 2.583247173 | 0.1406 | 0.2009 |
|  | Retinal progenitor cells | -0.972926697 | 15.89285415 | 1.722181468 | 0.2202 | 0.2536 |
|  | Interneurons | 0.291987356 | 17.46180942 | 1.650941493 | 0.2283 | 0.2536 |
|  | Ganglion cells | -0.291545108 | 15.41494643 | 0.796511642 | 0.3935 | 0.3935 |
| C vs Bel | Choroid-RPE complex | -2.303684356 | 17.66795909 | 105.3875431 | 0.0000 | 0.0000 |
|  | Macrophages | -5.545064901 | 12.58163799 | 30.0716766 | 0.0007 | 0.0036 |
|  | Macroglia | 2.318284269 | 16.16509223 | 11.51410316 | 0.0073 | 0.0245 |
|  | Photoreceptors | 0.602633725 | 18.50454774 | 8.21229474 | 0.0171 | 0.0427 |
|  | Microglia | 1.760961029 | 12.83926041 | 3.532407493 | 0.0911 | 0.1822 |
|  | Unspecific cell types | -1.377134717 | 15.5265813 | 1.931709983 | 0.1962 | 0.3270 |
|  | Perivascular cells | -1.002403934 | 12.87242085 | 1.061265148 | 0.3284 | 0.4669 |
|  | Interneurons | 0.211799254 | 17.46180942 | 0.86928714 | 0.3735 | 0.4669 |
|  | Ganglion cells | -0.105785052 | 15.41494643 | 0.104620767 | 0.7531 | 0.8368 |
|  | Retinal progenitor cells | 0.011645837 | 15.89285415 | 0.000246307 | 0.9878 | 0.9878 |

| **Table S8. Totall cell numbers** | | | | | | | |
| --- | --- | --- | --- | --- | --- | --- | --- |
| **Cells** | **day1_002** | **day1_003** | **day1_004** | **day1_005** | **day1_006** | **day1_007** |  |
| Choroid-RPE complex | 149 | 146 | 65 | 60 | 108 | 372 |  |
| Perivascular cells | 10 | 9 | 0 | 0 | 2 | 5 |  |
| Macroglia | 194 | 166 | 104 | 96 | 29 | 10 |  |
| Microglia | 4 | 5 | 44 | 20 | 1 | 2 |  |
| Macrophages | 1 | 15 | 0 | 0 | 10 | 5 |  |
| Retinal progenitor cells | 65 | 172 | 5 | 14 | 58 | 29 |  |
| Photoreceptors | 366 | 276 | 584 | 610 | 351 | 316 |  |
| Interneurons | 140 | 59 | 255 | 269 | 169 | 182 |  |
| Ganglion cells | 7 | 9 |  | 89 | 57 | 35 |  |
| Unspecific cell types | 21 | 90 | 17 | 33 | 91 | 5 |  |
|  |  |  |  |  |  |  |  |
|  | day1_008 | day1_009 | slide2_001 | slide2_002 | slide2_003 | slide2_004 | slide2_005 |
| Choroid-RPE complex | 318 | 201 | 187 | 143 | 53 | 96 | 298 |
| Perivascular cells | 7 | 1 | 9 | 15 | 0 | 7 | 5 |
| Macroglia | 2 | 3 | 202 | 189 | 76 | 98 | 24 |
| Microglia | 2 | 5 | 2 | 1 | 13 | 14 | 7 |
| Macrophages | 1 | 2 | 12 | 14 | 0 | 0 | 2 |
| Retinal progenitor cells | 20 | 31 | 125 | 186 | 42 | 94 | 109 |
| Photoreceptors | 298 | 321 | 321 | 123 | 585 | 709 | 303 |
| Interneurons | 168 | 122 | 142 | 18 | 240 | 310 | 169 |
| Ganglion cells | 38 | 68 | 12 | 1 | 67 | 24 | 63 |
| Unspecific cell types | 17 | 78 | 29 | 308 | 18 | 5 | 13 |
|  |  |  |  |  |  |  |  |
|  | slide2_006 | slide2_007 | slide2_008 | slide2_009 | slide2_010 | slide3_001 | slide3_002 |
| Choroid-RPE complex | 309 | 193 | 200 | 363 | 227 | 232 | 167 |
| Perivascular cells | 4 | 1 | 38 | 0 | 1 | 23 | 21 |
| Macroglia | 30 | 44 | 19 | 24 | 28 | 231 | 151 |
| Microglia | 2 | 8 | 1 | 4 | 9 | 5 | 2 |
| Macrophages | 1 | 3 | 11 | 2 | 0 | 11 | 23 |
| Retinal progenitor cells | 71 | 78 | 60 | 40 | 54 | 113 | 78 |
| Photoreceptors | 312 | 438 | 177 | 234 | 361 | 293 | 281 |
| Interneurons | 214 | 239 | 132 | 190 | 188 | 138 | 142 |
| Ganglion cells | 68 | 60 | 42 | 49 | 44 | 8 | 21 |
| Unspecific cell types | 5 | 8 | 312 | 6 | 13 | 35 | 15 |
|  |  |  |  |  |  |  |  |
|  | slide3_003 | slide3_004 | slide3_005 | slide3_006 | slide3_007 | slide3_008 | slide3_009 |
| Choroid-RPE complex | 83 | 127 | 267 | 298 | 296 | 301 | 307 |
| Perivascular cells | 1 | 6 | 3 | 3 | 14 | 0 | 2 |
| Macroglia | 52 | 66 | 15 | 2 | 4 | 10 | 11 |
| Microglia | 2 | 4 | 12 | 3 | 0 | 3 | 0 |
| Macrophages | 0 | 0 | 6 | 5 | 4 | 5 | 3 |
| Retinal progenitor cells | 18 | 48 | 60 | 21 | 5 | 13 | 1 |
| Photoreceptors | 717 | 822 | 247 | 238 | 324 | 323 | 395 |
| Interneurons | 295 | 342 | 164 | 151 | 180 | 234 | 188 |
| Ganglion cells | 73 | 51 | 89 | 36 | 46 | 36 | 45 |
| Unspecific cell types | 0 | 3 | 8 | 92 | 16 | 17 | 15 |

| **Table S9. IMC sample metadata** | | | | | | | |
| --- | --- | --- | --- | --- | --- | --- | --- |
| sample_id | donor_id | roi_id | condition | sample_id | donor_id | roi_id | condition |
| day1_002 | 1 | 1 | LC | day1_004 | 2 | 3 | C |
| day1_003 | 1 | 2 | LC | day1_005 | 2 | 4 | C |
| day1_004 | 2 | 3 | BC | slide2_003 | 6 | 11 | C |
| day1_005 | 2 | 4 | BC | slide2_004 | 6 | 12 | C |
| day1_006 | 3 | 5 | Fas | slide3_003 | 10 | 21 | C |
| day1_007 | 3 | 6 | Fas | slide3_004 | 10 | 22 | C |
| day1_008 | 4 | 7 | Bel | day1_002 | 1 | 1 | LC |
| day1_009 | 4 | 8 | Bel | day1_003 | 1 | 2 | LC |
| slide2_001 | 5 | 9 | LC | slide2_001 | 5 | 9 | LC |
| slide2_002 | 5 | 10 | LC | slide2_002 | 5 | 10 | LC |
| slide2_003 | 6 | 11 | BC | slide3_001 | 9 | 19 | LC |
| slide2_004 | 6 | 12 | BC | slide3_002 | 9 | 20 | LC |
| slide2_005 | 7 | 13 | Fas | day1_006 | 3 | 5 | Fas |
| slide2_006 | 7 | 14 | Fas | day1_007 | 3 | 6 | Fas |
| slide2_007 | 7 | 15 | Fas | slide2_005 | 7 | 13 | Fas |
| slide2_008 | 7 | 16 | Fas | slide2_006 | 7 | 14 | Fas |
| slide2_009 | 8 | 17 | Bel | slide2_007 | 7 | 15 | Fas |
| slide2_010 | 8 | 18 | Bel | slide2_008 | 7 | 16 | Fas |
| slide3_001 | 9 | 19 | LC | slide3_005 | 11 | 23 | Fas |
| slide3_002 | 9 | 20 | LC | slide3_006 | 11 | 24 | Fas |
| slide3_003 | 10 | 21 | BC | day1_008 | 4 | 7 | Bel |
| slide3_004 | 10 | 22 | BC | day1_009 | 4 | 8 | Bel |
| slide3_005 | 11 | 23 | Fas | slide2_009 | 8 | 17 | Bel |
| slide3_006 | 11 | 24 | Fas | slide2_010 | 8 | 18 | Bel |
| slide3_007 | 12 | 25 | Bel | slide3_007 | 12 | 25 | Bel |
| slide3_008 | 12 | 26 | Bel | slide3_008 | 12 | 26 | Bel |
| slide3_009 | 12 | 27 | Bel | slide3_009 | 12 | 27 | Bel |

| **Table S10. Celltype neighborhoods** | | | | | | | | | | | | |
| --- | --- | --- | --- | --- | --- | --- | --- | --- | --- | --- | --- | --- |
| , | = | day1_002 | |  |  |  |  |  |  |  |  |  |
|  |  |  |  |  |  |  |  |  |  |  |  |  |
|  |  |  |  |  |  |  |  |  |  |  |  |  |
| 1 | 2 | 3 | 4 | 5 | 6 | 7 | 8 | 9 | 10 |  |  |  |
| Choroid-RPE | complex | 147 | 0 | 1 | 0 | 0 | 0 | 0 | 0 | 1 | 0 |  |
| Perivascular | cells | 0 | 6 | 0 | 0 | 0 | 0 | 0 | 0 | 4 | 0 |  |
| Macroglia | 1 | 92 | 17 | 1 | 5 | 60 | 0 | 2 | 6 | 10 |  |  |
| Microglia | 1 | 2 | 1 | 0 | 0 | 0 | 0 | 0 | 0 | 0 |  |  |
| Macrophages | 1 | 0 | 0 | 0 | 0 | 0 | 0 | 0 | 0 | 0 |  |  |
| Retinal | progenitor | cells | 1 | 0 | 0 | 27 | 0 | 0 | 0 | 36 | 0 | 1 |
| Photoreceptors | 0 | 0 | 0 | 42 | 0 | 1 | 0 | 323 | 0 | 0 |  |  |
| Interneurons | 0 | 6 | 69 | 1 | 0 | 64 | 0 | 0 | 0 | 0 |  |  |
| Ganglion | cells | 0 | 6 | 0 | 0 | 0 | 0 | 0 | 0 | 0 | 1 |  |
| Unspecific | cell | types | 1 | 4 | 0 | 0 | 9 | 2 | 0 | 1 | 0 | 4 |
|  |  |  |  |  |  |  |  |  |  |  |  |  |
| , | = | day1_003 | |  |  |  |  |  |  |  |  |  |
|  |  |  |  |  |  |  |  |  |  |  |  |  |
|  |  |  |  |  |  |  |  |  |  |  |  |  |
| 1 | 2 | 3 | 4 | 5 | 6 | 7 | 8 | 9 | 10 |  |  |  |
| Choroid-RPE | complex | 141 | 0 | 0 | 0 | 0 | 0 | 0 | 0 | 5 | 0 |  |
| Perivascular | cells | 0 | 2 | 0 | 0 | 4 | 0 | 0 | 0 | 3 | 0 |  |
| Macroglia | 0 | 96 | 6 | 8 | 4 | 46 | 0 | 0 | 3 | 3 |  |  |
| Microglia | 1 | 1 | 0 | 2 | 1 | 0 | 0 | 0 | 0 | 0 |  |  |
| Macrophages | 9 | 0 | 0 | 0 | 3 | 0 | 0 | 0 | 3 | 0 |  |  |
| Retinal | progenitor | cells | 0 | 0 | 0 | 134 | 0 | 2 | 0 | 36 | 0 | 0 |
| Photoreceptors | 0 | 0 | 0 | 99 | 0 | 0 | 0 | 177 | 0 | 0 |  |  |
| Interneurons | 0 | 18 | 16 | 0 | 0 | 25 | 0 | 0 | 0 | 0 |  |  |
| Ganglion | cells | 0 | 1 | 0 | 0 | 1 | 0 | 4 | 0 | 0 | 3 |  |
| Unspecific | cell | types | 0 | 2 | 0 | 1 | 84 | 1 | 0 | 0 | 1 | 1 |
|  |  |  |  |  |  |  |  |  |  |  |  |  |
| , | = | day1_004 | |  |  |  |  |  |  |  |  |  |
|  |  |  |  |  |  |  |  |  |  |  |  |  |
|  |  |  |  |  |  |  |  |  |  |  |  |  |
| 1 | 2 | 3 | 4 | 5 | 6 | 7 | 8 | 9 | 10 |  |  |  |
| Choroid-RPE | complex | 64 | 0 | 0 | 0 | 0 | 0 | 0 | 1 | 0 | 0 |  |
| Perivascular | cells | 0 | 0 | 0 | 0 | 0 | 0 | 0 | 0 | 0 | 0 |  |
| Macroglia | 0 | 39 | 18 | 0 | 0 | 26 | 6 | 0 | 0 | 15 |  |  |
| Microglia | 4 | 0 | 12 | 0 | 2 | 8 | 2 | 10 | 0 | 6 |  |  |
| Macrophages | 0 | 0 | 0 | 0 | 0 | 0 | 0 | 0 | 0 | 0 |  |  |
| Retinal | progenitor | cells | 0 | 0 | 0 | 2 | 0 | 1 | 0 | 2 | 0 | 0 |
| Photoreceptors | 0 | 0 | 0 | 1 | 0 | 0 | 0 | 583 | 0 | 0 |  |  |
| Interneurons | 0 | 1 | 220 | 1 | 0 | 32 | 0 | 1 | 0 | 0 |  |  |
| Ganglion | cells | 0 | 3 | 0 | 0 | 0 | 0 | 30 | 0 | 0 | 27 |  |
| Unspecific | cell | types | 0 | 1 | 2 | 0 | 7 | 1 | 0 | 3 | 0 | 3 |
|  |  |  |  |  |  |  |  |  |  |  |  |  |
| , | = | day1_005 | |  |  |  |  |  |  |  |  |  |
|  |  |  |  |  |  |  |  |  |  |  |  |  |
|  |  |  |  |  |  |  |  |  |  |  |  |  |
| 1 | 2 | 3 | 4 | 5 | 6 | 7 | 8 | 9 | 10 |  |  |  |
| Choroid-RPE | complex | 57 | 0 | 0 | 0 | 0 | 0 | 0 | 3 | 0 | 0 |  |
| Perivascular | cells | 0 | 0 | 0 | 0 | 0 | 0 | 0 | 0 | 0 | 0 |  |
| Macroglia | 0 | 14 | 9 | 1 | 6 | 42 | 6 | 3 | 0 | 15 |  |  |
| Microglia | 0 | 1 | 6 | 0 | 1 | 6 | 3 | 1 | 0 | 2 |  |  |
| Macrophages | 0 | 0 | 0 | 0 | 0 | 0 | 0 | 0 | 0 | 0 |  |  |
| Retinal | progenitor | cells | 0 | 1 | 0 | 3 | 0 | 5 | 0 | 5 | 0 | 0 |
| Photoreceptors | 0 | 1 | 0 | 8 | 0 | 0 | 0 | 601 | 0 | 0 |  |  |
| Interneurons | 0 | 1 | 237 | 0 | 0 | 30 | 0 | 0 | 0 | 1 |  |  |
| Ganglion | cells | 0 | 1 | 0 | 0 | 0 | 2 | 64 | 0 | 0 | 22 |  |
| Unspecific | cell | types | 0 | 0 | 3 | 3 | 22 | 2 | 0 | 2 | 0 | 1 |
|  |  |  |  |  |  |  |  |  |  |  |  |  |
| , | = | day1_006 | |  |  |  |  |  |  |  |  |  |
|  |  |  |  |  |  |  |  |  |  |  |  |  |
|  |  |  |  |  |  |  |  |  |  |  |  |  |
| 1 | 2 | 3 | 4 | 5 | 6 | 7 | 8 | 9 | 10 |  |  |  |
| Choroid-RPE | complex | 101 | 0 | 0 | 0 | 3 | 0 | 0 | 0 | 4 | 0 |  |
| Perivascular | cells | 0 | 0 | 0 | 0 | 2 | 0 | 0 | 0 | 0 | 0 |  |
| Macroglia | 0 | 0 | 3 | 1 | 3 | 12 | 5 | 2 | 0 | 3 |  |  |
| Microglia | 0 | 0 | 0 | 0 | 0 | 0 | 0 | 1 | 0 | 0 |  |  |
| Macrophages | 7 | 0 | 0 | 0 | 3 | 0 | 0 | 0 | 0 | 0 |  |  |
| Retinal | progenitor | cells | 0 | 0 | 5 | 44 | 0 | 3 | 0 | 6 | 0 | 0 |
| Photoreceptors | 0 | 0 | 1 | 19 | 0 | 0 | 0 | 331 | 0 | 0 |  |  |
| Interneurons | 0 | 0 | 157 | 1 | 0 | 11 | 0 | 0 | 0 | 0 |  |  |
| Ganglion | cells | 0 | 0 | 0 | 0 | 0 | 0 | 54 | 0 | 0 | 3 |  |
| Unspecific | cell | types | 0 | 4 | 1 | 4 | 75 | 2 | 0 | 3 | 1 | 1 |
|  |  |  |  |  |  |  |  |  |  |  |  |  |
| , | = | day1_007 | |  |  |  |  |  |  |  |  |  |
|  |  |  |  |  |  |  |  |  |  |  |  |  |
|  |  |  |  |  |  |  |  |  |  |  |  |  |
| 1 | 2 | 3 | 4 | 5 | 6 | 7 | 8 | 9 | 10 |  |  |  |
| Choroid-RPE | complex | 369 | 0 | 0 | 0 | 0 | 0 | 0 | 1 | 2 | 0 |  |
| Perivascular | cells | 2 | 0 | 0 | 0 | 0 | 0 | 0 | 0 | 3 | 0 |  |
| Macroglia | 1 | 0 | 3 | 1 | 0 | 2 | 1 | 2 | 0 | 0 |  |  |
| Microglia | 1 | 0 | 0 | 0 | 0 | 0 | 0 | 1 | 0 | 0 |  |  |
| Macrophages | 4 | 0 | 0 | 0 | 0 | 0 | 0 | 0 | 1 | 0 |  |  |
| Retinal | progenitor | cells | 0 | 0 | 6 | 18 | 0 | 2 | 1 | 2 | 0 | 0 |
| Photoreceptors | 0 | 0 | 0 | 6 | 0 | 0 | 0 | 310 | 0 | 0 |  |  |
| Interneurons | 0 | 0 | 167 | 4 | 0 | 10 | 0 | 1 | 0 | 0 |  |  |
| Ganglion | cells | 0 | 0 | 2 | 0 | 0 | 0 | 32 | 0 | 0 | 1 |  |
| Unspecific | cell | types | 0 | 0 | 1 | 1 | 1 | 0 | 0 | 2 | 0 | 0 |
|  |  |  |  |  |  |  |  |  |  |  |  |  |
| , | = | day1_008 | |  |  |  |  |  |  |  |  |  |
|  |  |  |  |  |  |  |  |  |  |  |  |  |
|  |  |  |  |  |  |  |  |  |  |  |  |  |
| 1 | 2 | 3 | 4 | 5 | 6 | 7 | 8 | 9 | 10 |  |  |  |
| Choroid-RPE | complex | 318 | 0 | 0 | 0 | 0 | 0 | 0 | 0 | 0 | 0 |  |
| Perivascular | cells | 2 | 0 | 0 | 0 | 0 | 0 | 0 | 0 | 5 | 0 |  |
| Macroglia | 0 | 0 | 0 | 0 | 0 | 2 | 0 | 0 | 0 | 0 |  |  |
| Microglia | 1 | 0 | 1 | 0 | 0 | 0 | 0 | 0 | 0 | 0 |  |  |
| Macrophages | 1 | 0 | 0 | 0 | 0 | 0 | 0 | 0 | 0 | 0 |  |  |
| Retinal | progenitor | cells | 0 | 0 | 5 | 8 | 0 | 0 | 0 | 7 | 0 | 0 |
| Photoreceptors | 0 | 0 | 1 | 8 | 0 | 4 | 0 | 285 | 0 | 0 |  |  |
| Interneurons | 0 | 0 | 158 | 4 | 0 | 4 | 0 | 2 | 0 | 0 |  |  |
| Ganglion | cells | 0 | 0 | 2 | 0 | 0 | 0 | 33 | 0 | 1 | 2 |  |
| Unspecific | cell | types | 0 | 0 | 1 | 0 | 4 | 3 | 0 | 9 | 0 | 0 |
|  |  |  |  |  |  |  |  |  |  |  |  |  |
| , | = | day1_009 | |  |  |  |  |  |  |  |  |  |
|  |  |  |  |  |  |  |  |  |  |  |  |  |
|  |  |  |  |  |  |  |  |  |  |  |  |  |
| 1 | 2 | 3 | 4 | 5 | 6 | 7 | 8 | 9 | 10 |  |  |  |
| Choroid-RPE | complex | 200 | 0 | 0 | 1 | 0 | 0 | 0 | 0 | 0 | 0 |  |
| Perivascular | cells | 0 | 0 | 0 | 0 | 1 | 0 | 0 | 0 | 0 | 0 |  |
| Macroglia | 0 | 0 | 1 | 0 | 0 | 0 | 0 | 2 | 0 | 0 |  |  |
| Microglia | 2 | 0 | 1 | 1 | 1 | 0 | 0 | 0 | 0 | 0 |  |  |
| Macrophages | 0 | 0 | 0 | 0 | 1 | 0 | 1 | 0 | 0 | 0 |  |  |
| Retinal | progenitor | cells | 0 | 0 | 1 | 22 | 0 | 0 | 0 | 8 | 0 | 0 |
| Photoreceptors | 0 | 0 | 1 | 14 | 0 | 0 | 0 | 306 | 0 | 0 |  |  |
| Interneurons | 0 | 0 | 115 | 1 | 0 | 2 | 1 | 2 | 0 | 1 |  |  |
| Ganglion | cells | 0 | 0 | 0 | 0 | 2 | 0 | 62 | 0 | 0 | 4 |  |
| Unspecific | cell | types | 0 | 0 | 0 | 0 | 78 | 0 | 0 | 0 | 0 | 0 |
|  |  |  |  |  |  |  |  |  |  |  |  |  |
| , | = | slide2_001 | |  |  |  |  |  |  |  |  |  |
|  |  |  |  |  |  |  |  |  |  |  |  |  |
|  |  |  |  |  |  |  |  |  |  |  |  |  |
| 1 | 2 | 3 | 4 | 5 | 6 | 7 | 8 | 9 | 10 |  |  |  |
| Choroid-RPE | complex | 182 | 0 | 0 | 1 | 0 | 0 | 0 | 0 | 4 | 0 |  |
| Perivascular | cells | 1 | 5 | 0 | 0 | 0 | 0 | 0 | 0 | 1 | 2 |  |
| Macroglia | 0 | 111 | 14 | 8 | 2 | 54 | 0 | 0 | 4 | 9 |  |  |
| Microglia | 0 | 0 | 0 | 1 | 0 | 1 | 0 | 0 | 0 | 0 |  |  |
| Macrophages | 4 | 0 | 0 | 0 | 0 | 0 | 0 | 3 | 5 | 0 |  |  |
| Retinal | progenitor | cells | 0 | 3 | 0 | 89 | 0 | 8 | 0 | 25 | 0 | 0 |
| Photoreceptors | 0 | 1 | 0 | 54 | 0 | 1 | 0 | 264 | 1 | 0 |  |  |
| Interneurons | 0 | 10 | 72 | 0 | 0 | 60 | 0 | 0 | 0 | 0 |  |  |
| Ganglion | cells | 0 | 8 | 0 | 0 | 0 | 0 | 0 | 0 | 0 | 4 |  |
| Unspecific | cell | types | 1 | 0 | 0 | 0 | 24 | 3 | 0 | 1 | 0 | 0 |
|  |  |  |  |  |  |  |  |  |  |  |  |  |
| , | = | slide2_002 | |  |  |  |  |  |  |  |  |  |
|  |  |  |  |  |  |  |  |  |  |  |  |  |
|  |  |  |  |  |  |  |  |  |  |  |  |  |
| 1 | 2 | 3 | 4 | 5 | 6 | 7 | 8 | 9 | 10 |  |  |  |
| Choroid-RPE | complex | 140 | 0 | 0 | 1 | 2 | 0 | 0 | 0 | 0 | 0 |  |
| Perivascular | cells | 1 | 1 | 0 | 0 | 11 | 0 | 0 | 0 | 2 | 0 |  |
| Macroglia | 0 | 158 | 3 | 4 | 9 | 15 | 0 | 0 | 0 | 0 |  |  |
| Microglia | 0 | 1 | 0 | 0 | 0 | 0 | 0 | 0 | 0 | 0 |  |  |
| Macrophages | 0 | 0 | 0 | 0 | 9 | 0 | 0 | 0 | 5 | 0 |  |  |
| Retinal | progenitor | cells | 0 | 4 | 0 | 169 | 2 | 0 | 0 | 11 | 0 | 0 |
| Photoreceptors | 0 | 0 | 0 | 62 | 0 | 0 | 0 | 61 | 0 | 0 |  |  |
| Interneurons | 0 | 3 | 0 | 0 | 1 | 14 | 0 | 0 | 0 | 0 |  |  |
| Ganglion | cells | 0 | 1 | 0 | 0 | 0 | 0 | 0 | 0 | 0 | 0 |  |
| Unspecific | cell | types | 1 | 2 | 0 | 1 | 299 | 0 | 0 | 0 | 5 | 0 |
|  |  |  |  |  |  |  |  |  |  |  |  |  |
| , | = | slide2_003 | |  |  |  |  |  |  |  |  |  |
|  |  |  |  |  |  |  |  |  |  |  |  |  |
|  |  |  |  |  |  |  |  |  |  |  |  |  |
| 1 | 2 | 3 | 4 | 5 | 6 | 7 | 8 | 9 | 10 |  |  |  |
| Choroid-RPE | complex | 50 | 0 | 0 | 3 | 0 | 0 | 0 | 0 | 0 | 0 |  |
| Perivascular | cells | 0 | 0 | 0 | 0 | 0 | 0 | 0 | 0 | 0 | 0 |  |
| Macroglia | 0 | 10 | 13 | 0 | 3 | 33 | 7 | 4 | 0 | 6 |  |  |
| Microglia | 1 | 0 | 3 | 1 | 1 | 0 | 3 | 1 | 0 | 3 |  |  |
| Macrophages | 0 | 0 | 0 | 0 | 0 | 0 | 0 | 0 | 0 | 0 |  |  |
| Retinal | progenitor | cells | 0 | 0 | 0 | 15 | 1 | 1 | 0 | 25 | 0 | 0 |
| Photoreceptors | 0 | 0 | 0 | 28 | 0 | 0 | 0 | 557 | 0 | 0 |  |  |
| Interneurons | 0 | 2 | 202 | 1 | 0 | 34 | 0 | 1 | 0 | 0 |  |  |
| Ganglion | cells | 0 | 0 | 2 | 0 | 0 | 1 | 47 | 0 | 0 | 17 |  |
| Unspecific | cell | types | 0 | 2 | 0 | 4 | 3 | 2 | 1 | 5 | 0 | 1 |
|  |  |  |  |  |  |  |  |  |  |  |  |  |
| , | = | slide2_004 | |  |  |  |  |  |  |  |  |  |
|  |  |  |  |  |  |  |  |  |  |  |  |  |
|  |  |  |  |  |  |  |  |  |  |  |  |  |
| 1 | 2 | 3 | 4 | 5 | 6 | 7 | 8 | 9 | 10 |  |  |  |
| Choroid-RPE | complex | 92 | 0 | 0 | 4 | 0 | 0 | 0 | 0 | 0 | 0 |  |
| Perivascular | cells | 0 | 1 | 0 | 0 | 0 | 0 | 2 | 0 | 1 | 3 |  |
| Macroglia | 0 | 37 | 5 | 0 | 0 | 35 | 0 | 0 | 1 | 20 |  |  |
| Microglia | 2 | 7 | 4 | 0 | 0 | 0 | 0 | 0 | 0 | 1 |  |  |
| Macrophages | 0 | 0 | 0 | 0 | 0 | 0 | 0 | 0 | 0 | 0 |  |  |
| Retinal | progenitor | cells | 0 | 0 | 6 | 64 | 0 | 2 | 0 | 21 | 0 | 1 |
| Photoreceptors | 0 | 0 | 0 | 44 | 0 | 0 | 0 | 665 | 0 | 0 |  |  |
| Interneurons | 0 | 2 | 281 | 1 | 0 | 26 | 0 | 0 | 0 | 0 |  |  |
| Ganglion | cells | 0 | 7 | 0 | 0 | 0 | 1 | 0 | 0 | 3 | 13 |  |
| Unspecific | cell | types | 0 | 1 | 0 | 0 | 4 | 0 | 0 | 0 | 0 | 0 |
|  |  |  |  |  |  |  |  |  |  |  |  |  |
| , | = | slide2_005 | |  |  |  |  |  |  |  |  |  |
|  |  |  |  |  |  |  |  |  |  |  |  |  |
|  |  |  |  |  |  |  |  |  |  |  |  |  |
| 1 | 2 | 3 | 4 | 5 | 6 | 7 | 8 | 9 | 10 |  |  |  |
| Choroid-RPE | complex | 292 | 0 | 0 | 4 | 0 | 0 | 0 | 0 | 2 | 0 |  |
| Perivascular | cells | 5 | 0 | 0 | 0 | 0 | 0 | 0 | 0 | 0 | 0 |  |
| Macroglia | 0 | 0 | 4 | 3 | 2 | 12 | 3 | 0 | 0 | 0 |  |  |
| Microglia | 0 | 0 | 1 | 5 | 1 | 0 | 0 | 0 | 0 | 0 |  |  |
| Macrophages | 2 | 0 | 0 | 0 | 0 | 0 | 0 | 0 | 0 | 0 |  |  |
| Retinal | progenitor | cells | 0 | 0 | 14 | 80 | 0 | 5 | 0 | 10 | 0 | 0 |
| Photoreceptors | 0 | 1 | 1 | 24 | 0 | 1 | 0 | 276 | 0 | 0 |  |  |
| Interneurons | 0 | 1 | 134 | 12 | 1 | 18 | 0 | 3 | 0 | 0 |  |  |
| Ganglion | cells | 0 | 0 | 1 | 0 | 0 | 0 | 61 | 0 | 0 | 1 |  |
| Unspecific | cell | types | 0 | 1 | 0 | 1 | 5 | 4 | 0 | 2 | 0 | 0 |
|  |  |  |  |  |  |  |  |  |  |  |  |  |
| , | = | slide2_006 | |  |  |  |  |  |  |  |  |  |
|  |  |  |  |  |  |  |  |  |  |  |  |  |
|  |  |  |  |  |  |  |  |  |  |  |  |  |
| 1 | 2 | 3 | 4 | 5 | 6 | 7 | 8 | 9 | 10 |  |  |  |
| Choroid-RPE | complex | 308 | 0 | 0 | 1 | 0 | 0 | 0 | 0 | 0 | 0 |  |
| Perivascular | cells | 1 | 0 | 0 | 0 | 0 | 0 | 0 | 0 | 0 | 3 |  |
| Macroglia | 0 | 0 | 10 | 2 | 0 | 9 | 2 | 6 | 1 | 0 |  |  |
| Microglia | 1 | 0 | 1 | 0 | 0 | 0 | 0 | 0 | 0 | 0 |  |  |
| Macrophages | 1 | 0 | 0 | 0 | 0 | 0 | 0 | 0 | 0 | 0 |  |  |
| Retinal | progenitor | cells | 0 | 0 | 6 | 54 | 0 | 5 | 0 | 6 | 0 | 0 |
| Photoreceptors | 0 | 0 | 0 | 13 | 0 | 2 | 0 | 297 | 0 | 0 |  |  |
| Interneurons | 0 | 0 | 187 | 8 | 0 | 19 | 0 | 0 | 0 | 0 |  |  |
| Ganglion | cells | 0 | 0 | 2 | 0 | 0 | 1 | 61 | 0 | 0 | 4 |  |
| Unspecific | cell | types | 0 | 0 | 0 | 2 | 0 | 2 | 0 | 1 | 0 | 0 |
|  |  |  |  |  |  |  |  |  |  |  |  |  |
| , | = | slide2_007 | |  |  |  |  |  |  |  |  |  |
|  |  |  |  |  |  |  |  |  |  |  |  |  |
|  |  |  |  |  |  |  |  |  |  |  |  |  |
| 1 | 2 | 3 | 4 | 5 | 6 | 7 | 8 | 9 | 10 |  |  |  |
| Choroid-RPE | complex | 192 | 0 | 0 | 1 | 0 | 0 | 0 | 0 | 0 | 0 |  |
| Perivascular | cells | 1 | 0 | 0 | 0 | 0 | 0 | 0 | 0 | 0 | 0 |  |
| Macroglia | 0 | 8 | 8 | 3 | 1 | 19 | 3 | 2 | 0 | 0 |  |  |
| Microglia | 1 | 0 | 0 | 7 | 0 | 0 | 0 | 0 | 0 | 0 |  |  |
| Macrophages | 3 | 0 | 0 | 0 | 0 | 0 | 0 | 0 | 0 | 0 |  |  |
| Retinal | progenitor | cells | 0 | 0 | 9 | 50 | 0 | 5 | 0 | 13 | 0 | 1 |
| Photoreceptors | 0 | 0 | 0 | 26 | 0 | 0 | 0 | 412 | 0 | 0 |  |  |
| Interneurons | 0 | 0 | 206 | 3 | 1 | 29 | 0 | 0 | 0 | 0 |  |  |
| Ganglion | cells | 0 | 0 | 2 | 0 | 0 | 0 | 55 | 0 | 0 | 3 |  |
| Unspecific | cell | types | 0 | 0 | 1 | 0 | 0 | 5 | 0 | 2 | 0 | 0 |
|  |  |  |  |  |  |  |  |  |  |  |  |  |
| , | = | slide2_008 | |  |  |  |  |  |  |  |  |  |
|  |  |  |  |  |  |  |  |  |  |  |  |  |
|  |  |  |  |  |  |  |  |  |  |  |  |  |
| 1 | 2 | 3 | 4 | 5 | 6 | 7 | 8 | 9 | 10 |  |  |  |
| Choroid-RPE | complex | 195 | 0 | 0 | 0 | 1 | 1 | 0 | 3 | 0 | 0 |  |
| Perivascular | cells | 1 | 0 | 0 | 0 | 0 | 0 | 0 | 0 | 34 | 3 |  |
| Macroglia | 0 | 4 | 4 | 0 | 5 | 4 | 1 | 1 | 0 | 0 |  |  |
| Microglia | 1 | 0 | 0 | 0 | 0 | 0 | 0 | 0 | 0 | 0 |  |  |
| Macrophages | 0 | 0 | 0 | 0 | 7 | 0 | 0 | 0 | 4 | 0 |  |  |
| Retinal | progenitor | cells | 1 | 0 | 7 | 45 | 2 | 1 | 0 | 4 | 0 | 0 |
| Photoreceptors | 0 | 0 | 0 | 11 | 3 | 3 | 0 | 160 | 0 | 0 |  |  |
| Interneurons | 0 | 0 | 115 | 5 | 0 | 11 | 0 | 1 | 0 | 0 |  |  |
| Ganglion | cells | 0 | 0 | 1 | 0 | 1 | 0 | 34 | 0 | 1 | 5 |  |
| Unspecific | cell | types | 3 | 1 | 2 | 0 | 290 | 4 | 0 | 2 | 9 | 1 |
|  |  |  |  |  |  |  |  |  |  |  |  |  |
| , | = | slide2_009 | |  |  |  |  |  |  |  |  |  |
|  |  |  |  |  |  |  |  |  |  |  |  |  |
|  |  |  |  |  |  |  |  |  |  |  |  |  |
| 1 | 2 | 3 | 4 | 5 | 6 | 7 | 8 | 9 | 10 |  |  |  |
| Choroid-RPE | complex | 362 | 0 | 0 | 0 | 1 | 0 | 0 | 0 | 0 | 0 |  |
| Perivascular | cells | 0 | 0 | 0 | 0 | 0 | 0 | 0 | 0 | 0 | 0 |  |
| Macroglia | 0 | 0 | 5 | 3 | 0 | 12 | 0 | 4 | 0 | 0 |  |  |
| Microglia | 2 | 0 | 0 | 1 | 0 | 1 | 0 | 0 | 0 | 0 |  |  |
| Macrophages | 2 | 0 | 0 | 0 | 0 | 0 | 0 | 0 | 0 | 0 |  |  |
| Retinal | progenitor | cells | 0 | 0 | 9 | 21 | 0 | 2 | 0 | 8 | 0 | 0 |
| Photoreceptors | 0 | 2 | 1 | 16 | 0 | 1 | 0 | 214 | 0 | 0 |  |  |
| Interneurons | 0 | 1 | 164 | 4 | 0 | 18 | 0 | 3 | 0 | 0 |  |  |
| Ganglion | cells | 0 | 0 | 0 | 0 | 0 | 0 | 49 | 0 | 0 | 0 |  |
| Unspecific | cell | types | 1 | 0 | 0 | 0 | 1 | 1 | 1 | 2 | 0 | 0 |
|  |  |  |  |  |  |  |  |  |  |  |  |  |
| , | = | slide2_010 | |  |  |  |  |  |  |  |  |  |
|  |  |  |  |  |  |  |  |  |  |  |  |  |
|  |  |  |  |  |  |  |  |  |  |  |  |  |
| 1 | 2 | 3 | 4 | 5 | 6 | 7 | 8 | 9 | 10 |  |  |  |
| Choroid-RPE | complex | 224 | 3 | 0 | 0 | 0 | 0 | 0 | 0 | 0 | 0 |  |
| Perivascular | cells | 0 | 0 | 0 | 0 | 0 | 0 | 1 | 0 | 0 | 0 |  |
| Macroglia | 4 | 0 | 2 | 4 | 0 | 5 | 1 | 10 | 0 | 2 |  |  |
| Microglia | 8 | 0 | 0 | 1 | 0 | 0 | 0 | 0 | 0 | 0 |  |  |
| Macrophages | 0 | 0 | 0 | 0 | 0 | 0 | 0 | 0 | 0 | 0 |  |  |
| Retinal | progenitor | cells | 0 | 0 | 5 | 34 | 0 | 6 | 0 | 9 | 0 | 0 |
| Photoreceptors | 0 | 0 | 1 | 24 | 0 | 5 | 0 | 331 | 0 | 0 |  |  |
| Interneurons | 0 | 0 | 167 | 4 | 0 | 14 | 0 | 3 | 0 | 0 |  |  |
| Ganglion | cells | 0 | 0 | 1 | 0 | 0 | 0 | 40 | 0 | 0 | 3 |  |
| Unspecific | cell | types | 3 | 0 | 1 | 2 | 0 | 3 | 0 | 4 | 0 | 0 |
|  |  |  |  |  |  |  |  |  |  |  |  |  |
| , | = | slide3_001 | |  |  |  |  |  |  |  |  |  |
|  |  |  |  |  |  |  |  |  |  |  |  |  |
|  |  |  |  |  |  |  |  |  |  |  |  |  |
| 1 | 2 | 3 | 4 | 5 | 6 | 7 | 8 | 9 | 10 |  |  |  |
| Choroid-RPE | complex | 225 | 0 | 0 | 1 | 0 | 0 | 0 | 0 | 6 | 0 |  |
| Perivascular | cells | 2 | 7 | 0 | 0 | 0 | 0 | 0 | 0 | 14 | 0 |  |
| Macroglia | 0 | 133 | 19 | 3 | 10 | 55 | 1 | 0 | 5 | 5 |  |  |
| Microglia | 0 | 0 | 0 | 3 | 0 | 2 | 0 | 0 | 0 | 0 |  |  |
| Macrophages | 11 | 0 | 0 | 0 | 0 | 0 | 0 | 0 | 0 | 0 |  |  |
| Retinal | progenitor | cells | 1 | 4 | 1 | 77 | 0 | 5 | 0 | 25 | 0 | 0 |
| Photoreceptors | 0 | 0 | 1 | 47 | 1 | 2 | 0 | 242 | 0 | 0 |  |  |
| Interneurons | 0 | 11 | 62 | 2 | 0 | 61 | 0 | 2 | 0 | 0 |  |  |
| Ganglion | cells | 0 | 4 | 0 | 0 | 0 | 0 | 0 | 0 | 0 | 4 |  |
| Unspecific | cell | types | 3 | 1 | 1 | 0 | 26 | 0 | 0 | 4 | 0 | 0 |
|  |  |  |  |  |  |  |  |  |  |  |  |  |
| , | = | slide3_002 | |  |  |  |  |  |  |  |  |  |
|  |  |  |  |  |  |  |  |  |  |  |  |  |
|  |  |  |  |  |  |  |  |  |  |  |  |  |
| 1 | 2 | 3 | 4 | 5 | 6 | 7 | 8 | 9 | 10 |  |  |  |
| Choroid-RPE | complex | 158 | 0 | 0 | 1 | 0 | 0 | 0 | 1 | 7 | 0 |  |
| Perivascular | cells | 1 | 4 | 0 | 0 | 0 | 0 | 0 | 0 | 16 | 0 |  |
| Macroglia | 0 | 83 | 8 | 1 | 2 | 38 | 0 | 1 | 7 | 11 |  |  |
| Microglia | 0 | 0 | 0 | 1 | 0 | 0 | 0 | 1 | 0 | 0 |  |  |
| Macrophages | 7 | 0 | 2 | 0 | 0 | 0 | 1 | 1 | 11 | 1 |  |  |
| Retinal | progenitor | cells | 0 | 5 | 0 | 58 | 0 | 1 | 0 | 13 | 0 | 1 |
| Photoreceptors | 1 | 0 | 0 | 34 | 2 | 0 | 0 | 244 | 0 | 0 |  |  |
| Interneurons | 0 | 10 | 104 | 0 | 1 | 25 | 0 | 2 | 0 | 0 |  |  |
| Ganglion | cells | 0 | 3 | 0 | 0 | 0 | 0 | 5 | 0 | 1 | 12 |  |
| Unspecific | cell | types | 0 | 1 | 2 | 0 | 10 | 0 | 0 | 1 | 0 | 1 |
|  |  |  |  |  |  |  |  |  |  |  |  |  |
| , | = | slide3_003 | |  |  |  |  |  |  |  |  |  |
|  |  |  |  |  |  |  |  |  |  |  |  |  |
|  |  |  |  |  |  |  |  |  |  |  |  |  |
| 1 | 2 | 3 | 4 | 5 | 6 | 7 | 8 | 9 | 10 |  |  |  |
| Choroid-RPE | complex | 81 | 0 | 0 | 2 | 0 | 0 | 0 | 0 | 0 | 0 |  |
| Perivascular | cells | 0 | 0 | 0 | 0 | 0 | 0 | 1 | 0 | 0 | 0 |  |
| Macroglia | 0 | 6 | 21 | 2 | 0 | 11 | 7 | 4 | 0 | 1 |  |  |
| Microglia | 0 | 0 | 1 | 0 | 0 | 0 | 1 | 0 | 0 | 0 |  |  |
| Macrophages | 0 | 0 | 0 | 0 | 0 | 0 | 0 | 0 | 0 | 0 |  |  |
| Retinal | progenitor | cells | 0 | 0 | 0 | 4 | 0 | 0 | 0 | 14 | 0 | 0 |
| Photoreceptors | 0 | 0 | 0 | 7 | 0 | 0 | 0 | 710 | 0 | 0 |  |  |
| Interneurons | 0 | 0 | 271 | 0 | 0 | 24 | 0 | 0 | 0 | 0 |  |  |
| Ganglion | cells | 0 | 0 | 2 | 0 | 0 | 0 | 58 | 0 | 0 | 13 |  |
| Unspecific | cell | types | 0 | 0 | 0 | 0 | 0 | 0 | 0 | 0 | 0 | 0 |
|  |  |  |  |  |  |  |  |  |  |  |  |  |
| , | = | slide3_004 | |  |  |  |  |  |  |  |  |  |
|  |  |  |  |  |  |  |  |  |  |  |  |  |
|  |  |  |  |  |  |  |  |  |  |  |  |  |
| 1 | 2 | 3 | 4 | 5 | 6 | 7 | 8 | 9 | 10 |  |  |  |
| Choroid-RPE | complex | 122 | 0 | 0 | 4 | 0 | 0 | 0 | 1 | 0 | 0 |  |
| Perivascular | cells | 0 | 0 | 0 | 0 | 0 | 0 | 0 | 0 | 3 | 3 |  |
| Macroglia | 0 | 11 | 7 | 0 | 0 | 30 | 7 | 2 | 2 | 7 |  |  |
| Microglia | 0 | 0 | 1 | 0 | 0 | 0 | 1 | 1 | 1 | 0 |  |  |
| Macrophages | 0 | 0 | 0 | 0 | 0 | 0 | 0 | 0 | 0 | 0 |  |  |
| Retinal | progenitor | cells | 0 | 0 | 0 | 36 | 0 | 1 | 0 | 11 | 0 | 0 |
| Photoreceptors | 0 | 1 | 0 | 28 | 0 | 1 | 0 | 792 | 0 | 0 |  |  |
| Interneurons | 0 | 0 | 315 | 0 | 0 | 27 | 0 | 0 | 0 | 0 |  |  |
| Ganglion | cells | 0 | 1 | 3 | 0 | 0 | 0 | 32 | 0 | 1 | 14 |  |
| Unspecific | cell | types | 0 | 1 | 1 | 0 | 0 | 0 | 0 | 1 | 0 | 0 |
|  |  |  |  |  |  |  |  |  |  |  |  |  |
| , | = | slide3_005 | |  |  |  |  |  |  |  |  |  |
|  |  |  |  |  |  |  |  |  |  |  |  |  |
|  |  |  |  |  |  |  |  |  |  |  |  |  |
| 1 | 2 | 3 | 4 | 5 | 6 | 7 | 8 | 9 | 10 |  |  |  |
| Choroid-RPE | complex | 266 | 0 | 0 | 0 | 0 | 0 | 0 | 0 | 1 | 0 |  |
| Perivascular | cells | 1 | 0 | 0 | 0 | 0 | 0 | 0 | 0 | 0 | 2 |  |
| Macroglia | 1 | 1 | 3 | 0 | 0 | 5 | 4 | 1 | 0 | 0 |  |  |
| Microglia | 8 | 0 | 0 | 2 | 0 | 1 | 0 | 0 | 0 | 1 |  |  |
| Macrophages | 6 | 0 | 0 | 0 | 0 | 0 | 0 | 0 | 0 | 0 |  |  |
| Retinal | progenitor | cells | 0 | 0 | 9 | 43 | 0 | 1 | 1 | 6 | 0 | 0 |
| Photoreceptors | 0 | 0 | 0 | 14 | 0 | 1 | 0 | 232 | 0 | 0 |  |  |
| Interneurons | 0 | 0 | 150 | 7 | 1 | 6 | 0 | 0 | 0 | 0 |  |  |
| Ganglion | cells | 0 | 0 | 2 | 0 | 0 | 1 | 82 | 0 | 1 | 3 |  |
| Unspecific | cell | types | 0 | 0 | 2 | 1 | 0 | 5 | 0 | 0 | 0 | 0 |
|  |  |  |  |  |  |  |  |  |  |  |  |  |
| , | = | slide3_006 | |  |  |  |  |  |  |  |  |  |
|  |  |  |  |  |  |  |  |  |  |  |  |  |
|  |  |  |  |  |  |  |  |  |  |  |  |  |
| 1 | 2 | 3 | 4 | 5 | 6 | 7 | 8 | 9 | 10 |  |  |  |
| Choroid-RPE | complex | 297 | 0 | 0 | 0 | 0 | 0 | 0 | 1 | 0 | 0 |  |
| Perivascular | cells | 0 | 0 | 0 | 0 | 0 | 0 | 0 | 0 | 3 | 0 |  |
| Macroglia | 0 | 0 | 1 | 0 | 0 | 1 | 0 | 0 | 0 | 0 |  |  |
| Microglia | 0 | 0 | 0 | 1 | 0 | 0 | 0 | 1 | 0 | 1 |  |  |
| Macrophages | 1 | 0 | 0 | 0 | 3 | 0 | 1 | 0 | 0 | 0 |  |  |
| Retinal | progenitor | cells | 0 | 0 | 1 | 15 | 0 | 1 | 0 | 4 | 0 | 0 |
| Photoreceptors | 1 | 0 | 0 | 8 | 0 | 1 | 0 | 228 | 0 | 0 |  |  |
| Interneurons | 0 | 0 | 148 | 0 | 1 | 2 | 0 | 0 | 0 | 0 |  |  |
| Ganglion | cells | 0 | 0 | 1 | 0 | 1 | 0 | 30 | 0 | 0 | 4 |  |
| Unspecific | cell | types | 2 | 0 | 4 | 1 | 80 | 1 | 0 | 3 | 1 | 0 |
|  |  |  |  |  |  |  |  |  |  |  |  |  |
| , | = | slide3_007 | |  |  |  |  |  |  |  |  |  |
|  |  |  |  |  |  |  |  |  |  |  |  |  |
|  |  |  |  |  |  |  |  |  |  |  |  |  |
| 1 | 2 | 3 | 4 | 5 | 6 | 7 | 8 | 9 | 10 |  |  |  |
| Choroid-RPE | complex | 296 | 0 | 0 | 0 | 0 | 0 | 0 | 0 | 0 | 0 |  |
| Perivascular | cells | 4 | 0 | 0 | 0 | 0 | 0 | 1 | 0 | 4 | 5 |  |
| Macroglia | 0 | 0 | 3 | 0 | 0 | 1 | 0 | 0 | 0 | 0 |  |  |
| Microglia | 0 | 0 | 0 | 0 | 0 | 0 | 0 | 0 | 0 | 0 |  |  |
| Macrophages | 4 | 0 | 0 | 0 | 0 | 0 | 0 | 0 | 0 | 0 |  |  |
| Retinal | progenitor | cells | 0 | 0 | 0 | 1 | 0 | 0 | 0 | 4 | 0 | 0 |
| Photoreceptors | 1 | 0 | 1 | 1 | 2 | 5 | 0 | 314 | 0 | 0 |  |  |
| Interneurons | 0 | 0 | 174 | 0 | 0 | 5 | 0 | 1 | 0 | 0 |  |  |
| Ganglion | cells | 0 | 0 | 1 | 0 | 0 | 0 | 32 | 0 | 4 | 9 |  |
| Unspecific | cell | types | 0 | 0 | 5 | 1 | 0 | 4 | 0 | 6 | 0 | 0 |
|  |  |  |  |  |  |  |  |  |  |  |  |  |
| , | = | slide3_008 | |  |  |  |  |  |  |  |  |  |
|  |  |  |  |  |  |  |  |  |  |  |  |  |
|  |  |  |  |  |  |  |  |  |  |  |  |  |
| 1 | 2 | 3 | 4 | 5 | 6 | 7 | 8 | 9 | 10 |  |  |  |
| Choroid-RPE | complex | 297 | 0 | 0 | 1 | 0 | 0 | 0 | 3 | 0 | 0 |  |
| Perivascular | cells | 0 | 0 | 0 | 0 | 0 | 0 | 0 | 0 | 0 | 0 |  |
| Macroglia | 0 | 9 | 0 | 0 | 0 | 0 | 0 | 0 | 0 | 1 |  |  |
| Microglia | 1 | 0 | 2 | 0 | 0 | 0 | 0 | 0 | 0 | 0 |  |  |
| Macrophages | 5 | 0 | 0 | 0 | 0 | 0 | 0 | 0 | 0 | 0 |  |  |
| Retinal | progenitor | cells | 0 | 0 | 4 | 6 | 0 | 0 | 0 | 3 | 0 | 0 |
| Photoreceptors | 2 | 0 | 4 | 6 | 2 | 2 | 0 | 307 | 0 | 0 |  |  |
| Interneurons | 0 | 0 | 224 | 3 | 0 | 3 | 0 | 4 | 0 | 0 |  |  |
| Ganglion | cells | 0 | 1 | 5 | 0 | 0 | 0 | 25 | 0 | 0 | 5 |  |
| Unspecific | cell | types | 1 | 0 | 0 | 0 | 7 | 2 | 0 | 7 | 0 | 0 |
|  |  |  |  |  |  |  |  |  |  |  |  |  |
| , | = | slide3_009 | |  |  |  |  |  |  |  |  |  |
|  |  |  |  |  |  |  |  |  |  |  |  |  |
|  |  |  |  |  |  |  |  |  |  |  |  |  |
| 1 | 2 | 3 | 4 | 5 | 6 | 7 | 8 | 9 | 10 |  |  |  |
| Choroid-RPE | complex | 306 | 0 | 0 | 0 | 0 | 0 | 0 | 0 | 1 | 0 |  |
| Perivascular | cells | 0 | 0 | 0 | 0 | 0 | 0 | 2 | 0 | 0 | 0 |  |
| Macroglia | 0 | 0 | 4 | 0 | 0 | 3 | 3 | 0 | 0 | 1 |  |  |
| Microglia | 0 | 0 | 0 | 0 | 0 | 0 | 0 | 0 | 0 | 0 |  |  |
| Macrophages | 3 | 0 | 0 | 0 | 0 | 0 | 0 | 0 | 0 | 0 |  |  |
| Retinal | progenitor | cells | 0 | 0 | 0 | 0 | 0 | 0 | 1 | 0 | 0 | 0 |
| Photoreceptors | 0 | 0 | 0 | 0 | 1 | 1 | 0 | 393 | 0 | 0 |  |  |
| Interneurons | 0 | 0 | 170 | 0 | 1 | 14 | 0 | 3 | 0 | 0 |  |  |
| Ganglion | cells | 0 | 0 | 2 | 0 | 0 | 0 | 37 | 0 | 0 | 6 |  |
| Unspecific | cell | types | 0 | 0 | 4 | 0 | 1 | 6 | 0 | 4 | 0 | 0 |

| **Table S11. Expression neighborhoods** | | | | | | | | | | | | |
| --- | --- | --- | --- | --- | --- | --- | --- | --- | --- | --- | --- | --- |
| , | = | day1_002 |  |  |  |  |  |  |  |  |  |  |
|  |  |  |  |  |  |  |  |  |  |  |  |  |
|  |  |  |  |  |  |  |  |  |  |  |  |  |
| 1 | 2 | 3 | 4 | 5 | 6 | 7 | 8 | 9 | 10 |  |  |  |
| Choroid-RPE | complex | 73 | 54 | 0 | 0 | 0 | 0 | 0 | 0 | 0 | 22 |  |
| Perivascular | cells | 0 | 0 | 0 | 0 | 0 | 0 | 0 | 0 | 10 | 0 |  |
| Macroglia | 0 | 0 | 0 | 0 | 16 | 6 | 0 | 0 | 115 | 57 |  |  |
| Microglia | 0 | 1 | 0 | 0 | 0 | 1 | 0 | 0 | 2 | 0 |  |  |
| Macrophages | 1 | 0 | 0 | 0 | 0 | 0 | 0 | 0 | 0 | 0 |  |  |
| Retinal | progenitor | cells | 0 | 0 | 3 | 0 | 3 | 23 | 0 | 26 | 4 | 6 |
| Photoreceptors | 0 | 0 | 24 | 1 | 4 | 251 | 4 | 67 | 3 | 12 |  |  |
| Interneurons | 0 | 0 | 0 | 0 | 29 | 8 | 0 | 0 | 25 | 78 |  |  |
| Ganglion | cells | 0 | 0 | 0 | 0 | 0 | 0 | 0 | 0 | 5 | 2 |  |
| Unspecific | cell | types | 0 | 0 | 1 | 0 | 7 | 2 | 0 | 0 | 7 | 4 |
|  |  |  |  |  |  |  |  |  |  |  |  |  |
| , | = | day1_003 |  |  |  |  |  |  |  |  |  |  |
|  |  |  |  |  |  |  |  |  |  |  |  |  |
|  |  |  |  |  |  |  |  |  |  |  |  |  |
| 1 | 2 | 3 | 4 | 5 | 6 | 7 | 8 | 9 | 10 |  |  |  |
| Choroid-RPE | complex | 101 | 22 | 23 | 0 | 0 | 0 | 0 | 0 | 0 | 0 |  |
| Perivascular | cells | 0 | 2 | 0 | 0 | 0 | 0 | 0 | 0 | 7 | 0 |  |
| Macroglia | 0 | 2 | 0 | 0 | 33 | 1 | 0 | 1 | 127 | 2 |  |  |
| Microglia | 1 | 1 | 0 | 0 | 0 | 0 | 0 | 0 | 3 | 0 |  |  |
| Macrophages | 3 | 1 | 10 | 0 | 0 | 0 | 0 | 0 | 1 | 0 |  |  |
| Retinal | progenitor | cells | 0 | 0 | 0 | 5 | 14 | 1 | 3 | 145 | 4 | 0 |
| Photoreceptors | 0 | 0 | 0 | 70 | 19 | 9 | 13 | 164 | 1 | 0 |  |  |
| Interneurons | 0 | 0 | 0 | 0 | 34 | 3 | 0 | 0 | 22 | 0 |  |  |
| Ganglion | cells | 0 | 0 | 0 | 0 | 3 | 0 | 0 | 0 | 2 | 4 |  |
| Unspecific | cell | types | 27 | 34 | 16 | 0 | 3 | 0 | 0 | 0 | 9 | 1 |
|  |  |  |  |  |  |  |  |  |  |  |  |  |
| , | = | day1_004 |  |  |  |  |  |  |  |  |  |  |
|  |  |  |  |  |  |  |  |  |  |  |  |  |
|  |  |  |  |  |  |  |  |  |  |  |  |  |
| 1 | 2 | 3 | 4 | 5 | 6 | 7 | 8 | 9 | 10 |  |  |  |
| Choroid-RPE | complex | 0 | 0 | 0 | 0 | 0 | 0 | 0 | 0 | 0 | 65 |  |
| Perivascular | cells | 0 | 0 | 0 | 0 | 0 | 0 | 0 | 0 | 0 | 0 |  |
| Macroglia | 0 | 0 | 0 | 0 | 9 | 2 | 0 | 0 | 22 | 71 |  |  |
| Microglia | 0 | 0 | 0 | 0 | 1 | 10 | 4 | 0 | 0 | 29 |  |  |
| Macrophages | 0 | 0 | 0 | 0 | 0 | 0 | 0 | 0 | 0 | 0 |  |  |
| Retinal | progenitor | cells | 0 | 0 | 0 | 0 | 1 | 2 | 0 | 0 | 0 | 2 |
| Photoreceptors | 0 | 0 | 0 | 0 | 1 | 406 | 144 | 0 | 0 | 33 |  |  |
| Interneurons | 0 | 0 | 0 | 0 | 48 | 104 | 0 | 0 | 4 | 99 |  |  |
| Ganglion | cells | 0 | 0 | 0 | 0 | 0 | 0 | 0 | 0 | 0 | 60 |  |
| Unspecific | cell | types | 0 | 0 | 0 | 0 | 0 | 1 | 0 | 0 | 0 | 16 |
|  |  |  |  |  |  |  |  |  |  |  |  |  |
| , | = | day1_005 |  |  |  |  |  |  |  |  |  |  |
|  |  |  |  |  |  |  |  |  |  |  |  |  |
|  |  |  |  |  |  |  |  |  |  |  |  |  |
| 1 | 2 | 3 | 4 | 5 | 6 | 7 | 8 | 9 | 10 |  |  |  |
| Choroid-RPE | complex | 0 | 0 | 0 | 0 | 0 | 0 | 0 | 0 | 0 | 60 |  |
| Perivascular | cells | 0 | 0 | 0 | 0 | 0 | 0 | 0 | 0 | 0 | 0 |  |
| Macroglia | 0 | 0 | 0 | 0 | 28 | 2 | 0 | 0 | 17 | 49 |  |  |
| Microglia | 0 | 0 | 0 | 0 | 3 | 5 | 1 | 0 | 2 | 9 |  |  |
| Macrophages | 0 | 0 | 0 | 0 | 0 | 0 | 0 | 0 | 0 | 0 |  |  |
| Retinal | progenitor | cells | 0 | 0 | 0 | 0 | 5 | 2 | 1 | 5 | 1 | 0 |
| Photoreceptors | 0 | 0 | 0 | 20 | 6 | 317 | 245 | 7 | 1 | 14 |  |  |
| Interneurons | 0 | 0 | 0 | 0 | 87 | 143 | 1 | 0 | 4 | 34 |  |  |
| Ganglion | cells | 0 | 0 | 0 | 0 | 5 | 15 | 0 | 0 | 3 | 66 |  |
| Unspecific | cell | types | 0 | 0 | 0 | 0 | 2 | 0 | 1 | 0 | 2 | 28 |
|  |  |  |  |  |  |  |  |  |  |  |  |  |
| , | = | day1_006 |  |  |  |  |  |  |  |  |  |  |
|  |  |  |  |  |  |  |  |  |  |  |  |  |
|  |  |  |  |  |  |  |  |  |  |  |  |  |
| 1 | 2 | 3 | 4 | 5 | 6 | 7 | 8 | 9 | 10 |  |  |  |
| Choroid-RPE | complex | 4 | 55 | 29 | 4 | 1 | 12 | 3 | 0 | 0 | 0 |  |
| Perivascular | cells | 0 | 0 | 2 | 0 | 0 | 0 | 0 | 0 | 0 | 0 |  |
| Macroglia | 0 | 0 | 0 | 1 | 23 | 3 | 0 | 0 | 2 | 0 |  |  |
| Microglia | 0 | 0 | 0 | 0 | 0 | 0 | 1 | 0 | 0 | 0 |  |  |
| Macrophages | 1 | 0 | 7 | 0 | 0 | 2 | 0 | 0 | 0 | 0 |  |  |
| Retinal | progenitor | cells | 0 | 0 | 0 | 2 | 3 | 0 | 3 | 50 | 0 | 0 |
| Photoreceptors | 0 | 0 | 0 | 24 | 5 | 9 | 290 | 23 | 0 | 0 |  |  |
| Interneurons | 0 | 0 | 0 | 12 | 43 | 12 | 100 | 2 | 0 | 0 |  |  |
| Ganglion | cells | 0 | 0 | 0 | 0 | 18 | 30 | 1 | 0 | 0 | 8 |  |
| Unspecific | cell | types | 4 | 29 | 33 | 0 | 9 | 6 | 3 | 4 | 3 | 0 |
|  |  |  |  |  |  |  |  |  |  |  |  |  |
| , | = | day1_007 |  |  |  |  |  |  |  |  |  |  |
|  |  |  |  |  |  |  |  |  |  |  |  |  |
|  |  |  |  |  |  |  |  |  |  |  |  |  |
| 1 | 2 | 3 | 4 | 5 | 6 | 7 | 8 | 9 | 10 |  |  |  |
| Choroid-RPE | complex | 252 | 117 | 0 | 0 | 3 | 0 | 0 | 0 | 0 | 0 |  |
| Perivascular | cells | 1 | 1 | 0 | 0 | 3 | 0 | 0 | 0 | 0 | 0 |  |
| Macroglia | 0 | 0 | 0 | 1 | 4 | 0 | 4 | 1 | 0 | 0 |  |  |
| Microglia | 0 | 1 | 0 | 0 | 0 | 0 | 1 | 0 | 0 | 0 |  |  |
| Macrophages | 0 | 3 | 1 | 0 | 1 | 0 | 0 | 0 | 0 | 0 |  |  |
| Retinal | progenitor | cells | 0 | 0 | 0 | 1 | 1 | 1 | 2 | 24 | 0 | 0 |
| Photoreceptors | 0 | 0 | 0 | 77 | 2 | 0 | 226 | 11 | 0 | 0 |  |  |
| Interneurons | 0 | 0 | 0 | 27 | 37 | 1 | 101 | 16 | 0 | 0 |  |  |
| Ganglion | cells | 0 | 0 | 0 | 0 | 7 | 26 | 2 | 0 | 0 | 0 |  |
| Unspecific | cell | types | 0 | 0 | 0 | 1 | 1 | 0 | 2 | 1 | 0 | 0 |
|  |  |  |  |  |  |  |  |  |  |  |  |  |
| , | = | day1_008 |  |  |  |  |  |  |  |  |  |  |
|  |  |  |  |  |  |  |  |  |  |  |  |  |
|  |  |  |  |  |  |  |  |  |  |  |  |  |
| 1 | 2 | 3 | 4 | 5 | 6 | 7 | 8 | 9 | 10 |  |  |  |
| Choroid-RPE | complex | 102 | 143 | 73 | 0 | 0 | 0 | 0 | 0 | 0 | 0 |  |
| Perivascular | cells | 0 | 2 | 0 | 0 | 0 | 5 | 0 | 0 | 0 | 0 |  |
| Macroglia | 0 | 0 | 0 | 0 | 2 | 0 | 0 | 0 | 0 | 0 |  |  |
| Microglia | 0 | 1 | 0 | 0 | 0 | 1 | 0 | 0 | 0 | 0 |  |  |
| Macrophages | 0 | 1 | 0 | 0 | 0 | 0 | 0 | 0 | 0 | 0 |  |  |
| Retinal | progenitor | cells | 0 | 0 | 0 | 3 | 0 | 0 | 5 | 12 | 0 | 0 |
| Photoreceptors | 0 | 0 | 0 | 69 | 3 | 18 | 189 | 19 | 0 | 0 |  |  |
| Interneurons | 0 | 0 | 0 | 6 | 30 | 19 | 108 | 5 | 0 | 0 |  |  |
| Ganglion | cells | 0 | 0 | 0 | 0 | 6 | 32 | 0 | 0 | 0 | 0 |  |
| Unspecific | cell | types | 0 | 0 | 0 | 2 | 5 | 2 | 8 | 0 | 0 | 0 |
|  |  |  |  |  |  |  |  |  |  |  |  |  |
| , | = | day1_009 |  |  |  |  |  |  |  |  |  |  |
|  |  |  |  |  |  |  |  |  |  |  |  |  |
|  |  |  |  |  |  |  |  |  |  |  |  |  |
| 1 | 2 | 3 | 4 | 5 | 6 | 7 | 8 | 9 | 10 |  |  |  |
| Choroid-RPE | complex | 30 | 164 | 0 | 1 | 1 | 2 | 0 | 1 | 0 | 2 |  |
| Perivascular | cells | 0 | 1 | 0 | 0 | 0 | 0 | 0 | 0 | 0 | 0 |  |
| Macroglia | 0 | 0 | 0 | 0 | 1 | 1 | 1 | 0 | 0 | 0 |  |  |
| Microglia | 0 | 3 | 0 | 0 | 0 | 1 | 0 | 1 | 0 | 0 |  |  |
| Macrophages | 0 | 1 | 1 | 0 | 0 | 0 | 0 | 0 | 0 | 0 |  |  |
| Retinal | progenitor | cells | 0 | 0 | 0 | 0 | 1 | 2 | 3 | 25 | 0 | 0 |
| Photoreceptors | 0 | 1 | 0 | 2 | 13 | 186 | 95 | 24 | 0 | 0 |  |  |
| Interneurons | 0 | 0 | 15 | 0 | 15 | 92 | 0 | 0 | 0 | 0 |  |  |
| Ganglion | cells | 0 | 0 | 58 | 0 | 0 | 10 | 0 | 0 | 0 | 0 |  |
| Unspecific | cell | types | 33 | 43 | 2 | 0 | 0 | 0 | 0 | 0 | 0 | 0 |
|  |  |  |  |  |  |  |  |  |  |  |  |  |
| , | = | slide2_001 | |  |  |  |  |  |  |  |  |  |
|  |  |  |  |  |  |  |  |  |  |  |  |  |
|  |  |  |  |  |  |  |  |  |  |  |  |  |
| 1 | 2 | 3 | 4 | 5 | 6 | 7 | 8 | 9 | 10 |  |  |  |
| Choroid-RPE | complex | 85 | 25 | 76 | 0 | 0 | 0 | 0 | 1 | 0 | 0 |  |
| Perivascular | cells | 1 | 0 | 0 | 0 | 0 | 0 | 0 | 0 | 8 | 0 |  |
| Macroglia | 0 | 0 | 1 | 0 | 51 | 0 | 0 | 2 | 148 | 0 |  |  |
| Microglia | 0 | 0 | 0 | 0 | 0 | 0 | 0 | 0 | 2 | 0 |  |  |
| Macrophages | 2 | 2 | 8 | 0 | 0 | 0 | 0 | 0 | 0 | 0 |  |  |
| Retinal | progenitor | cells | 0 | 0 | 0 | 16 | 4 | 1 | 0 | 92 | 12 | 0 |
| Photoreceptors | 0 | 9 | 71 | 157 | 1 | 2 | 10 | 70 | 1 | 0 |  |  |
| Interneurons | 0 | 0 | 46 | 0 | 76 | 0 | 0 | 0 | 20 | 0 |  |  |
| Ganglion | cells | 0 | 0 | 0 | 0 | 0 | 0 | 0 | 0 | 12 | 0 |  |
| Unspecific | cell | types | 1 | 0 | 12 | 0 | 14 | 0 | 2 | 0 | 0 | 0 |
|  |  |  |  |  |  |  |  |  |  |  |  |  |
| , | = | slide2_002 | |  |  |  |  |  |  |  |  |  |
|  |  |  |  |  |  |  |  |  |  |  |  |  |
|  |  |  |  |  |  |  |  |  |  |  |  |  |
| 1 | 2 | 3 | 4 | 5 | 6 | 7 | 8 | 9 | 10 |  |  |  |
| Choroid-RPE | complex | 20 | 26 | 96 | 0 | 0 | 0 | 0 | 1 | 0 | 0 |  |
| Perivascular | cells | 10 | 4 | 0 | 0 | 0 | 0 | 0 | 0 | 1 | 0 |  |
| Macroglia | 4 | 3 | 2 | 0 | 14 | 0 | 0 | 1 | 165 | 0 |  |  |
| Microglia | 0 | 0 | 0 | 0 | 0 | 0 | 0 | 0 | 1 | 0 |  |  |
| Macrophages | 1 | 2 | 11 | 0 | 0 | 0 | 0 | 0 | 0 | 0 |  |  |
| Retinal | progenitor | cells | 0 | 0 | 2 | 1 | 3 | 3 | 1 | 165 | 11 | 0 |
| Photoreceptors | 0 | 0 | 14 | 7 | 0 | 8 | 8 | 84 | 2 | 0 |  |  |
| Interneurons | 0 | 1 | 0 | 0 | 8 | 0 | 0 | 0 | 9 | 0 |  |  |
| Ganglion | cells | 0 | 0 | 0 | 0 | 0 | 0 | 0 | 0 | 1 | 0 |  |
| Unspecific | cell | types | 169 | 49 | 64 | 14 | 2 | 0 | 6 | 3 | 1 | 0 |
|  |  |  |  |  |  |  |  |  |  |  |  |  |
| , | = | slide2_003 | |  |  |  |  |  |  |  |  |  |
|  |  |  |  |  |  |  |  |  |  |  |  |  |
|  |  |  |  |  |  |  |  |  |  |  |  |  |
| 1 | 2 | 3 | 4 | 5 | 6 | 7 | 8 | 9 | 10 |  |  |  |
| Choroid-RPE | complex | 0 | 1 | 0 | 0 | 1 | 3 | 0 | 1 | 0 | 47 |  |
| Perivascular | cells | 0 | 0 | 0 | 0 | 0 | 0 | 0 | 0 | 0 | 0 |  |
| Macroglia | 0 | 0 | 0 | 2 | 48 | 0 | 1 | 0 | 23 | 2 |  |  |
| Microglia | 0 | 0 | 0 | 0 | 4 | 0 | 0 | 0 | 5 | 4 |  |  |
| Macrophages | 0 | 0 | 0 | 0 | 0 | 0 | 0 | 0 | 0 | 0 |  |  |
| Retinal | progenitor | cells | 0 | 0 | 0 | 12 | 1 | 3 | 14 | 12 | 0 | 0 |
| Photoreceptors | 0 | 0 | 0 | 321 | 3 | 3 | 236 | 19 | 0 | 3 |  |  |
| Interneurons | 0 | 0 | 0 | 8 | 199 | 7 | 19 | 0 | 6 | 1 |  |  |
| Ganglion | cells | 0 | 5 | 0 | 0 | 11 | 0 | 0 | 0 | 40 | 11 |  |
| Unspecific | cell | types | 0 | 0 | 0 | 5 | 3 | 1 | 0 | 4 | 2 | 3 |
|  |  |  |  |  |  |  |  |  |  |  |  |  |
| , | = | slide2_004 | |  |  |  |  |  |  |  |  |  |
|  |  |  |  |  |  |  |  |  |  |  |  |  |
|  |  |  |  |  |  |  |  |  |  |  |  |  |
| 1 | 2 | 3 | 4 | 5 | 6 | 7 | 8 | 9 | 10 |  |  |  |
| Choroid-RPE | complex | 0 | 0 | 0 | 0 | 0 | 23 | 0 | 3 | 0 | 70 |  |
| Perivascular | cells | 0 | 0 | 0 | 0 | 2 | 0 | 0 | 0 | 5 | 0 |  |
| Macroglia | 0 | 0 | 0 | 1 | 21 | 0 | 0 | 0 | 74 | 2 |  |  |
| Microglia | 0 | 0 | 0 | 1 | 2 | 0 | 1 | 0 | 8 | 2 |  |  |
| Macrophages | 0 | 0 | 0 | 0 | 0 | 0 | 0 | 0 | 0 | 0 |  |  |
| Retinal | progenitor | cells | 0 | 0 | 0 | 46 | 10 | 0 | 1 | 35 | 1 | 1 |
| Photoreceptors | 0 | 0 | 0 | 497 | 2 | 0 | 185 | 25 | 0 | 0 |  |  |
| Interneurons | 0 | 0 | 0 | 79 | 109 | 5 | 97 | 2 | 7 | 11 |  |  |
| Ganglion | cells | 0 | 0 | 0 | 0 | 4 | 0 | 0 | 0 | 18 | 2 |  |
| Unspecific | cell | types | 0 | 0 | 0 | 0 | 0 | 0 | 0 | 0 | 1 | 4 |
|  |  |  |  |  |  |  |  |  |  |  |  |  |
| , | = | slide2_005 | |  |  |  |  |  |  |  |  |  |
|  |  |  |  |  |  |  |  |  |  |  |  |  |
|  |  |  |  |  |  |  |  |  |  |  |  |  |
| 1 | 2 | 3 | 4 | 5 | 6 | 7 | 8 | 9 | 10 |  |  |  |
| Choroid-RPE | complex | 125 | 69 | 91 | 0 | 1 | 7 | 0 | 2 | 0 | 3 |  |
| Perivascular | cells | 0 | 4 | 0 | 0 | 0 | 1 | 0 | 0 | 0 | 0 |  |
| Macroglia | 0 | 0 | 0 | 0 | 15 | 4 | 0 | 3 | 1 | 1 |  |  |
| Microglia | 0 | 0 | 0 | 0 | 0 | 2 | 0 | 5 | 0 | 0 |  |  |
| Macrophages | 0 | 0 | 2 | 0 | 0 | 0 | 0 | 0 | 0 | 0 |  |  |
| Retinal | progenitor | cells | 0 | 0 | 0 | 0 | 3 | 6 | 6 | 94 | 0 | 0 |
| Photoreceptors | 0 | 0 | 0 | 3 | 3 | 67 | 198 | 32 | 0 | 0 |  |  |
| Interneurons | 0 | 0 | 0 | 1 | 64 | 20 | 59 | 24 | 1 | 0 |  |  |
| Ganglion | cells | 0 | 0 | 0 | 0 | 7 | 26 | 0 | 0 | 4 | 26 |  |
| Unspecific | cell | types | 0 | 0 | 0 | 0 | 5 | 5 | 2 | 1 | 0 | 0 |
|  |  |  |  |  |  |  |  |  |  |  |  |  |
| , | = | slide2_006 | |  |  |  |  |  |  |  |  |  |
|  |  |  |  |  |  |  |  |  |  |  |  |  |
|  |  |  |  |  |  |  |  |  |  |  |  |  |
| 1 | 2 | 3 | 4 | 5 | 6 | 7 | 8 | 9 | 10 |  |  |  |
| Choroid-RPE | complex | 147 | 105 | 51 | 0 | 0 | 4 | 0 | 2 | 0 | 0 |  |
| Perivascular | cells | 0 | 1 | 0 | 0 | 0 | 0 | 0 | 0 | 0 | 3 |  |
| Macroglia | 0 | 0 | 0 | 0 | 11 | 6 | 0 | 4 | 2 | 7 |  |  |
| Microglia | 0 | 1 | 0 | 0 | 0 | 1 | 0 | 0 | 0 | 0 |  |  |
| Macrophages | 0 | 1 | 0 | 0 | 0 | 0 | 0 | 0 | 0 | 0 |  |  |
| Retinal | progenitor | cells | 0 | 0 | 0 | 0 | 3 | 10 | 0 | 55 | 3 | 0 |
| Photoreceptors | 0 | 0 | 0 | 0 | 14 | 245 | 28 | 20 | 0 | 5 |  |  |
| Interneurons | 0 | 0 | 0 | 0 | 38 | 142 | 0 | 15 | 5 | 14 |  |  |
| Ganglion | cells | 0 | 0 | 0 | 0 | 1 | 4 | 0 | 0 | 0 | 63 |  |
| Unspecific | cell | types | 0 | 0 | 0 | 0 | 1 | 1 | 0 | 2 | 0 | 1 |
|  |  |  |  |  |  |  |  |  |  |  |  |  |
| , | = | slide2_007 | |  |  |  |  |  |  |  |  |  |
|  |  |  |  |  |  |  |  |  |  |  |  |  |
|  |  |  |  |  |  |  |  |  |  |  |  |  |
| 1 | 2 | 3 | 4 | 5 | 6 | 7 | 8 | 9 | 10 |  |  |  |
| Choroid-RPE | complex | 55 | 66 | 60 | 0 | 0 | 6 | 3 | 1 | 0 | 2 |  |
| Perivascular | cells | 0 | 0 | 0 | 0 | 0 | 1 | 0 | 0 | 0 | 0 |  |
| Macroglia | 0 | 0 | 0 | 2 | 39 | 0 | 1 | 2 | 0 | 0 |  |  |
| Microglia | 0 | 1 | 0 | 0 | 0 | 0 | 0 | 7 | 0 | 0 |  |  |
| Macrophages | 0 | 2 | 1 | 0 | 0 | 0 | 0 | 0 | 0 | 0 |  |  |
| Retinal | progenitor | cells | 0 | 0 | 0 | 8 | 2 | 0 | 5 | 63 | 0 | 0 |
| Photoreceptors | 0 | 0 | 0 | 84 | 7 | 1 | 317 | 29 | 0 | 0 |  |  |
| Interneurons | 0 | 0 | 0 | 126 | 44 | 2 | 63 | 4 | 0 | 0 |  |  |
| Ganglion | cells | 0 | 0 | 0 | 0 | 27 | 32 | 0 | 1 | 0 | 0 |  |
| Unspecific | cell | types | 0 | 0 | 0 | 1 | 3 | 0 | 4 | 0 | 0 | 0 |
|  |  |  |  |  |  |  |  |  |  |  |  |  |
| , | = | slide2_008 | |  |  |  |  |  |  |  |  |  |
|  |  |  |  |  |  |  |  |  |  |  |  |  |
|  |  |  |  |  |  |  |  |  |  |  |  |  |
| 1 | 2 | 3 | 4 | 5 | 6 | 7 | 8 | 9 | 10 |  |  |  |
| Choroid-RPE | complex | 35 | 160 | 2 | 1 | 0 | 0 | 2 | 0 | 0 | 0 |  |
| Perivascular | cells | 0 | 1 | 0 | 0 | 14 | 9 | 14 | 0 | 0 | 0 |  |
| Macroglia | 0 | 0 | 0 | 0 | 17 | 1 | 0 | 0 | 1 | 0 |  |  |
| Microglia | 0 | 1 | 0 | 0 | 0 | 0 | 0 | 0 | 0 | 0 |  |  |
| Macrophages | 0 | 3 | 1 | 0 | 3 | 2 | 2 | 0 | 0 | 0 |  |  |
| Retinal | progenitor | cells | 0 | 1 | 0 | 4 | 4 | 2 | 2 | 47 | 0 | 0 |
| Photoreceptors | 0 | 7 | 5 | 12 | 8 | 13 | 117 | 15 | 0 | 0 |  |  |
| Interneurons | 0 | 5 | 0 | 36 | 25 | 8 | 45 | 13 | 0 | 0 |  |  |
| Ganglion | cells | 0 | 0 | 0 | 0 | 2 | 37 | 1 | 0 | 0 | 2 |  |
| Unspecific | cell | types | 7 | 41 | 48 | 94 | 41 | 12 | 67 | 1 | 0 | 1 |
|  |  |  |  |  |  |  |  |  |  |  |  |  |
| , | = | slide2_009 | |  |  |  |  |  |  |  |  |  |
|  |  |  |  |  |  |  |  |  |  |  |  |  |
|  |  |  |  |  |  |  |  |  |  |  |  |  |
| 1 | 2 | 3 | 4 | 5 | 6 | 7 | 8 | 9 | 10 |  |  |  |
| Choroid-RPE | complex | 189 | 90 | 70 | 0 | 0 | 6 | 0 | 0 | 0 | 8 |  |
| Perivascular | cells | 0 | 0 | 0 | 0 | 0 | 0 | 0 | 0 | 0 | 0 |  |
| Macroglia | 0 | 0 | 0 | 0 | 20 | 1 | 0 | 1 | 1 | 1 |  |  |
| Microglia | 0 | 2 | 0 | 0 | 0 | 0 | 0 | 1 | 1 | 0 |  |  |
| Macrophages | 0 | 2 | 0 | 0 | 0 | 0 | 0 | 0 | 0 | 0 |  |  |
| Retinal | progenitor | cells | 0 | 0 | 0 | 0 | 13 | 5 | 2 | 20 | 0 | 0 |
| Photoreceptors | 0 | 0 | 0 | 1 | 21 | 92 | 104 | 14 | 0 | 2 |  |  |
| Interneurons | 0 | 0 | 0 | 0 | 126 | 59 | 0 | 4 | 0 | 1 |  |  |
| Ganglion | cells | 0 | 0 | 0 | 0 | 2 | 29 | 0 | 0 | 0 | 18 |  |
| Unspecific | cell | types | 0 | 2 | 0 | 0 | 2 | 2 | 0 | 0 | 0 | 0 |
|  |  |  |  |  |  |  |  |  |  |  |  |  |
| , | = | slide2_010 | |  |  |  |  |  |  |  |  |  |
|  |  |  |  |  |  |  |  |  |  |  |  |  |
|  |  |  |  |  |  |  |  |  |  |  |  |  |
| 1 | 2 | 3 | 4 | 5 | 6 | 7 | 8 | 9 | 10 |  |  |  |
| Choroid-RPE | complex | 127 | 92 | 2 | 0 | 0 | 0 | 0 | 0 | 5 | 1 |  |
| Perivascular | cells | 0 | 0 | 0 | 0 | 0 | 0 | 0 | 0 | 0 | 1 |  |
| Macroglia | 0 | 3 | 0 | 0 | 20 | 0 | 0 | 0 | 1 | 4 |  |  |
| Microglia | 0 | 8 | 0 | 0 | 0 | 0 | 0 | 1 | 0 | 0 |  |  |
| Macrophages | 0 | 0 | 0 | 0 | 0 | 0 | 0 | 0 | 0 | 0 |  |  |
| Retinal | progenitor | cells | 0 | 0 | 0 | 0 | 17 | 4 | 1 | 32 | 0 | 0 |
| Photoreceptors | 0 | 0 | 0 | 0 | 43 | 194 | 101 | 23 | 0 | 0 |  |  |
| Interneurons | 0 | 0 | 0 | 0 | 103 | 83 | 0 | 0 | 0 | 2 |  |  |
| Ganglion | cells | 0 | 0 | 0 | 0 | 2 | 1 | 0 | 0 | 0 | 41 |  |
| Unspecific | cell | types | 0 | 3 | 0 | 0 | 7 | 2 | 0 | 1 | 0 | 0 |
|  |  |  |  |  |  |  |  |  |  |  |  |  |
| , | = | slide3_001 | |  |  |  |  |  |  |  |  |  |
|  |  |  |  |  |  |  |  |  |  |  |  |  |
|  |  |  |  |  |  |  |  |  |  |  |  |  |
| 1 | 2 | 3 | 4 | 5 | 6 | 7 | 8 | 9 | 10 |  |  |  |
| Choroid-RPE | complex | 79 | 13 | 140 | 0 | 0 | 0 | 0 | 0 | 0 | 0 |  |
| Perivascular | cells | 1 | 1 | 0 | 0 | 0 | 0 | 0 | 0 | 21 | 0 |  |
| Macroglia | 0 | 0 | 0 | 0 | 62 | 1 | 0 | 0 | 168 | 0 |  |  |
| Microglia | 0 | 0 | 0 | 0 | 2 | 0 | 0 | 1 | 2 | 0 |  |  |
| Macrophages | 1 | 5 | 5 | 0 | 0 | 0 | 0 | 0 | 0 | 0 |  |  |
| Retinal | progenitor | cells | 0 | 1 | 2 | 20 | 6 | 0 | 1 | 75 | 8 | 0 |
| Photoreceptors | 0 | 2 | 24 | 205 | 2 | 1 | 25 | 34 | 0 | 0 |  |  |
| Interneurons | 0 | 2 | 24 | 3 | 79 | 3 | 0 | 2 | 25 | 0 |  |  |
| Ganglion | cells | 0 | 0 | 0 | 0 | 0 | 0 | 0 | 0 | 8 | 0 |  |
| Unspecific | cell | types | 0 | 1 | 5 | 2 | 23 | 2 | 2 | 0 | 0 | 0 |
|  |  |  |  |  |  |  |  |  |  |  |  |  |
| , | = | slide3_002 | |  |  |  |  |  |  |  |  |  |
|  |  |  |  |  |  |  |  |  |  |  |  |  |
|  |  |  |  |  |  |  |  |  |  |  |  |  |
| 1 | 2 | 3 | 4 | 5 | 6 | 7 | 8 | 9 | 10 |  |  |  |
| Choroid-RPE | complex | 69 | 35 | 62 | 0 | 0 | 0 | 0 | 1 | 0 | 0 |  |
| Perivascular | cells | 1 | 0 | 0 | 0 | 0 | 0 | 0 | 0 | 20 | 0 |  |
| Macroglia | 0 | 0 | 0 | 0 | 42 | 0 | 0 | 1 | 108 | 0 |  |  |
| Microglia | 0 | 0 | 0 | 0 | 0 | 0 | 0 | 2 | 0 | 0 |  |  |
| Macrophages | 0 | 1 | 17 | 1 | 0 | 0 | 0 | 0 | 4 | 0 |  |  |
| Retinal | progenitor | cells | 0 | 0 | 0 | 6 | 1 | 0 | 1 | 63 | 7 | 0 |
| Photoreceptors | 0 | 3 | 111 | 98 | 4 | 0 | 30 | 35 | 0 | 0 |  |  |
| Interneurons | 0 | 0 | 40 | 0 | 84 | 1 | 0 | 0 | 17 | 0 |  |  |
| Ganglion | cells | 0 | 0 | 4 | 0 | 5 | 1 | 0 | 0 | 10 | 1 |  |
| Unspecific | cell | types | 0 | 2 | 5 | 0 | 6 | 1 | 0 | 0 | 1 | 0 |
|  |  |  |  |  |  |  |  |  |  |  |  |  |
| , | = | slide3_003 | |  |  |  |  |  |  |  |  |  |
|  |  |  |  |  |  |  |  |  |  |  |  |  |
|  |  |  |  |  |  |  |  |  |  |  |  |  |
| 1 | 2 | 3 | 4 | 5 | 6 | 7 | 8 | 9 | 10 |  |  |  |
| Choroid-RPE | complex | 0 | 19 | 0 | 0 | 1 | 19 | 0 | 2 | 0 | 42 |  |
| Perivascular | cells | 0 | 0 | 0 | 0 | 0 | 0 | 0 | 0 | 0 | 1 |  |
| Macroglia | 0 | 0 | 0 | 1 | 38 | 1 | 2 | 0 | 6 | 4 |  |  |
| Microglia | 0 | 0 | 0 | 0 | 2 | 0 | 0 | 0 | 0 | 0 |  |  |
| Macrophages | 0 | 0 | 0 | 0 | 0 | 0 | 0 | 0 | 0 | 0 |  |  |
| Retinal | progenitor | cells | 0 | 0 | 0 | 9 | 1 | 1 | 5 | 2 | 0 | 0 |
| Photoreceptors | 0 | 0 | 0 | 413 | 10 | 6 | 287 | 1 | 0 | 0 |  |  |
| Interneurons | 0 | 0 | 0 | 6 | 127 | 66 | 95 | 0 | 0 | 1 |  |  |
| Ganglion | cells | 0 | 0 | 0 | 0 | 19 | 17 | 0 | 0 | 0 | 37 |  |
| Unspecific | cell | types | 0 | 0 | 0 | 0 | 0 | 0 | 0 | 0 | 0 | 0 |
|  |  |  |  |  |  |  |  |  |  |  |  |  |
| , | = | slide3_004 | |  |  |  |  |  |  |  |  |  |
|  |  |  |  |  |  |  |  |  |  |  |  |  |
|  |  |  |  |  |  |  |  |  |  |  |  |  |
| 1 | 2 | 3 | 4 | 5 | 6 | 7 | 8 | 9 | 10 |  |  |  |
| Choroid-RPE | complex | 0 | 2 | 0 | 0 | 0 | 100 | 0 | 4 | 0 | 21 |  |
| Perivascular | cells | 0 | 0 | 0 | 0 | 6 | 0 | 0 | 0 | 0 | 0 |  |
| Macroglia | 0 | 0 | 0 | 0 | 50 | 0 | 0 | 0 | 15 | 1 |  |  |
| Microglia | 0 | 0 | 0 | 0 | 3 | 1 | 0 | 0 | 0 | 0 |  |  |
| Macrophages | 0 | 0 | 0 | 0 | 0 | 0 | 0 | 0 | 0 | 0 |  |  |
| Retinal | progenitor | cells | 0 | 0 | 0 | 27 | 2 | 0 | 0 | 19 | 0 | 0 |
| Photoreceptors | 0 | 0 | 0 | 461 | 4 | 10 | 329 | 18 | 0 | 0 |  |  |
| Interneurons | 0 | 0 | 0 | 60 | 89 | 12 | 178 | 0 | 1 | 2 |  |  |
| Ganglion | cells | 0 | 0 | 0 | 0 | 40 | 1 | 0 | 0 | 7 | 3 |  |
| Unspecific | cell | types | 0 | 0 | 0 | 0 | 3 | 0 | 0 | 0 | 0 | 0 |
|  |  |  |  |  |  |  |  |  |  |  |  |  |
| , | = | slide3_005 | |  |  |  |  |  |  |  |  |  |
|  |  |  |  |  |  |  |  |  |  |  |  |  |
|  |  |  |  |  |  |  |  |  |  |  |  |  |
| 1 | 2 | 3 | 4 | 5 | 6 | 7 | 8 | 9 | 10 |  |  |  |
| Choroid-RPE | complex | 71 | 102 | 89 | 0 | 5 | 0 | 0 | 0 | 0 | 0 |  |
| Perivascular | cells | 0 | 1 | 0 | 0 | 0 | 2 | 0 | 0 | 0 | 0 |  |
| Macroglia | 0 | 0 | 0 | 1 | 12 | 2 | 0 | 0 | 0 | 0 |  |  |
| Microglia | 2 | 6 | 0 | 0 | 1 | 1 | 0 | 2 | 0 | 0 |  |  |
| Macrophages | 0 | 0 | 6 | 0 | 0 | 0 | 0 | 0 | 0 | 0 |  |  |
| Retinal | progenitor | cells | 0 | 0 | 0 | 3 | 2 | 2 | 6 | 47 | 0 | 0 |
| Photoreceptors | 0 | 3 | 0 | 46 | 2 | 10 | 176 | 10 | 0 | 0 |  |  |
| Interneurons | 0 | 0 | 0 | 7 | 40 | 0 | 110 | 7 | 0 | 0 |  |  |
| Ganglion | cells | 0 | 0 | 0 | 1 | 12 | 58 | 15 | 2 | 1 | 0 |  |
| Unspecific | cell | types | 0 | 0 | 0 | 1 | 3 | 0 | 3 | 1 | 0 | 0 |
|  |  |  |  |  |  |  |  |  |  |  |  |  |
| , | = | slide3_006 | |  |  |  |  |  |  |  |  |  |
|  |  |  |  |  |  |  |  |  |  |  |  |  |
|  |  |  |  |  |  |  |  |  |  |  |  |  |
| 1 | 2 | 3 | 4 | 5 | 6 | 7 | 8 | 9 | 10 |  |  |  |
| Choroid-RPE | complex | 71 | 226 | 1 | 0 | 0 | 0 | 0 | 0 | 0 | 0 |  |
| Perivascular | cells | 0 | 0 | 3 | 0 | 0 | 0 | 0 | 0 | 0 | 0 |  |
| Macroglia | 0 | 0 | 0 | 0 | 1 | 0 | 1 | 0 | 0 | 0 |  |  |
| Microglia | 0 | 0 | 0 | 0 | 0 | 1 | 1 | 1 | 0 | 0 |  |  |
| Macrophages | 1 | 1 | 3 | 0 | 0 | 0 | 0 | 0 | 0 | 0 |  |  |
| Retinal | progenitor | cells | 0 | 0 | 0 | 0 | 0 | 1 | 2 | 18 | 0 | 0 |
| Photoreceptors | 0 | 1 | 24 | 23 | 11 | 1 | 162 | 16 | 0 | 0 |  |  |
| Interneurons | 0 | 1 | 11 | 24 | 14 | 2 | 91 | 8 | 0 | 0 |  |  |
| Ganglion | cells | 0 | 0 | 8 | 0 | 0 | 25 | 3 | 0 | 0 | 0 |  |
| Unspecific | cell | types | 10 | 35 | 41 | 1 | 1 | 0 | 1 | 3 | 0 | 0 |
|  |  |  |  |  |  |  |  |  |  |  |  |  |
| , | = | slide3_007 | |  |  |  |  |  |  |  |  |  |
|  |  |  |  |  |  |  |  |  |  |  |  |  |
|  |  |  |  |  |  |  |  |  |  |  |  |  |
| 1 | 2 | 3 | 4 | 5 | 6 | 7 | 8 | 9 | 10 |  |  |  |
| Choroid-RPE | complex | 106 | 93 | 80 | 0 | 12 | 4 | 1 | 0 | 0 | 0 |  |
| Perivascular | cells | 1 | 1 | 2 | 0 | 8 | 2 | 0 | 0 | 0 | 0 |  |
| Macroglia | 0 | 0 | 0 | 0 | 1 | 0 | 3 | 0 | 0 | 0 |  |  |
| Microglia | 0 | 0 | 0 | 0 | 0 | 0 | 0 | 0 | 0 | 0 |  |  |
| Macrophages | 0 | 0 | 4 | 0 | 0 | 0 | 0 | 0 | 0 | 0 |  |  |
| Retinal | progenitor | cells | 0 | 0 | 0 | 4 | 0 | 0 | 1 | 0 | 0 | 0 |
| Photoreceptors | 0 | 1 | 0 | 98 | 3 | 2 | 219 | 1 | 0 | 0 |  |  |
| Interneurons | 0 | 0 | 0 | 49 | 9 | 1 | 121 | 0 | 0 | 0 |  |  |
| Ganglion | cells | 0 | 0 | 0 | 0 | 15 | 25 | 6 | 0 | 0 | 0 |  |
| Unspecific | cell | types | 0 | 0 | 0 | 4 | 3 | 0 | 9 | 0 | 0 | 0 |
|  |  |  |  |  |  |  |  |  |  |  |  |  |
| , | = | slide3_008 | |  |  |  |  |  |  |  |  |  |
|  |  |  |  |  |  |  |  |  |  |  |  |  |
|  |  |  |  |  |  |  |  |  |  |  |  |  |
| 1 | 2 | 3 | 4 | 5 | 6 | 7 | 8 | 9 | 10 |  |  |  |
| Choroid-RPE | complex | 29 | 124 | 131 | 3 | 3 | 3 | 8 | 0 | 0 | 0 |  |
| Perivascular | cells | 0 | 0 | 0 | 0 | 0 | 0 | 0 | 0 | 0 | 0 |  |
| Macroglia | 0 | 0 | 0 | 0 | 0 | 0 | 0 | 0 | 10 | 0 |  |  |
| Microglia | 0 | 1 | 0 | 0 | 0 | 0 | 2 | 0 | 0 | 0 |  |  |
| Macrophages | 0 | 0 | 5 | 0 | 0 | 0 | 0 | 0 | 0 | 0 |  |  |
| Retinal | progenitor | cells | 0 | 0 | 0 | 6 | 0 | 1 | 1 | 5 | 0 | 0 |
| Photoreceptors | 0 | 0 | 95 | 60 | 0 | 10 | 155 | 3 | 0 | 0 |  |  |
| Interneurons | 0 | 0 | 4 | 51 | 6 | 1 | 171 | 1 | 0 | 0 |  |  |
| Ganglion | cells | 0 | 0 | 0 | 0 | 7 | 15 | 10 | 0 | 3 | 1 |  |
| Unspecific | cell | types | 0 | 0 | 1 | 6 | 0 | 0 | 9 | 0 | 0 | 1 |
|  |  |  |  |  |  |  |  |  |  |  |  |  |
| , | = | slide3_009 | |  |  |  |  |  |  |  |  |  |
|  |  |  |  |  |  |  |  |  |  |  |  |  |
|  |  |  |  |  |  |  |  |  |  |  |  |  |
| 1 | 2 | 3 | 4 | 5 | 6 | 7 | 8 | 9 | 10 |  |  |  |
| Choroid-RPE | complex | 144 | 64 | 99 | 0 | 0 | 0 | 0 | 0 | 0 | 0 |  |
| Perivascular | cells | 0 | 0 | 0 | 0 | 1 | 1 | 0 | 0 | 0 | 0 |  |
| Macroglia | 0 | 0 | 0 | 0 | 8 | 0 | 3 | 0 | 0 | 0 |  |  |
| Microglia | 0 | 0 | 0 | 0 | 0 | 0 | 0 | 0 | 0 | 0 |  |  |
| Macrophages | 0 | 0 | 3 | 0 | 0 | 0 | 0 | 0 | 0 | 0 |  |  |
| Retinal | progenitor | cells | 0 | 0 | 0 | 0 | 0 | 1 | 0 | 0 | 0 | 0 |
| Photoreceptors | 0 | 0 | 0 | 20 | 4 | 72 | 299 | 0 | 0 | 0 |  |  |
| Interneurons | 0 | 0 | 0 | 1 | 41 | 15 | 131 | 0 | 0 | 0 |  |  |
| Ganglion | cells | 0 | 0 | 0 | 0 | 13 | 26 | 5 | 0 | 0 | 1 |  |
| Unspecific | cell | types | 0 | 0 | 0 | 1 | 4 | 1 | 9 | 0 | 0 | 0 |

**Table S12. IMC differential state (C vs LC)**

| cluster_id | marker_id | ID | logFC | AveExpr | t | *p*_val | p_adj | B |
| --- | --- | --- | --- | --- | --- | --- | --- | --- |
| Choroid-RPE complex | αSMA | Choroid-RPE complex | -0.7721 | 0.7295 | -3.4752 | 0.0019 | 0.0137 | -2.1297 |
| Perivascular cells | αSMA | Perivascular cells | -24.7559 | 16.5474 | -3.4968 | 0.0024 | 0.0152 | -1.4451 |
| Ganglion cells | αSMA | Ganglion cells | -0.0712 | 0.1360 | -3.7525 | 0.0010 | 0.0078 | -1.2848 |
| Unspecific cell types | αSMA | Unspecific cell types | -0.9248 | 0.3142 | -3.1279 | 0.0045 | 0.0245 | -2.4393 |
| Retinal progenitor cells | pTyrosine | Retinal progenitor cells | 0.0437 | 0.1286 | 3.3999 | 0.0023 | 0.0152 | -2.0129 |
| Choroid-RPE complex | Vimentin | Choroid-RPE complex | -1.3250 | 0.8316 | -5.4810 | 0.0000 | 0.0002 | 2.8859 |
| Perivascular cells | Vimentin | Perivascular cells | -7.2756 | 5.9541 | -3.0470 | 0.0066 | 0.0323 | -2.2486 |
| Microglia | Vimentin | Microglia | -13.0953 | 5.0747 | -5.0916 | 0.0000 | 0.0005 | 1.9244 |
| Photoreceptors | Vimentin | Photoreceptors | -0.2406 | 0.2770 | -3.8511 | 0.0008 | 0.0063 | -1.8259 |
| Interneurons | Vimentin | Interneurons | -3.0464 | 1.6610 | -8.4720 | 0.0000 | 0.0000 | 9.6341 |
| Ganglion cells | Vimentin | Ganglion cells | -6.6311 | 2.9468 | -14.7586 | 0.0000 | 0.0000 | 21.1111 |
| Microglia | Ki67 | Microglia | -0.1996 | 0.1863 | -2.8132 | 0.0101 | 0.0435 | -2.6134 |
| Photoreceptors | Ki67 | Photoreceptors | -0.0184 | 0.0186 | -2.7534 | 0.0110 | 0.0458 | -4.4087 |
| Interneurons | Ki67 | Interneurons | -0.0571 | 0.0544 | -6.5626 | 0.0000 | 0.0000 | 5.1924 |
| Ganglion cells | Ki67 | Ganglion cells | -0.1214 | 0.0838 | -7.0447 | 0.0000 | 0.0000 | 6.8391 |
| Choroid-RPE complex | CD45 | Choroid-RPE complex | -0.3817 | 0.3496 | -2.8587 | 0.0086 | 0.0394 | -3.5532 |
| Microglia | CD45 | Microglia | -0.3456 | 0.3363 | -3.0276 | 0.0061 | 0.0309 | -2.2058 |
| Choroid-RPE complex | Fibronectin | Choroid-RPE complex | -0.9917 | 1.0011 | -3.2872 | 0.0031 | 0.0175 | -2.5755 |
| Photoreceptors | CD44 | Photoreceptors | -0.4823 | 1.0940 | -3.9908 | 0.0005 | 0.0048 | -1.4780 |
| Photoreceptors | beta_actin | Photoreceptors | -0.0262 | 0.0214 | -5.1483 | 0.0000 | 0.0004 | 1.4607 |
| Interneurons | beta_actin | Interneurons | -0.0253 | 0.0426 | -4.8926 | 0.0001 | 0.0006 | 1.0006 |
| Ganglion cells | beta_actin | Ganglion cells | -0.0424 | 0.0639 | -3.1905 | 0.0039 | 0.0216 | -2.6227 |
| Unspecific cell types | beta_actin | Unspecific cell types | -0.2800 | 0.0991 | -2.7107 | 0.0122 | 0.0486 | -3.3547 |
| Retinal progenitor cells | GranzymeB | Retinal progenitor cells | 0.0237 | 0.0507 | 4.0266 | 0.0005 | 0.0048 | -0.4972 |
| Choroid-RPE complex | F480 | Choroid-RPE complex | -0.8578 | 0.7971 | -3.0155 | 0.0060 | 0.0306 | -3.2025 |
| Retinal progenitor cells | Ecadherin | Retinal progenitor cells | -0.0811 | 0.1936 | -3.0772 | 0.0051 | 0.0270 | -2.7602 |
| Photoreceptors | Ecadherin | Photoreceptors | -0.0817 | 0.1581 | -4.1992 | 0.0003 | 0.0035 | -0.9548 |
| Interneurons | Ecadherin | Interneurons | -0.3035 | 0.4527 | -4.1912 | 0.0003 | 0.0035 | -0.7800 |
| Ganglion cells | Ecadherin | Ganglion cells | -0.8794 | 0.6962 | -8.4180 | 0.0000 | 0.0000 | 9.9350 |
| Unspecific cell types | Ecadherin | Unspecific cell types | -1.5801 | 0.4608 | -3.9939 | 0.0005 | 0.0048 | -0.3929 |
| Retinal progenitor cells | CD4 | Retinal progenitor cells | 0.0194 | 0.0310 | 2.7732 | 0.0105 | 0.0446 | -3.4341 |
| Choroid-RPE complex | iNOS_CD206 | Choroid-RPE complex | -2.0438 | 1.9107 | -2.8602 | 0.0086 | 0.0394 | -3.5500 |
| Unspecific cell types | iNOS_CD206 | Unspecific cell types | -1.9635 | 0.4541 | -2.7425 | 0.0113 | 0.0460 | -3.2872 |
| Choroid-RPE complex | CD11b | Choroid-RPE complex | -0.6905 | 0.6496 | -2.8520 | 0.0088 | 0.0394 | -3.5680 |
| Retinal progenitor cells | Arginase_perk | Retinal progenitor cells | -0.2326 | 0.6697 | -3.9238 | 0.0006 | 0.0055 | -0.7500 |
| Choroid-RPE complex | Beta_catenin | Choroid-RPE complex | -1.9574 | 1.4666 | -3.4691 | 0.0020 | 0.0137 | -2.1444 |
| Perivascular cells | Beta_catenin | Perivascular cells | -0.9926 | 0.7428 | -2.8890 | 0.0093 | 0.0412 | -2.5256 |
| Microglia | Beta_catenin | Microglia | -2.7502 | 1.7946 | -6.7700 | 0.0000 | 0.0000 | 5.0203 |
| Retinal progenitor cells | Beta_catenin | Retinal progenitor cells | -0.5760 | 1.5526 | -3.4412 | 0.0021 | 0.0143 | -1.9154 |
| Photoreceptors | Beta_catenin | Photoreceptors | -0.4878 | 0.5368 | -9.2502 | 0.0000 | 0.0000 | 11.1404 |
| Interneurons | Beta_catenin | Interneurons | -0.4923 | 0.5485 | -6.3343 | 0.0000 | 0.0000 | 4.6321 |
| Ganglion cells | Beta_catenin | Ganglion cells | -0.5340 | 0.5507 | -5.2663 | 0.0000 | 0.0003 | 2.4999 |
| Choroid-RPE complex | CollagenT1 | Choroid-RPE complex | -24.6444 | 7.6230 | -4.1461 | 0.0004 | 0.0037 | -0.4833 |
| Retinal progenitor cells | CollagenT1 | Retinal progenitor cells | -0.4902 | 0.3086 | -2.9556 | 0.0069 | 0.0329 | -3.0338 |
| Photoreceptors | CollagenT1 | Photoreceptors | -0.7528 | 0.4287 | -6.4665 | 0.0000 | 0.0000 | 4.7720 |
| Interneurons | CollagenT1 | Interneurons | -0.3324 | 0.2718 | -5.7201 | 0.0000 | 0.0001 | 3.1008 |
| Choroid-RPE complex | Pan_cytokeratin | Choroid-RPE complex | -2.1041 | 1.0688 | -9.1687 | 0.0000 | 0.0000 | 11.4367 |
| Photoreceptors | Pan_cytokeratin | Photoreceptors | -0.0726 | 0.0400 | -6.9801 | 0.0000 | 0.0000 | 6.0195 |
| Interneurons | Pan_cytokeratin | Interneurons | -0.0342 | 0.0435 | -3.3179 | 0.0029 | 0.0167 | -2.9237 |
| Choroid-RPE complex | B220 | Choroid-RPE complex | -0.5450 | 0.3382 | -6.6851 | 0.0000 | 0.0000 | 5.8552 |
| Choroid-RPE complex | DNA1 | Choroid-RPE complex | -52.0343 | 47.2815 | -3.6846 | 0.0012 | 0.0089 | -1.6239 |
| Unspecific cell types | DNA1 | Unspecific cell types | -36.8553 | 35.4673 | -3.3583 | 0.0026 | 0.0160 | -1.9103 |
| Choroid-RPE complex | DNA2 | Choroid-RPE complex | -95.5576 | 85.9798 | -3.6640 | 0.0012 | 0.0090 | -1.6741 |
| Unspecific cell types | DNA2 | Unspecific cell types | -68.3428 | 65.0935 | -3.3402 | 0.0027 | 0.0163 | -1.9524 |

**IMC differential state (C vs Fas)**

| cluster_id | marker_id | ID | logFC | AveExpr | t | *p*_val | p_adj | B |
| --- | --- | --- | --- | --- | --- | --- | --- | --- |
| Choroid-RPE complex | αSMA | Choroid-RPE complex | -0.9964 | 0.7295 | -4.8941 | 0.0001 | 0.0013 | 0.9916 |
| Photoreceptors | αSMA | Photoreceptors | -0.1929 | 0.2596 | -4.1307 | 0.0004 | 0.0055 | -1.6043 |
| Interneurons | αSMA | Interneurons | -0.0938 | 0.1770 | -3.9102 | 0.0007 | 0.0074 | -1.8815 |
| Choroid-RPE complex | pTyrosine | Choroid-RPE complex | -2.0072 | 1.4739 | -3.7872 | 0.0009 | 0.0077 | -1.8011 |
| Microglia | pTyrosine | Microglia | -0.4158 | 0.5019 | -2.9809 | 0.0069 | 0.0296 | -2.5561 |
| Retinal progenitor cells | pTyrosine | Retinal progenitor cells | 0.0520 | 0.1286 | 3.8167 | 0.0008 | 0.0075 | -1.2832 |
| Photoreceptors | Vimentin | Photoreceptors | 0.1905 | 0.2770 | 3.4352 | 0.0021 | 0.0139 | -3.3211 |
| Ganglion cells | Vimentin | Ganglion cells | 1.5535 | 2.9468 | 3.5870 | 0.0015 | 0.0106 | -2.0575 |
| Unspecific cell types | Vimentin | Unspecific cell types | 3.0608 | 2.4156 | 3.5942 | 0.0014 | 0.0106 | -1.6569 |
| Choroid-RPE complex | Ki67 | Choroid-RPE complex | -0.6136 | 0.4485 | -3.6553 | 0.0012 | 0.0099 | -2.1250 |
| Choroid-RPE complex | CD45 | Choroid-RPE complex | -0.4683 | 0.3496 | -3.8269 | 0.0008 | 0.0075 | -1.7030 |
| Retinal progenitor cells | CD45 | Retinal progenitor cells | -0.0314 | 0.0638 | -3.9973 | 0.0005 | 0.0067 | -0.8369 |
| Choroid-RPE complex | Fibronectin | Choroid-RPE complex | -1.5209 | 1.0011 | -5.5009 | 0.0000 | 0.0004 | 2.5347 |
| Retinal progenitor cells | Fibronectin | Retinal progenitor cells | -0.0610 | 0.0478 | -3.4367 | 0.0021 | 0.0139 | -2.2040 |
| Photoreceptors | Fibronectin | Photoreceptors | -0.0750 | 0.0362 | -8.2993 | 0.0000 | 0.0000 | 8.6254 |
| Choroid-RPE complex | CD44 | Choroid-RPE complex | -2.9228 | 1.8568 | -3.5743 | 0.0015 | 0.0106 | -2.3221 |
| Microglia | CD44 | Microglia | -5.6832 | 3.2079 | -3.2665 | 0.0035 | 0.0162 | -1.9384 |
| Retinal progenitor cells | CD44 | Retinal progenitor cells | -2.0419 | 4.3040 | -4.2028 | 0.0003 | 0.0055 | -0.3244 |
| Choroid-RPE complex | beta_actin | Choroid-RPE complex | -0.4707 | 0.3285 | -3.8199 | 0.0008 | 0.0075 | -1.7203 |
| Retinal progenitor cells | beta_actin | Retinal progenitor cells | -0.0168 | 0.0473 | -3.2603 | 0.0033 | 0.0162 | -2.6208 |
| Photoreceptors | beta_actin | Photoreceptors | -0.0205 | 0.0214 | -4.5223 | 0.0001 | 0.0030 | -0.6111 |
| Choroid-RPE complex | GranzymeB | Choroid-RPE complex | -1.2420 | 0.9201 | -3.7210 | 0.0011 | 0.0087 | -1.9639 |
| Choroid-RPE complex | F480 | Choroid-RPE complex | -1.1053 | 0.7971 | -4.2399 | 0.0003 | 0.0055 | -0.6703 |
| Choroid-RPE complex | Ecadherin | Choroid-RPE complex | -1.4697 | 1.0102 | -3.3805 | 0.0025 | 0.0143 | -2.7883 |
| Choroid-RPE complex | CD4 | Choroid-RPE complex | -1.2531 | 0.9584 | -3.3104 | 0.0029 | 0.0158 | -2.9549 |
| Choroid-RPE complex | iNOS_CD206 | Choroid-RPE complex | -2.2274 | 1.9107 | -3.4013 | 0.0023 | 0.0140 | -2.7388 |
| Choroid-RPE complex | MHCII | Choroid-RPE complex | -2.2868 | 1.7264 | -3.3233 | 0.0028 | 0.0156 | -2.9242 |
| Choroid-RPE complex | CD8 | Choroid-RPE complex | -1.3237 | 1.0130 | -3.2605 | 0.0033 | 0.0162 | -3.0723 |
| Choroid-RPE complex | CD11b | Choroid-RPE complex | -0.8518 | 0.6496 | -3.8393 | 0.0008 | 0.0075 | -1.6724 |
| Microglia | CD11b | Microglia | -0.6835 | 1.1398 | -2.8318 | 0.0097 | 0.0409 | -2.8696 |
| Choroid-RPE complex | Arginase_perk | Choroid-RPE complex | -6.2435 | 4.8082 | -3.4277 | 0.0022 | 0.0139 | -2.6756 |
| Perivascular cells | Arginase_perk | Perivascular cells | 0.9058 | 0.9012 | 3.0283 | 0.0069 | 0.0296 | -2.3106 |
| Retinal progenitor cells | Arginase_perk | Retinal progenitor cells | 0.2035 | 0.6697 | 3.2435 | 0.0034 | 0.0162 | -2.6599 |
| Photoreceptors | Arginase_perk | Photoreceptors | 0.1524 | 0.3530 | 3.2323 | 0.0035 | 0.0162 | -3.8032 |
| Choroid-RPE complex | Foxp3 | Choroid-RPE complex | -1.0424 | 0.8071 | -3.2155 | 0.0037 | 0.0165 | -3.1780 |
| Choroid-RPE complex | Ly6G | Choroid-RPE complex | -0.5375 | 0.4199 | -3.3249 | 0.0028 | 0.0156 | -2.9204 |
| Choroid-RPE complex | Beta_catenin | Choroid-RPE complex | -1.6861 | 1.4666 | -3.2608 | 0.0033 | 0.0162 | -3.0717 |
| Retinal progenitor cells | Beta_catenin | Retinal progenitor cells | 1.3057 | 1.5526 | 7.3714 | 0.0000 | 0.0000 | 7.4914 |
| Photoreceptors | Beta_catenin | Photoreceptors | 0.3413 | 0.5368 | 7.2905 | 0.0000 | 0.0000 | 6.2994 |
| Interneurons | Beta_catenin | Interneurons | 0.3080 | 0.5485 | 4.1336 | 0.0004 | 0.0055 | -1.3221 |
| Ganglion cells | Beta_catenin | Ganglion cells | 0.3534 | 0.5507 | 3.6164 | 0.0014 | 0.0106 | -1.9861 |
| Choroid-RPE complex | CD3 | Choroid-RPE complex | -2.1802 | 1.7040 | -3.4064 | 0.0023 | 0.0140 | -2.7265 |
| Choroid-RPE complex | CD31 | Choroid-RPE complex | -3.0417 | 2.3533 | -3.2513 | 0.0034 | 0.0162 | -3.0941 |
| Choroid-RPE complex | Pan_cytokeratin | Choroid-RPE complex | -1.0330 | 1.0688 | -4.9119 | 0.0001 | 0.0013 | 1.0369 |
| Choroid-RPE complex | B220 | Choroid-RPE complex | -0.3770 | 0.3382 | -5.0466 | 0.0000 | 0.0011 | 1.3803 |
| Choroid-RPE complex | DNA1 | Choroid-RPE complex | -50.5792 | 47.2815 | -3.9081 | 0.0007 | 0.0074 | -1.5017 |
| Microglia | DNA1 | Microglia | -26.9490 | 22.7409 | -7.0256 | 0.0000 | 0.0000 | 6.4573 |
| Unspecific cell types | DNA1 | Unspecific cell types | -45.2223 | 35.4673 | -4.1102 | 0.0004 | 0.0055 | -0.3922 |
| Choroid-RPE complex | DNA2 | Choroid-RPE complex | -93.0093 | 85.9798 | -3.8914 | 0.0007 | 0.0074 | -1.5431 |
| Microglia | DNA2 | Microglia | -48.0793 | 40.9567 | -6.3555 | 0.0000 | 0.0001 | 5.0468 |
| Unspecific cell types | DNA2 | Unspecific cell types | -83.9863 | 65.0935 | -4.0943 | 0.0004 | 0.0055 | -0.4317 |

**IMC differential state (C vs Bel)**

| cluster_id | marker_id | ID | logFC | AveExpr | t | *p*_val | p_adj | B |
| --- | --- | --- | --- | --- | --- | --- | --- | --- |
| Choroid-RPE complex | αSMA | Choroid-RPE complex | -0.8844 | 0.7295 | -4.3380 | 0.0002 | 0.0068 | -0.4202 |
| Microglia | αSMA | Microglia | -0.3527 | 0.3074 | -3.2682 | 0.0035 | 0.0209 | -1.8378 |
| Interneurons | αSMA | Interneurons | -0.0763 | 0.1770 | -3.0394 | 0.0056 | 0.0282 | -3.9281 |
| Choroid-RPE complex | pTyrosine | Choroid-RPE complex | -1.8980 | 1.4739 | -3.5760 | 0.0015 | 0.0138 | -2.3157 |
| Microglia | pTyrosine | Microglia | -0.6102 | 0.5019 | -3.6814 | 0.0013 | 0.0138 | -0.9328 |
| Retinal progenitor cells | pTyrosine | Retinal progenitor cells | 0.0657 | 0.1286 | 3.8013 | 0.0009 | 0.0138 | -1.1088 |
| Photoreceptors | pTyrosine | Photoreceptors | 0.0264 | 0.0739 | 3.6961 | 0.0011 | 0.0138 | -2.6695 |
| Retinal progenitor cells | Vimentin | Retinal progenitor cells | -0.7388 | 0.9464 | -3.4795 | 0.0019 | 0.0138 | -1.8853 |
| Ganglion cells | Vimentin | Ganglion cells | 1.3305 | 2.9468 | 2.8169 | 0.0095 | 0.0456 | -3.7624 |
| Choroid-RPE complex | Ki67 | Choroid-RPE complex | -0.5920 | 0.4485 | -3.5216 | 0.0017 | 0.0138 | -2.4475 |
| Choroid-RPE complex | CD45 | Choroid-RPE complex | -0.4275 | 0.3496 | -3.4892 | 0.0019 | 0.0138 | -2.5255 |
| Choroid-RPE complex | Fibronectin | Choroid-RPE complex | -1.1711 | 1.0011 | -4.2298 | 0.0003 | 0.0078 | -0.6936 |
| Ganglion cells | CD44 | Ganglion cells | 0.5305 | 0.7221 | 3.2102 | 0.0037 | 0.0217 | -2.8709 |
| Choroid-RPE complex | beta_actin | Choroid-RPE complex | -0.4481 | 0.3285 | -3.6311 | 0.0013 | 0.0138 | -2.1815 |
| Photoreceptors | beta_actin | Photoreceptors | -0.0166 | 0.0214 | -3.6172 | 0.0014 | 0.0138 | -2.8629 |
| Choroid-RPE complex | GranzymeB | Choroid-RPE complex | -1.1824 | 0.9201 | -3.5375 | 0.0017 | 0.0138 | -2.4091 |
| Choroid-RPE complex | F480 | Choroid-RPE complex | -0.9562 | 0.7971 | -3.6628 | 0.0012 | 0.0138 | -2.1043 |
| Choroid-RPE complex | CD4 | Choroid-RPE complex | -1.3216 | 0.9584 | -3.4865 | 0.0019 | 0.0138 | -2.5321 |
| Choroid-RPE complex | iNOS_CD206 | Choroid-RPE complex | -2.1377 | 1.9107 | -3.2597 | 0.0033 | 0.0204 | -3.0720 |
| Choroid-RPE complex | MHCII | Choroid-RPE complex | -2.4433 | 1.7264 | -3.5458 | 0.0016 | 0.0138 | -2.3889 |
| Microglia | MHCII | Microglia | -0.6162 | 0.3150 | -2.9122 | 0.0080 | 0.0394 | -2.5903 |
| Choroid-RPE complex | CD8 | Choroid-RPE complex | -1.4326 | 1.0130 | -3.5238 | 0.0017 | 0.0138 | -2.4422 |
| Microglia | CD8 | Microglia | -0.3610 | 0.2098 | -3.0664 | 0.0056 | 0.0282 | -2.2682 |
| Choroid-RPE complex | CD11b | Choroid-RPE complex | -0.8245 | 0.6496 | -3.7106 | 0.0011 | 0.0138 | -1.9871 |
| Choroid-RPE complex | Arginase_perk | Choroid-RPE complex | -6.6270 | 4.8082 | -3.6331 | 0.0013 | 0.0138 | -2.1768 |
| Choroid-RPE complex | Foxp3 | Choroid-RPE complex | -1.1520 | 0.8071 | -3.5484 | 0.0016 | 0.0138 | -2.3827 |
| Choroid-RPE complex | Ly6G | Choroid-RPE complex | -0.5931 | 0.4199 | -3.6640 | 0.0012 | 0.0138 | -2.1013 |
| Choroid-RPE complex | Beta_catenin | Choroid-RPE complex | -1.8801 | 1.4666 | -3.6308 | 0.0013 | 0.0138 | -2.1824 |
| Retinal progenitor cells | Beta_catenin | Retinal progenitor cells | 1.3084 | 1.5526 | 5.8147 | 0.0000 | 0.0006 | 3.9139 |
| Photoreceptors | Beta_catenin | Photoreceptors | 0.3762 | 0.5368 | 7.9040 | 0.0000 | 0.0000 | 7.7464 |
| Interneurons | Beta_catenin | Interneurons | 0.3485 | 0.5485 | 4.4709 | 0.0002 | 0.0068 | -0.4227 |
| Ganglion cells | Beta_catenin | Ganglion cells | 0.4657 | 0.5507 | 4.3697 | 0.0002 | 0.0068 | -0.0325 |
| Choroid-RPE complex | CD3 | Choroid-RPE complex | -2.3898 | 1.7040 | -3.7287 | 0.0010 | 0.0138 | -1.9428 |
| Choroid-RPE complex | CD31 | Choroid-RPE complex | -3.2305 | 2.3533 | -3.4482 | 0.0021 | 0.0141 | -2.6242 |
| Microglia | CD31 | Microglia | -0.9068 | 0.5592 | -3.7151 | 0.0012 | 0.0138 | -0.8580 |
| Retinal progenitor cells | CD31 | Retinal progenitor cells | 0.0444 | 0.1019 | 2.7796 | 0.0104 | 0.0487 | -3.4841 |
| Microglia | CollagenT1 | Microglia | -0.7082 | 0.4939 | -3.4827 | 0.0021 | 0.0141 | -1.3714 |
| Choroid-RPE complex | Pan_cytokeratin | Choroid-RPE complex | -1.1187 | 1.0688 | -5.3116 | 0.0000 | 0.0010 | 2.0570 |
| Choroid-RPE complex | B220 | Choroid-RPE complex | -0.4178 | 0.3382 | -5.5838 | 0.0000 | 0.0007 | 2.7466 |
| Microglia | B220 | Microglia | -0.0936 | 0.0589 | -3.1989 | 0.0041 | 0.0228 | -1.9867 |
| Choroid-RPE complex | DNA1 | Choroid-RPE complex | -43.5089 | 47.2815 | -3.3570 | 0.0026 | 0.0170 | -2.8419 |
| Microglia | DNA1 | Microglia | -16.2268 | 22.7409 | -3.5598 | 0.0017 | 0.0138 | -1.2019 |
| Unspecific cell types | DNA1 | Unspecific cell types | -40.2889 | 35.4673 | -3.0508 | 0.0055 | 0.0282 | -2.7664 |
| Choroid-RPE complex | DNA2 | Choroid-RPE complex | -79.3970 | 85.9798 | -3.3172 | 0.0029 | 0.0182 | -2.9364 |
| Microglia | DNA2 | Microglia | -28.8702 | 40.9567 | -3.2113 | 0.0040 | 0.0227 | -1.9601 |
| Unspecific cell types | DNA2 | Unspecific cell types | -75.1674 | 65.0935 | -3.0530 | 0.0054 | 0.0282 | -2.7615 |

**IMC differential state (LC vs Fas)**

| cluster_id | marker_id | ID | logFC | AveExpr | t | *p*_val | p_adj | B |
| --- | --- | --- | --- | --- | --- | --- | --- | --- |
| Perivascular cells | αSMA | Perivascular cells | 11.5753 | 16.5474 | 2.8193 | 0.0109 | 0.0348 | -3.2715 |
| Photoreceptors | αSMA | Photoreceptors | -0.1540 | 0.2596 | -2.6735 | 0.0132 | 0.0413 | -5.1031 |
| Unspecific cell types | αSMA | Unspecific cell types | 0.6952 | 0.3142 | 3.8883 | 0.0007 | 0.0039 | -1.6426 |
| Perivascular cells | pTyrosine | Perivascular cells | 0.1148 | 0.1941 | 2.9821 | 0.0076 | 0.0260 | -2.9334 |
| Photoreceptors | pTyrosine | Photoreceptors | 0.0245 | 0.0739 | 2.8260 | 0.0093 | 0.0311 | -4.7705 |
| Unspecific cell types | pTyrosine | Unspecific cell types | 0.9497 | 0.3328 | 3.3524 | 0.0026 | 0.0112 | -2.9475 |
| Choroid-RPE complex | Vimentin | Choroid-RPE complex | 0.7814 | 0.8316 | 4.6588 | 0.0001 | 0.0008 | -0.1193 |
| Perivascular cells | Vimentin | Perivascular cells | 10.6334 | 5.9541 | 7.6791 | 0.0000 | 0.0000 | 7.0226 |
| Microglia | Vimentin | Microglia | 12.9908 | 5.0747 | 4.4687 | 0.0002 | 0.0015 | 0.8287 |
| Photoreceptors | Vimentin | Photoreceptors | 0.4312 | 0.2770 | 6.2994 | 0.0000 | 0.0000 | 3.8552 |
| Interneurons | Vimentin | Interneurons | 3.6063 | 1.6610 | 9.4413 | 0.0000 | 0.0000 | 11.2228 |
| Ganglion cells | Vimentin | Ganglion cells | 8.1846 | 2.9468 | 18.0256 | 0.0000 | 0.0000 | 25.6044 |
| Unspecific cell types | Vimentin | Unspecific cell types | 2.7323 | 2.4156 | 5.3193 | 0.0000 | 0.0002 | 1.9852 |
| Perivascular cells | Ki67 | Perivascular cells | 0.2166 | 0.1800 | 4.6353 | 0.0002 | 0.0014 | 0.7104 |
| Photoreceptors | Ki67 | Photoreceptors | 0.0227 | 0.0186 | 3.1056 | 0.0048 | 0.0179 | -4.1381 |
| Interneurons | Ki67 | Interneurons | 0.0746 | 0.0544 | 8.0827 | 0.0000 | 0.0000 | 8.2626 |
| Ganglion cells | Ki67 | Ganglion cells | 0.1607 | 0.0838 | 9.2263 | 0.0000 | 0.0000 | 11.2800 |
| Unspecific cell types | Ki67 | Unspecific cell types | 0.4052 | 0.1483 | 4.2260 | 0.0003 | 0.0021 | -0.7964 |
| Retinal progenitor cells | CD45 | Retinal progenitor cells | -0.0358 | 0.0638 | -6.3142 | 0.0000 | 0.0000 | 4.4736 |
| Unspecific cell types | CD45 | Unspecific cell types | 0.3083 | 0.0922 | 3.9193 | 0.0006 | 0.0037 | -1.5654 |
| Retinal progenitor cells | Fibronectin | Retinal progenitor cells | -0.0771 | 0.0478 | -6.0348 | 0.0000 | 0.0000 | 3.7797 |
| Photoreceptors | Fibronectin | Photoreceptors | -0.0632 | 0.0362 | -5.6662 | 0.0000 | 0.0001 | 2.2672 |
| Unspecific cell types | Fibronectin | Unspecific cell types | 0.6463 | 0.2559 | 3.0547 | 0.0054 | 0.0197 | -3.6428 |
| Macrophages | CD44 | Macrophages | 0.9832 | 1.0502 | 3.0894 | 0.0063 | 0.0224 | -2.6261 |
| Retinal progenitor cells | CD44 | Retinal progenitor cells | -2.4606 | 4.3040 | -7.0361 | 0.0000 | 0.0000 | 6.2293 |
| Photoreceptors | CD44 | Photoreceptors | 0.3682 | 1.0940 | 2.7815 | 0.0103 | 0.0339 | -4.8686 |
| Ganglion cells | CD44 | Ganglion cells | 0.5506 | 0.7221 | 3.4662 | 0.0020 | 0.0091 | -2.5435 |
| Unspecific cell types | CD44 | Unspecific cell types | 1.3382 | 1.1038 | 3.2693 | 0.0032 | 0.0131 | -3.1442 |
| Perivascular cells | beta_actin | Perivascular cells | 0.2102 | 0.2207 | 2.8892 | 0.0093 | 0.0311 | -3.1274 |
| Retinal progenitor cells | beta_actin | Retinal progenitor cells | -0.0149 | 0.0473 | -4.0119 | 0.0005 | 0.0032 | -1.3449 |
| Interneurons | beta_actin | Interneurons | 0.0250 | 0.0426 | 4.5599 | 0.0001 | 0.0010 | -0.3858 |
| Ganglion cells | beta_actin | Ganglion cells | 0.0509 | 0.0639 | 3.7874 | 0.0009 | 0.0046 | -1.7610 |
| Unspecific cell types | beta_actin | Unspecific cell types | 0.2474 | 0.0991 | 3.9602 | 0.0006 | 0.0035 | -1.4636 |
| Unspecific cell types | GranzymeB | Unspecific cell types | 0.5962 | 0.1835 | 3.3028 | 0.0030 | 0.0124 | -3.0652 |
| Unspecific cell types | F480 | Unspecific cell types | 0.8049 | 0.1928 | 3.8755 | 0.0007 | 0.0039 | -1.6743 |
| Perivascular cells | Ecadherin | Perivascular cells | 0.9203 | 0.6193 | 4.5823 | 0.0002 | 0.0015 | 0.5920 |
| Retinal progenitor cells | Ecadherin | Retinal progenitor cells | 0.0735 | 0.1936 | 3.6606 | 0.0012 | 0.0058 | -2.2143 |
| Photoreceptors | Ecadherin | Photoreceptors | 0.0897 | 0.1581 | 4.2131 | 0.0003 | 0.0021 | -1.4357 |
| Interneurons | Ecadherin | Interneurons | 0.2887 | 0.4527 | 3.7543 | 0.0010 | 0.0048 | -2.4136 |
| Ganglion cells | Ecadherin | Ganglion cells | 0.9061 | 0.6962 | 8.5828 | 0.0000 | 0.0000 | 9.8999 |
| Unspecific cell types | Ecadherin | Unspecific cell types | 1.4174 | 0.4608 | 5.9249 | 0.0000 | 0.0001 | 3.5149 |
| Retinal progenitor cells | CD4 | Retinal progenitor cells | -0.0184 | 0.0310 | -3.4582 | 0.0020 | 0.0091 | -2.7053 |
| Unspecific cell types | CD4 | Unspecific cell types | 0.6509 | 0.1815 | 3.3748 | 0.0025 | 0.0110 | -2.8944 |
| Unspecific cell types | iNOS_CD206 | Unspecific cell types | 1.8107 | 0.4541 | 4.1824 | 0.0003 | 0.0022 | -0.9063 |
| Unspecific cell types | MHCII | Unspecific cell types | 1.0365 | 0.2891 | 3.2092 | 0.0037 | 0.0149 | -3.2853 |
| Unspecific cell types | CD8 | Unspecific cell types | 0.6552 | 0.1867 | 3.3609 | 0.0026 | 0.0112 | -2.9273 |
| Retinal progenitor cells | CD11b | Retinal progenitor cells | -0.0151 | 0.0399 | -2.9580 | 0.0068 | 0.0237 | -3.8732 |
| Unspecific cell types | CD11b | Unspecific cell types | 0.5519 | 0.1468 | 3.8584 | 0.0007 | 0.0040 | -1.7167 |
| Perivascular cells | Arginase_perk | Perivascular cells | 1.0574 | 0.9012 | 6.2734 | 0.0000 | 0.0001 | 4.2517 |
| Retinal progenitor cells | Arginase_perk | Retinal progenitor cells | 0.4361 | 0.6697 | 9.6570 | 0.0000 | 0.0000 | 12.0568 |
| Photoreceptors | Arginase_perk | Photoreceptors | 0.2386 | 0.3530 | 4.0999 | 0.0004 | 0.0027 | -1.7214 |
| Interneurons | Arginase_perk | Interneurons | 0.4791 | 0.6487 | 3.6924 | 0.0011 | 0.0055 | -2.5658 |
| Ganglion cells | Arginase_perk | Ganglion cells | 0.7319 | 0.7474 | 2.7690 | 0.0106 | 0.0344 | -4.1492 |
| Unspecific cell types | Arginase_perk | Unspecific cell types | 3.5422 | 1.2290 | 3.7998 | 0.0009 | 0.0046 | -1.8618 |
| Unspecific cell types | Foxp3 | Unspecific cell types | 0.4940 | 0.1449 | 3.0887 | 0.0050 | 0.0184 | -3.5646 |
| Unspecific cell types | Ly6G | Unspecific cell types | 0.3086 | 0.0973 | 3.6157 | 0.0014 | 0.0064 | -2.3136 |
| Perivascular cells | Beta_catenin | Perivascular cells | 1.4861 | 0.7428 | 7.4583 | 0.0000 | 0.0000 | 6.6061 |
| Microglia | Beta_catenin | Microglia | 2.6773 | 1.7946 | 5.8310 | 0.0000 | 0.0001 | 3.8733 |
| Macrophages | Beta_catenin | Macrophages | 0.3148 | 0.3229 | 3.2590 | 0.0043 | 0.0164 | -2.2713 |
| Retinal progenitor cells | Beta_catenin | Retinal progenitor cells | 1.8817 | 1.5526 | 14.7580 | 0.0000 | 0.0000 | 21.0431 |
| Photoreceptors | Beta_catenin | Photoreceptors | 0.8291 | 0.5368 | 14.3522 | 0.0000 | 0.0000 | 19.9508 |
| Interneurons | Beta_catenin | Interneurons | 0.8003 | 0.5485 | 9.6934 | 0.0000 | 0.0000 | 11.7459 |
| Ganglion cells | Beta_catenin | Ganglion cells | 0.8874 | 0.5507 | 8.6607 | 0.0000 | 0.0000 | 10.0698 |
| Unspecific cell types | Beta_catenin | Unspecific cell types | 2.5063 | 0.8618 | 5.6688 | 0.0000 | 0.0001 | 2.8709 |
| Unspecific cell types | CD3 | Unspecific cell types | 1.0377 | 0.3485 | 3.1871 | 0.0039 | 0.0152 | -3.3368 |
| Unspecific cell types | CD31 | Unspecific cell types | 2.1445 | 0.5075 | 3.9733 | 0.0006 | 0.0035 | -1.4308 |
| Choroid-RPE complex | CollagenT1 | Choroid-RPE complex | 21.1795 | 7.6230 | 5.1358 | 0.0000 | 0.0003 | 1.0998 |
| Retinal progenitor cells | CollagenT1 | Retinal progenitor cells | 0.4995 | 0.3086 | 3.9535 | 0.0006 | 0.0035 | -1.4906 |
| Photoreceptors | CollagenT1 | Photoreceptors | 0.6637 | 0.4287 | 5.2041 | 0.0000 | 0.0002 | 1.0917 |
| Interneurons | CollagenT1 | Interneurons | 0.3299 | 0.2718 | 5.3435 | 0.0000 | 0.0002 | 1.6153 |
| Choroid-RPE complex | Pan_cytokeratin | Choroid-RPE complex | 1.0711 | 1.0688 | 6.7271 | 0.0000 | 0.0000 | 5.0869 |
| Photoreceptors | Pan_cytokeratin | Photoreceptors | 0.0591 | 0.0400 | 5.1803 | 0.0000 | 0.0002 | 1.0308 |
| Interneurons | Pan_cytokeratin | Interneurons | 0.0360 | 0.0435 | 3.2879 | 0.0031 | 0.0127 | -3.5418 |
| Unspecific cell types | Pan_cytokeratin | Unspecific cell types | 0.7245 | 0.2543 | 2.7375 | 0.0114 | 0.0361 | -4.3509 |
| Choroid-RPE complex | B220 | Choroid-RPE complex | 0.1680 | 0.3382 | 2.9695 | 0.0066 | 0.0234 | -4.2646 |
| Unspecific cell types | B220 | Unspecific cell types | 0.2441 | 0.0654 | 3.1931 | 0.0039 | 0.0152 | -3.3229 |
| Microglia | DNA1 | Microglia | -23.9317 | 22.7409 | -4.2939 | 0.0003 | 0.0021 | 0.4312 |
| Microglia | DNA2 | Microglia | -41.9940 | 40.9567 | -3.8204 | 0.0009 | 0.0047 | -0.6407 |

**IMC differential state (LC vs Bel)**

| cluster_id | marker_id | ID | logFC | AveExpr | t | *p*_val | p_adj | B |
| --- | --- | --- | --- | --- | --- | --- | --- | --- |
| Perivascular cells | αSMA | Perivascular cells | 17.0520 | 16.5474 | 3.0565 | 0.0064 | 0.0384 | -2.3003 |
| Photoreceptors | pTyrosine | Photoreceptors | 0.0325 | 0.0739 | 3.7099 | 0.0011 | 0.0085 | -2.2904 |
| Interneurons | pTyrosine | Interneurons | 0.0440 | 0.0837 | 3.0874 | 0.0050 | 0.0320 | -3.5821 |
| Choroid-RPE complex | Vimentin | Choroid-RPE complex | 0.7670 | 0.8316 | 4.5616 | 0.0001 | 0.0012 | 0.0210 |
| Perivascular cells | Vimentin | Perivascular cells | 9.6464 | 5.9541 | 5.1266 | 0.0001 | 0.0006 | 1.9220 |
| Microglia | Vimentin | Microglia | 13.9673 | 5.0747 | 4.3947 | 0.0002 | 0.0020 | 0.5400 |
| Retinal progenitor cells | Vimentin | Retinal progenitor cells | -0.5299 | 0.9464 | -2.9801 | 0.0065 | 0.0384 | -3.0712 |
| Photoreceptors | Vimentin | Photoreceptors | 0.3799 | 0.2770 | 5.4900 | 0.0000 | 0.0002 | 2.2166 |
| Interneurons | Vimentin | Interneurons | 3.4286 | 1.6610 | 8.6486 | 0.0000 | 0.0000 | 9.9176 |
| Ganglion cells | Vimentin | Ganglion cells | 7.9615 | 2.9468 | 16.1948 | 0.0000 | 0.0000 | 23.1657 |
| Unspecific cell types | Vimentin | Unspecific cell types | 2.2163 | 2.4156 | 2.9016 | 0.0078 | 0.0440 | -3.2308 |
| Perivascular cells | Ki67 | Perivascular cells | 0.2158 | 0.1800 | 3.3990 | 0.0030 | 0.0213 | -1.6112 |
| Interneurons | Ki67 | Interneurons | 0.0758 | 0.0544 | 7.9050 | 0.0000 | 0.0000 | 8.2570 |
| Ganglion cells | Ki67 | Ganglion cells | 0.1629 | 0.0838 | 8.6407 | 0.0000 | 0.0000 | 10.3534 |
| Retinal progenitor cells | CD45 | Retinal progenitor cells | -0.0273 | 0.0638 | -3.2653 | 0.0033 | 0.0220 | -2.4185 |
| Interneurons | Fibronectin | Interneurons | 0.0367 | 0.0454 | 2.9700 | 0.0066 | 0.0384 | -3.8499 |
| Photoreceptors | CD44 | Photoreceptors | 0.6790 | 1.0940 | 5.0731 | 0.0000 | 0.0004 | 1.1549 |
| Interneurons | CD44 | Interneurons | 0.5332 | 0.8527 | 4.3392 | 0.0002 | 0.0020 | -0.5245 |
| Ganglion cells | CD44 | Ganglion cells | 0.9324 | 0.7221 | 5.4208 | 0.0000 | 0.0002 | 2.7856 |
| Interneurons | beta_actin | Interneurons | 0.0328 | 0.0426 | 5.7611 | 0.0000 | 0.0001 | 3.0897 |
| Ganglion cells | beta_actin | Ganglion cells | 0.0650 | 0.0639 | 4.4651 | 0.0002 | 0.0015 | 0.3729 |
| Perivascular cells | Ecadherin | Perivascular cells | 0.8430 | 0.6193 | 3.0891 | 0.0060 | 0.0373 | -2.2355 |
| Retinal progenitor cells | Ecadherin | Retinal progenitor cells | 0.0946 | 0.1936 | 3.1890 | 0.0039 | 0.0257 | -2.5956 |
| Photoreceptors | Ecadherin | Photoreceptors | 0.1251 | 0.1581 | 5.8100 | 0.0000 | 0.0001 | 3.0258 |
| Interneurons | Ecadherin | Interneurons | 0.3904 | 0.4527 | 4.8913 | 0.0001 | 0.0006 | 0.8807 |
| Ganglion cells | Ecadherin | Ganglion cells | 1.0302 | 0.6962 | 9.0122 | 0.0000 | 0.0000 | 11.1413 |
| Perivascular cells | Arginase_perk | Perivascular cells | 0.9067 | 0.9012 | 3.9584 | 0.0008 | 0.0067 | -0.4614 |
| Retinal progenitor cells | Arginase_perk | Retinal progenitor cells | 0.3719 | 0.6697 | 5.5724 | 0.0000 | 0.0002 | 3.2907 |
| Photoreceptors | Arginase_perk | Photoreceptors | 0.1931 | 0.3530 | 3.2827 | 0.0031 | 0.0217 | -3.3211 |
| Interneurons | Arginase_perk | Interneurons | 0.4747 | 0.6487 | 3.5246 | 0.0017 | 0.0131 | -2.5490 |
| Ganglion cells | Arginase_perk | Ganglion cells | 0.9485 | 0.7474 | 3.3142 | 0.0029 | 0.0213 | -2.4538 |
| Perivascular cells | Beta_catenin | Perivascular cells | 1.3490 | 0.7428 | 4.9825 | 0.0001 | 0.0008 | 1.6339 |
| Microglia | Beta_catenin | Microglia | 3.2036 | 1.7946 | 6.3819 | 0.0000 | 0.0000 | 4.3662 |
| Retinal progenitor cells | Beta_catenin | Retinal progenitor cells | 1.8844 | 1.5526 | 9.9993 | 0.0000 | 0.0000 | 13.1864 |
| Photoreceptors | Beta_catenin | Photoreceptors | 0.8640 | 0.5368 | 14.7935 | 0.0000 | 0.0000 | 20.9627 |
| Interneurons | Beta_catenin | Interneurons | 0.8409 | 0.5485 | 9.8131 | 0.0000 | 0.0000 | 12.3723 |
| Ganglion cells | Beta_catenin | Ganglion cells | 0.9997 | 0.5507 | 9.0113 | 0.0000 | 0.0000 | 11.1395 |
| Choroid-RPE complex | CollagenT1 | Choroid-RPE complex | 20.3096 | 7.6230 | 4.9127 | 0.0001 | 0.0006 | 0.9161 |
| Photoreceptors | CollagenT1 | Photoreceptors | 0.7079 | 0.4287 | 5.4904 | 0.0000 | 0.0002 | 2.2178 |
| Interneurons | CollagenT1 | Interneurons | 0.3478 | 0.2718 | 5.4275 | 0.0000 | 0.0002 | 2.2463 |
| Choroid-RPE complex | Pan_cytokeratin | Choroid-RPE complex | 0.9854 | 1.0688 | 6.1738 | 0.0000 | 0.0000 | 4.1036 |
| Photoreceptors | Pan_cytokeratin | Photoreceptors | 0.0583 | 0.0400 | 5.0581 | 0.0000 | 0.0004 | 1.1165 |
| Interneurons | Pan_cytokeratin | Interneurons | 0.0434 | 0.0435 | 3.8231 | 0.0008 | 0.0067 | -1.8184 |
| Perivascular cells | DNA1 | Perivascular cells | -14.0285 | 23.3689 | -2.9229 | 0.0087 | 0.0479 | -2.5635 |

**IMC differential state (Fas vs Bel)**

| cluster_id | marker_id | ID | logFC | AveExpr | t | *p*_val | p_adj | B |
| --- | --- | --- | --- | --- | --- | --- | --- | --- |
| Photoreceptors | Fibronectin | Photoreceptors | 0.0640 | 0.0362 | 6.236 | 0.0000 | 0.0004 | 4.9797 |

| **Table S13. Antibody list** | | | | | | |
| --- | --- | --- | --- | --- | --- | --- |
|  | **name** | **Cata Lot NO.** | **Dilution** | | **Type** | **Company** |
| 1^st^ Antibodies | p-MYPT1 | PA5-10529 | WB 1:500 | IHC 1:50 | Rabbit Polyclonal | Thermo Fisher Scientific |
|  | MYPT1 | sc-51426 | WB 1:100 | IF 1:50 | Mouse Polyclonal | Santa Cruz Biotechnology |
|  | ROCK1 | sc-17794 | WB 1:100 | IHC 1:50 | Mouse Polyclonal | Santa Cruz Biotechnology |
|  | ROCK2 | sc-398519 | WB 1:100 | IHC 1:50 | Mouse Polyclonal | Santa Cruz Biotechnology |
|  | TGF-β1 | ab215715 | WB 1:1000 | IHC 1:500 | Rabbit Polyclonal | Abcam |
|  | CCL19 | LS‑C372455 | WB 1:50 | | Rabbit Polyclonal | LSBio |
|  | Type 1 collagen | ab34710 | WB 1:1000 | IHC 1:200 | Rabbit Polyclonal | Abcam |
|  | Fibronectin | ab2413 | WB 1:1000 | IHC 1:100 | Rabbit Polyclonal | Abcam |
|  | Vimentin | ab137321 | WB 1:500 | IHC 1:100 | Rabbit Polyclonal | Abcam |
|  | Isolectin GS-IB4 | I21411 | IHC 1:200 | |  | Thermo Fisher Scientific |
|  | α-SMA | ab15734 | WB 1:1000 | IHC 1:200 | Rabbit Polyclonal | Abcam |
|  | β-actin | 8H10D10 | WB 1:1000 | | Mouse Polyclonal | Cell Signaling Technology |
| 2^nd^ Antibodies | 680RD donkey anti-mouse IgG IRDye® | 926-68072 | WB 1:5000 | IHC 1:100 | Mouse Polyclonal | LI-COR Biosciences |
|  | 800CW donkey anti-rabbit IgG IRDye® | 926-32213 | WB 1:5000 | IHC 1:100 | Rabbit Polyclonal | LI-COR Biosciences |

**Figure F1. Boxplot of fibrosis-related markers in IMC**

**
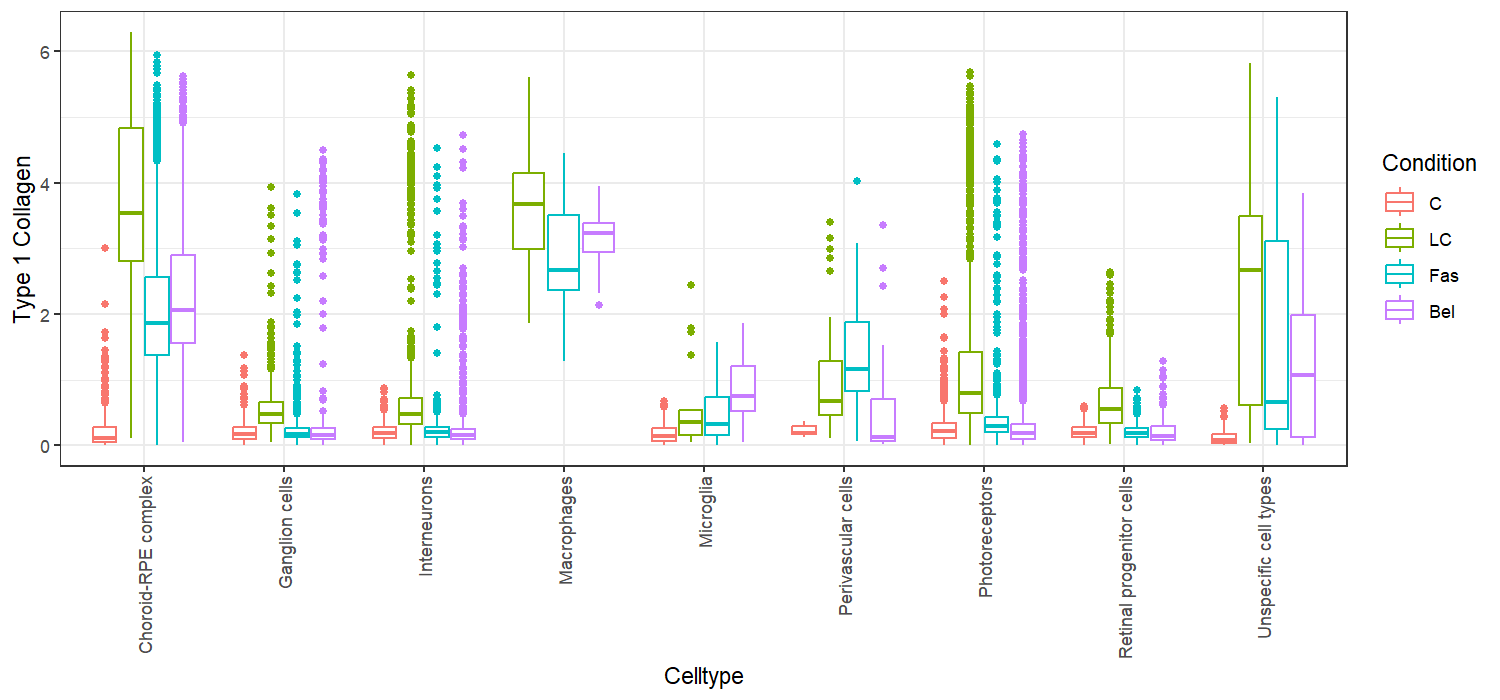

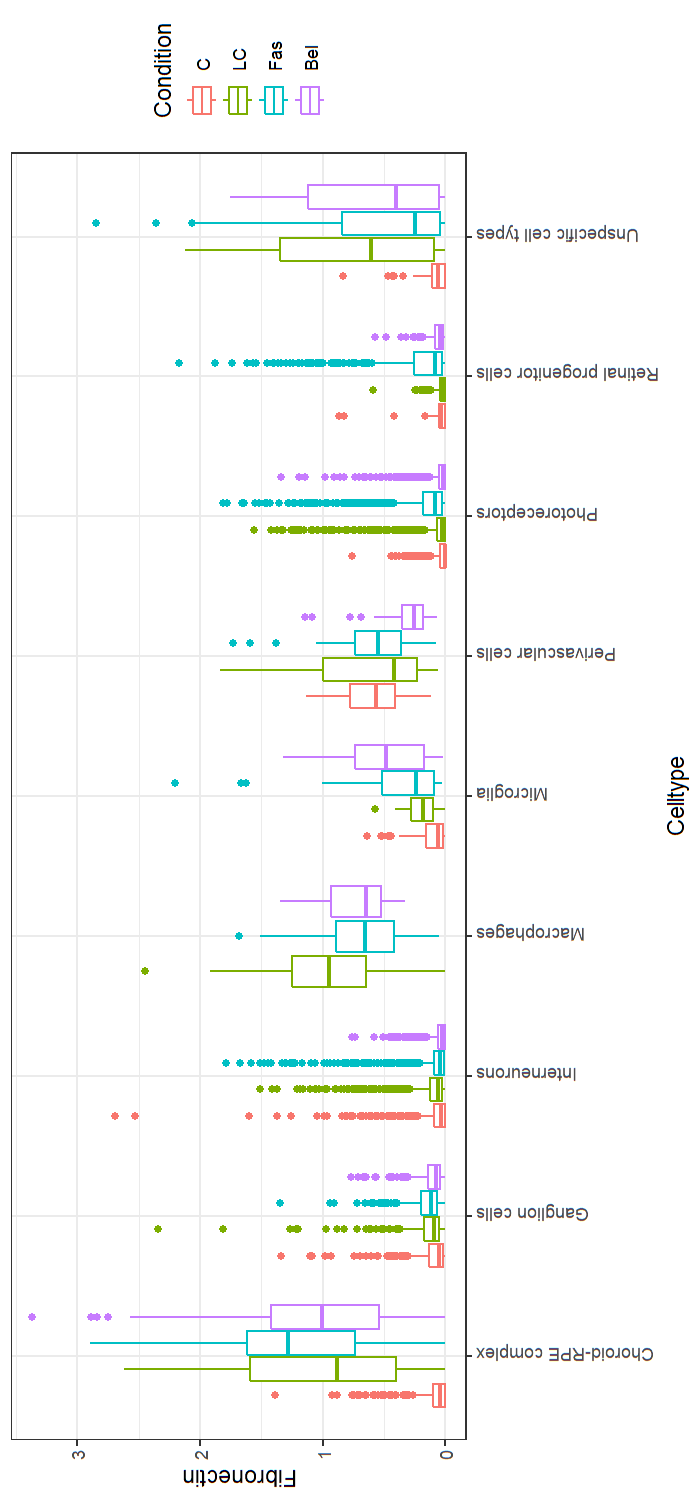

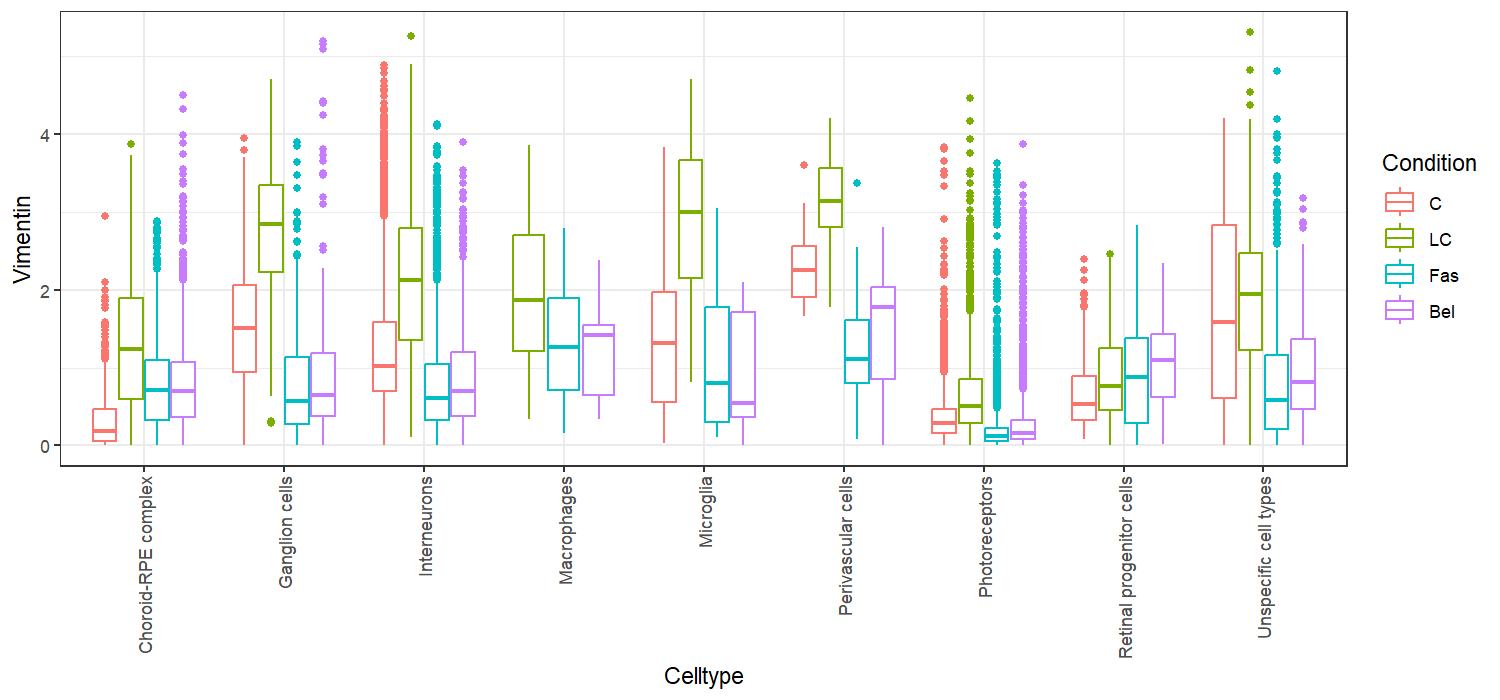
**
