# Supplementary material for: Rho-kinase inhibition reduces subretinal fibrosis
Source: Cell Death Discov. 2025 Oct 6;11:428. doi: 10.1038/s41420-025-02709-0 (PMC12501381; doi:10.1038/s41420-025-02709-0)
Supplement: Supplementary file 2 — Additional Materials and Methods [file 41420_2025_2709_MOESM2_ESM.pdf]

Additional Materials and Methods

Animals

All animal experiments adhered to the Statement for the Use of Animals in Ophthalmic and Vision Research (Association of Research in Visual Sciences and Ophthalmology). Protocols were approved by the The cantonal committee on animal experimentation of Bern (BE 146/2020). Mixed-sex C57BL/6J mice (Charles River Germany, Sulzfeld, Germany), 8-10 weeks of age, weighing 20-25 g were used. Animals were maintained on standard laboratory chow and water ad libitum in individually ventilated cages (IVC) in a temperature-controlled animal facility with a 12-hour circadian cycle. A group size of n = 6 per condition. Animals were included if they successfully received the full intervention procedure (Laser induction and intraperitoneal injection treament) without complications and survived until the designated endpoint. Animals were excluded if there was evidence of surgical failure, infection, or any unrelated health issue (trauma, weight loss >20%,cataracts, endophthalmitis and severe infection) that could confound interpretation of the fibrotic outcome. All exclusions and reasons were recorded before unblinding. Animals were randomly assigned to experimental groups using an Excel-generated randomization list to avoid allocation bias. Experimental group assignment was completed before treatment by volunteers from other laboratories who were not involved in outcome assessment. All subsequent analyses were performed blindly to the experimenters to ensure objectivity.

Diliation formula: with 2.5% phenylephrine and 0.5% tropicamide (ISPI, Bern, Switzerland)

Laser

Six spots (100 mW, 50 µm, 100 ms) lesions were located 2 to 3 disc diameters from the optic disc and between each vessel to avoid damage to the vessels. Bruch's membrane rupture was confirmed by the development of a bubble under the laser spot. Eyes with hemorrhage, severe cataract, infection or undamaged Bruch's membrane (without bubble formation after the laser) were excluded. A subretinal fibrosis developed 35 days after induction without active CNV.

Fluorescein angiography (FA), Fundus autofluorescence imaging (AF) and Optical coherence tomography (OCT)

Fluorescein injections were performed subcutaneously with 50 µl of 0.01% fluorescein [Faure; Novartis, Basel, Switzerland; in phosphate-buffered saline (PBS)]. Thereby, autofluorescence imaging (AF) reveals the fundus's natural fluorescence, while fluorescein angiography (FA) detects fluorescence leakage and the condition of retinal blood vessels. Optical coherence tomography (OCT) allows for the observation of cross-sectional retinal lesions at various depths within the mouse retina. Optical coherence tomography (OCT; Heidelberg Spectralis HRA2, Heidelberg Engineering GmbH, Germany) and confocal microscopy (Zeiss LSM 900; Zeiss, Oberkochen, Germany) were used to quantify the volume of CNV and fibrosis.

Lesion quantification (FA)

The volume of CNV lesions and subretinal fibrosis were measured based on the criteria depicted in Figure 1 on the basis of references (1,2) at the designated time points after laser injury. Choroid flatmounts were imaged with confocal microscopy and the Z stack images were measured with Image J. For the preparation of flat mounts, eyes were enucleated immediately after euthanasia and the anterior segment and the retina were removed from the eyecup. The remaining retinal pigment epithelium (RPE)-choroidsclera complex was flat-mounted using mounting medium (FM 100119; Thermo Fisher Scientific, Waltham, MA, United States) and coverslips after relaxing radial incisions. Afterwards, and staining for CNV (isolectin Isolectin GS-IB4; Thermo Fisher Scientific) and fibrosis (anti-type 1 collagen; Abcam, Cambridge, United Kingdom) was performed every week after laser injury (d7, d21, d35, d49, n=6 mice/10 eyes/60 lesions per time point). Optical coherence tomography (OCT; Heidelberg Spectralis HRA2, Heidelberg Engineering GmbH, Germany) and confocal microscopy (Zeiss LSM 900; Zeiss, Oberkochen, Germany) were used to quantify the volume of CNV and fibrosis. The extent of CNV and fibrotic lesions of the flat mounts were determined separately by using FIJI/Image J software (3). To document changes in CNV and fibrosis over time, we also performed autofluorescence and fluorescence angiography at each time point.

A)

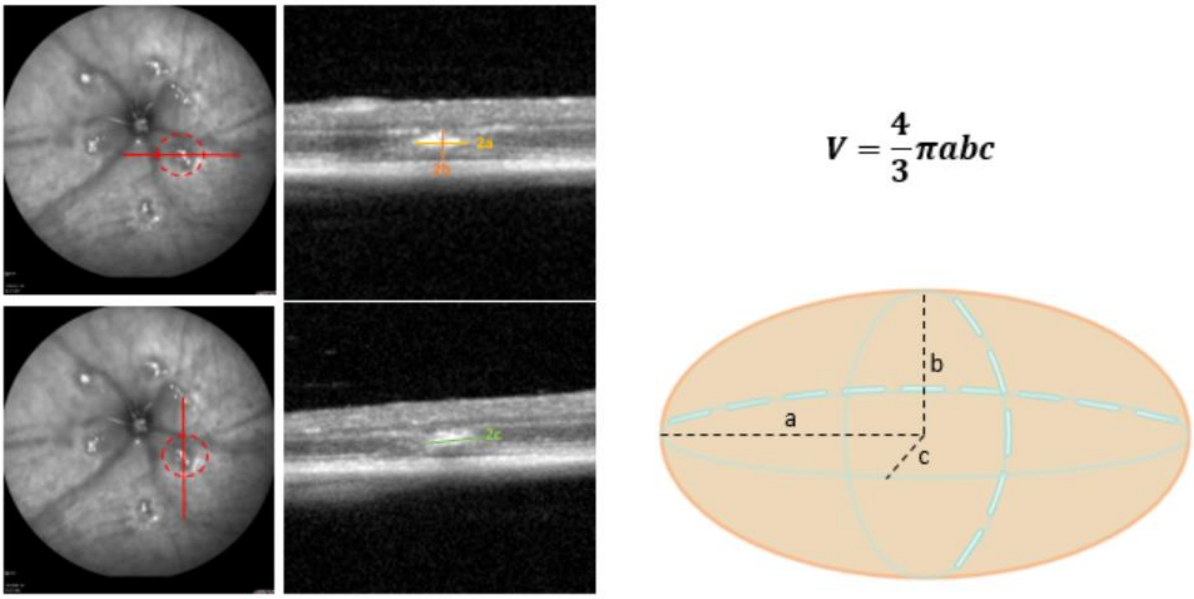

$$V = \frac{4}{3}\pi abc$$

B)

- **Grade-I**, no hyperfluorescence;
- **Grade-II**, hyperfluorescence without leakage;
- **Grade-III**, hyperfluorescence in the early or mid-transit images and late leakage;
- **Grade-IV**, bright hyperfluorescence in the transit images and late leakage beyond the treated areas.

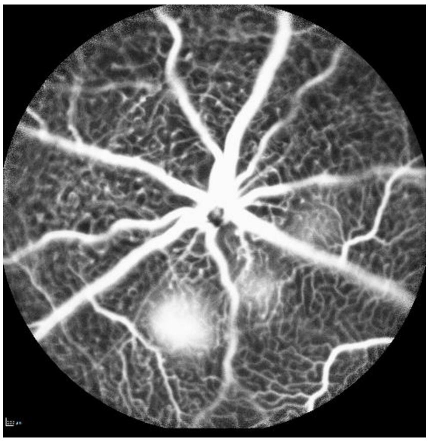

Figure 1: **(A)** Schematic diagram of measuring lesion size and calculating the volume of an ellipsoid. **(B)** FA image leakage grading criteria.

ROCK treatment

Fasudil was administered intraperitoneally (i.p.) at a concentration of 20 mg/kg in Hank's balanced salt solution (HBSS; Sigma-Aldrich, Burlington, Massachusetts, United States) once daily from d35 to d49 post laser. Belumosudil was administered i.p. at a concentration of 10 mg/kg in a 1:9 solution of dimethyl sulfoxide (DMSO; Sigma-Aldrich) and corn oil (Sigma-Aldrich) twice daily at 12-hour intervals from day 35 to day 49 post laser to treat solely fibrosis without active CNV.

Cell culture

Primary human dermal fibroblasts (ATCC, PCS-201-012) were cultured at 37 °C in a humidified incubator with 5% CO_2 using Fibroblast Growth Medium (C-23220, Sigma-Aldrich, Cambridge, MA, USA) supplemented according to the situation. MC3T3-E1 cells (99072810-DNA-5UG, Merck) were maintained in α-MEM medium (12492013, Gibco, Grand Island, NY, USA), supplemented as recommended. Cells were detached with 0.05% Trypsin-EDTA and adjusted to $0.5X10^5$ cells/ml (~80% full). The suspension was centrifuged at 1500 rpm at room temperature for 5 min. Supernatant was discarded and the precipitated cells were re-suspended with supplement-free culture medium and transferred to a laminin coated 8-wells chamber slide (Sigma-Aldrich) for 24 hours.

H&E, IHC

HE Images were captured with a Leica microscope (DMRBE; Leica, Wetzlar, Germany). IHC Images were taken with a fluorescence microscope (Eclipse Ni 90; Nikon, Egg, Switzerland).

Westernblot

The eyes were enucleated, the choroid-RPE complex was isolated by microsurgery, and the total protein was extracted using RIPA buffer (88901; Thermo Fisher Scientific) with a protease inhibitor cocktail. Individual sample protein concentrations were determined by the Bradford method. The lysed samples were then homogenized using a Precellys 24 tissue homogenizer (Bertin Instruments, Montigny-le-Bretonneux, France) and centrifuged twice at 5.500g at 4°C for 1 min. The supernatant was transferred to a new Eppendorf tube and centrifuged at 13.000 rpm, 4°C for 20 min. Then the supernatant was transferred to a new tube and centrifuged again for 10 min. Finally, supernatants were collected and stored at -80°C. The absorbance of samples with unknown protein concentration was measured at 600 nm and the protein concentration was obtained from the standard curve equation. After adjusting the samples according to the standard curve equation, the mixture loading solution containing samples (34 µg/ml), laminin blue and RIPA buffer, were separated by SDS-PAGE and electroblotted onto Trans-Blot Turbo Mini 0.2 µm nitrocellulose transfer packed membrane (1704-158; Bio-Rad, Cressier, Switzerland). To block non-specific signals, the membranes were immersed in blocking buffer TBS (LIC-927-60001; LI-

COR, Bad Homburg, Germany) at room temperature for 1 h. The membranes were incubated individually with rabbit or mouse antibodies on a shaking bed at 4°C overnight. This was followed by incubation with 2nd antibodies. The signals were visualized by fluorescence on the Odyssey Infrared Imaging System (LI-COR). (antibody list reported in Supplement tables/files S13).

Flatmount

Retinal flatmount preparation involves enucleating the eye, fixing it briefly in 4% paraformaldehyde, and carefully dissecting out the retina under a microscope. After removing the cornea, lens, and iris, the retina is isolated and optionally post-fixed. For immunostaining, the tissue is permeabilized with Triton X-100, blocked, and incubated with primary and secondary antibodies. Four radial cuts are made to flatten the retina onto a microscope slide, with the ganglion cell layer facing up. It is then mounted with an antifade medium, sealed with a coverslip, and imaged under a fluorescence or confocal microscope. Staining: CNV (isolectin Isolectin GS-IB4; Thermo Fisher Scientific) and fibrosis (anti-collagen I; Abcam, Cambridge, United Kingdom).

IMC

Data were processed as described in the Steinbock pipeline, except for cell segmentation masks being produced using the cellpose tissue net model. For the segmentation model, the flow threshold parameter was set to 1.0, cellprob_threshold to -3.0 and the expected cell diameter to 6. Channels DNA1 and DNA2 were chosen to represent nuclear and all other non-background membrane and cytoplasm channels. For dimensionality reduction and clustering assays, the seed was set to 322 prior to every calculation.

Minor regions of interest (ROI) LP 1-3 were used for Hyperion laser power tests and were discarded from analysis. Informative channel dual counts were arcsinh-transformed. Transformed counts were used for dimensionality reduction using the R package scater v.1.28.048 runUMAP function with default parameters.

During quality control, cells in the choroid-RPE layer were observed to be positive for the majority of markers used in this study. To not interfere with other cell type annotations, they were manually labelled using unsupervised clustering. Cells from the whole object were clustered using the R package CATALYST v.1.24.049 cluster function with default parameters using all informative channels. Separation into two

clusters was chosen and based on overall high marker intensity cells belonging to cluster 1 were annotated as choroid-RPE complex.

The remaining cells were phenotyped manually where possible for each ROI (Supplementary tables/files S3). Using markers associated with perivascular cells, microglia, macrophages, T cells, and B cells were annotated using an in-house app. To confirm the annotation, an unsupervised clustering of manually annotated cells using all informative channels was performed using the CATALYST cluster function with default parameters. Separation into eight clusters was chosen in this step. Based on marker levels, cells in cluster 1 were assigned to be perivascular (high α-SMA), in cluster 2 – microglial (high CD11b), cluster 3 – choroid-RPE complex (high overall), cluster 4 – macrophages (high CD11b and CD45), and clusters 5-8 – choroid-RPE complex (high overall). Low amounts of cells expressing T and B cell markers were found during manual annotation (Supplementary tables/files S4), but clustered closely with choroid-RPE cells both spatially and on dimensionality reduction graphs and as such were re-labelled as belonging to that layer.

The remaining unannotated cells were analyzed for increased vimentin and CD44 marker levels. Undefined cells were re-clustered using all informative channels as described above. Separation into seven clusters was selected and cells from cluster 4 with elevated vimentin levels were assigned as vimentin-positive.

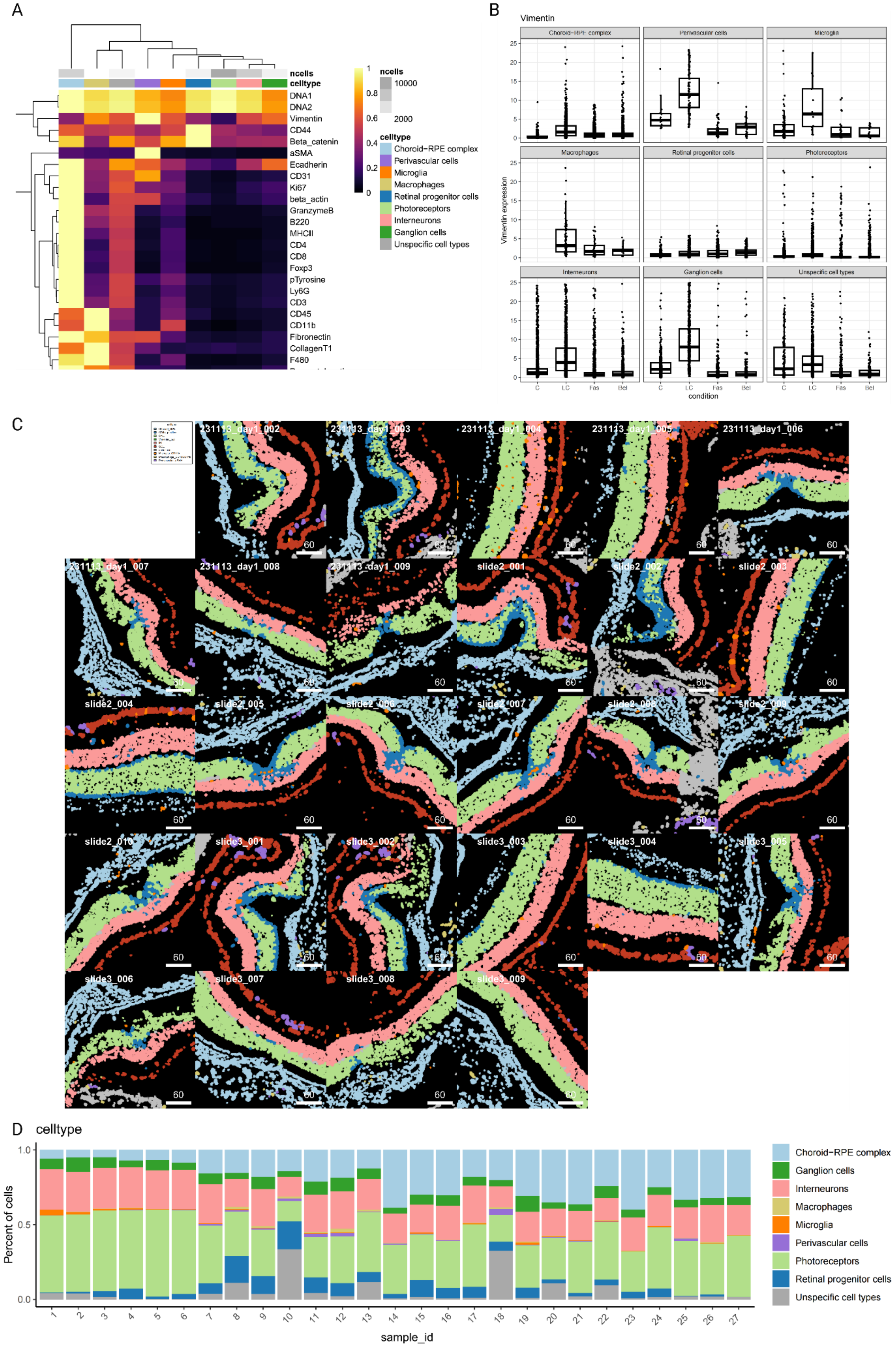
A
ncells
celltype
DNA1
DNA2
Vimentin
CD44
Beta_catenin
aSMA
Ecadherin
CD31
Ki67
beta_actin
GranzymeB
B220
MHCII
CD4
CD8
Foxp3
pTyrosine
Ly6G
CD3
CD45
CD11b
Fibronectin
CollagenT1
F480
1
0.8
0.6
0.4
0.2
0
ncells
10000
2000
celltype
Choroid−RPE complex
Perivascular cells
Microglia
Macrophages
Retinal progenitor cells
Photoreceptors
Interneurons
Ganglion cells
Unspecific cell types
B
Vimentin
Choroid−RPE complex
Perivascular cells
Microglia
Macrophages
Retinal progenitor cells
Photoreceptors
Interneurons
Ganglion cells
Unspecific cell types
Vimentin expression
C LC Fas Bel
condition
C
231113_day1_002
231113_day1_003
231113_day1_004
231113_day1_005
231113_day1_006
231113_day1_007
231113_day1_008
231113_day1_009
slide2_001
slide2_002
slide2_003
slide2_004
slide2_005
slide2_006
slide2_007
slide2_008
slide2_009
slide2_010
slide3_001
slide3_002
slide3_003
slide3_004
slide3_005
slide3_006
slide3_007
slide3_008
slide3_009
60
D
celltype
1.0
0.5
0.0
Percent of cells
1 2 3 4 5 6 7 8 9 10 11 12 13 14 15 16 17 18 19 20 21 22 23 24 25 26 27
sample_id
Choroid−RPE complex
Ganglion cells
Interneurons
Macrophages
Microglia
Perivascular cells
Photoreceptors
Retinal progenitor cells
Unspecific cell types

Figure 2: **(A)** Heatmap of key protein markers across annotated retinal cell types. Unspecific cell types show elevated fibrotic markers (fibronectin, type 1 collagen). **(B)** Boxplots of Vimentin expression across four conditions (C, LC, Fas, Bel) by cell type. **(C)** Spatial maps of classified cell types across retinal sections. **(D)** Cell type composition by sample.

This IMC-based spatial profiling of the retina reveals striking changes in cell type composition and protein expression in response to pathological conditions (e.g., Fas, Bel). A distinct population of unspecific cells, localized near the choroid–RPE interface, shows elevated levels of fibrotic/mesenchymal markers such as Vimentin, αSMA, CollagenT1, and Fibronectin. These findings supports that this population may consist of fibroblasts, myofibroblasts, or RPE cells undergoing epithelial–mesenchymal transition (EMT).

The co-localization of fibrotic markers with spatial niches of damage, along with an increase in immune cells (macrophages, microglia), supports the presence of fibroinflammatory remodeling in disease-affected retinal zones.To isolate CD44-positive cells, an unsupervised clustering of the whole dataset was performed using all informative channels. Separation into 20 clusters was selected and undefined cells from cluster 5 with elevated CD44 levels were assigned as CD44-positive. To resolve the remaining unannotated cells manual labelling of retinal layers was performed. Binary masks obtained from the segmentation step were imported into qupath v. 0.5.050. Masks belonging to choroid-RPE, outer nuclear layer (ONL), inner nuclear layer (INL), and ganglion cell layer (GCL) were manually highlighted and transferred to the dataset (Zenodo link). Undefined cells and cells mistakenly assigned to the choroid-RPE complex due to tissue folds within these defined layers were then renamed after them. The undefined cells were identified with type 1 collagen and fibronectin, left as unspecific. Differential abundance testing was performed using the edgeR R package. The masked_roi_alternative contains R-generated output from a cell-type segmentation analysis of retinal or ocular tissue, likely part of a spatial transcriptomics or immunohistochemistry study. It documents annotated regions corresponding to key retinal layers (ONL, INL, GCL), structural cells (Choroid_RPE, Vimentin-positive), and immune cell populations (CD44-positive, Microglia_CD11b, Macrophage_CD45CD11b, Perivascular_aSMA). Each slide or image (e.g., 231113_day1_002, slide2_001, etc.) is associated with the number "60," possibly indicating the number of detected regions or standardized ROI threshold. The presence of an "undefined" category suggests some segmentation ambiguity or unclassified regions. This summary indicates a systematic spatial classification of retinal cells, useful for studying tissue architecture or inflammation.

References:

1. Gong, Y. *et al.* Optimization of an Image-Guided Laser-Induced Choroidal Neovascularization Model in Mice. *PLoS ONE* **10**, e0132643 (2015).

2. Sulaiman, R. S. *et al.* A Simple Optical Coherence Tomography Quantification Method for Choroidal Neovascularization. *J. Ocul. Pharmacol. Ther.* **31**, 447–454 (2015).

3. Schindelin, J. *et al.* Fiji: an open-source platform for biological-image analysis. *Nat. Methods* **9**, 676–682 (2012).
