# Supplementary figures and images for: Rho-kinase inhibition reduces subretinal fibrosis
Source: Cell Death Discov. 2025 Oct 6;11:428. doi: 10.1038/s41420-025-02709-0 (PMC12501381; doi:10.1038/s41420-025-02709-0)

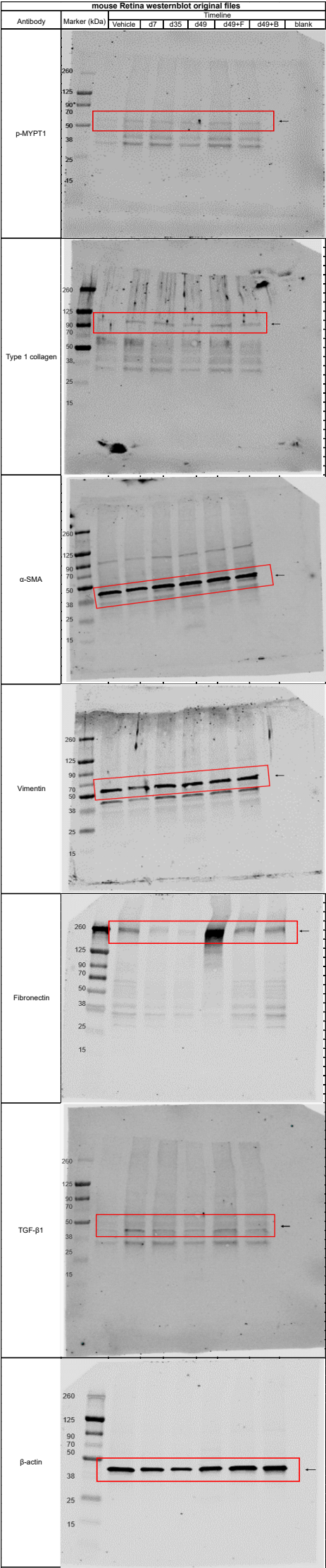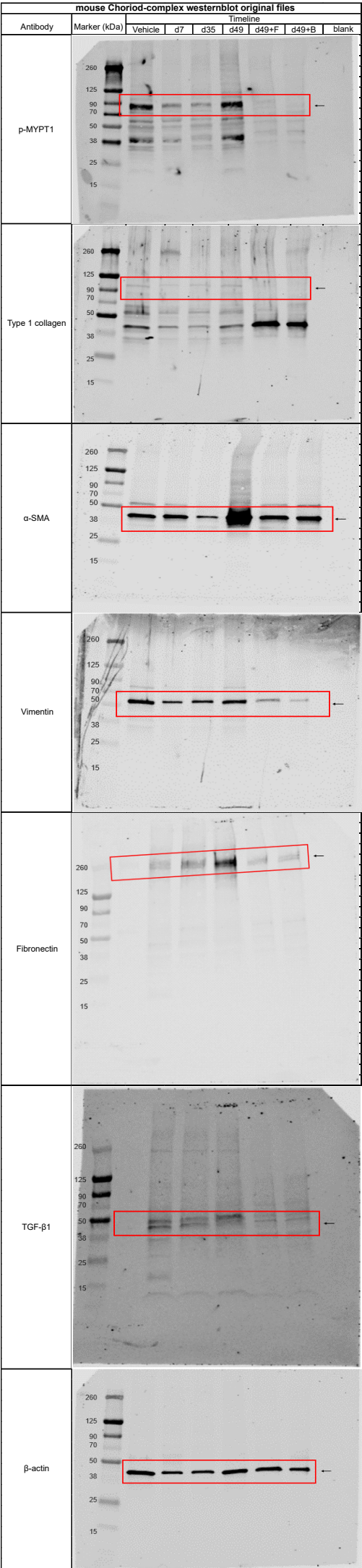

Supplement: Supplementary file 3 — Supplementary-westernblot original files [file 41420_2025_2709_MOESM3_ESM.pdf]
